# Selected Abstracts from the 2019 NCRI Cancer Conference of National Cancer Research Institute

**DOI:** 10.1038/s41416-019-0555-x

**Published:** 2019-10-04

## Cancer discovery and underpinning research

### 1. A novel role of BAP1 in regulating the spindle assembly checkpoint and mitotic spindle assembly

#### Anita Singh^1^, Dean Fennell^1^, Andrew Fry^1^

##### ^1^University of Leicester, Leicester, UK

**Background:** BRCA1 associated protein 1 (BAP1) is a tumour suppressor that plays critical roles in cell cycle regulation, transcription, cell death and DNA damage repair. Germline or somatic mutations and copy number alterations have been identified in several cancers and inactivation of BAP1 accelerates invasion, recurrence and metastases. BAP1 loss can modulate the response to chemotherapeutic drugs. However it is unknown how BAP1 status modulates the response to the DNA damaging agents or anti-microtubule drugs. The aim of this study was to identify how BAP1 regulates the DNA repair, SAC and mitosis.

**Method:** Functional genetic analysis of SAC proteins was conducted after BAP1 depletion using siRNA transfection. Spindle length and aster volume as measured in Imaris3D. Stable cell lines with wildtype or mutants of BAP1 were irradiated to induce DNA damage.

**Results:** Loss of BAP1 led to mitotic spindle defects such as elongated spindle length, increased aster volume by regulating the localization and expression of motor proteins, KIF18A and KIF18B. BAP1 is required to maintain SAC integrity through stimulating MAD2 expression and BUBR1 recruitment to kinetochores. Expression of wild type and mutants of BAP1 increased BRCA1 foci after DNA damage.

**Conclusion:** The findings suggest a novel role for BAP1 in regulating mitotic spindle assembly and regulating genomic stability. Our findings also suggest that during DNA damage BAP1 is required to recruit BRCA1 foci for DNA repair. Our results also suggest that the association between BAP1 and SAC may be clinically relevant. Vinorelbine exhibits useful clinical activity in mesothelioma and is currently being evaluated in a multicentre randomised phase II trial (VIM, NCT02139904) and patients are currently screened for BAP1 status for MIST trial. BAP1 has the potential as a predictive biomarker for chemotherapeutic drugs, to underpin chemotherapy stratification.

**Disclosure:** Funded by University of Leicester, Leicester, UK

**Corresponding author:** Anita Singh

### 2. Inhibiting Ehmt2 and Ezh2 histone methyltransferases alters the immune microenvironment in a *Trp53*^-/-^ murine ovarian cancer model

#### Pavlina Spiliopoulou^1^, Sarah Spear^2^, Susanne Dowson^3^, Susan Mason^3^, Karen Blyth^3^, Matthew Fuchter^2^, Bob Brown^2^, Iain A McNeish^2^

##### ^1^Beatson West of Scotland Cancer Centre, Glasgow, UK, ^2^Imperial College London, London, UK, ^3^University of Glasgow, Glasgow, UK

**Background:** Ovarian cancer prognosis is strongly dependent on the development of an anti-tumour immune response. However, tumours can epigenetically silence immunostimulatory genes in order to evade this response. We investigated whether a novel dual inhibitor of Ehmt2/Ezh2 methyltransferases (HKMT) was able to derepress expression of critical chemokines and augment immune responses in a murine ovarian cancer model.

**Method:** ID8 *Trp53*^-/-^ murine ovarian cancer cell line was previously generated, using CRISPR-Cas9 technique. Mice bearing intraperitoneal *Trp53*^-/-^tumours were treated with the novel Ehmt2/Ezh2 inhibitor, HKMTI-1-005, for 14 days (21-35d). Tumours were harvested for immune cell phenotyping by flow cytometry. HKMT1-1-005 was screened in vitro for its ability to enhance expression of 84-chemokine genes in ID8 *Trp53*^-/-^ ovarian cancer cells.

**Results:** In vitro, HKMTI-1-005 treatment significantly (p<0.05) upregulated the expression of cxcl10 (3-fold), cxcl9 (22-fold) and ccl5 (14-fold), after stimulation with IFNγ. Mice treated with HKMTI-1-005 had longer survival (52 *vs*45d,p<.0001), less ascites (3.7 *vs*5.6ml,p=.0037) and trended towards tumour size reduction (weight 138 *vs*178mg,p=.10) compared to vehicle treatment. Tumours harvested 24hr post last HKMTI-1-005 dose had significantly more effector CD8^+^T cells (p=.03), natural killer (NK) cells (p<.0001) and dendritic cells (DCs, p=.02), and less naïve CD8^+^T cells (p=.02) and immunosuppressive CD4^+^Tregs (p=.02). Expression of the Cxcl9/Cxcl10 receptor Cxcr3 was increased in HKMTI-1-005-treated cohort tumours on CD8^+^[mean fluorescence intensity (MFI) 3959 *vs*2097, p<.0001], CD4^+^(MFI 2341 *vs*1099, p<.0001)and NK (MFI 1507 *vs*440, p<.0001) cells.

**Conclusion:** Inhibition of Ehmt2/Ezh2 HKMTs stimulates expression of chemokines involved in T cell, NK and DC recruitment. In vivo, HKMTI-1-005 alters the immune microenvironment and confers a small survival benefit. This suggests that HKMTI-1-005 could augment the anti-tumour immune response of current immunotherapies.

**Disclosure:** Funded by Imperial College London, London, UK

**Corresponding author:** Pavlina Spiliopoulou

### 3. Pick-Seq®: a novel technology to retrieve tissue micro-regions for RNA sequencing

#### Rebecca Podyminogin^1^, Nolan Ericson^1^, Jennifer Chow^1^, Jia-Ren Lin^2^, Yu-An Chen^2^, Zoltan Maliga^2^, Kyla Teplitz^1^, Tessa Pei-Ching Tsai^1^, Adrian Quintanilla^1^, Peter Sorger^2^, Eric Kaldjian^1^, Tad George^1^

##### ^1^RareCyte, Seattle, US, ^2^Harvard Medical School, Cambridge, US

**Background:** Tumour tissue imaging allows for contextual understanding of tumour cells in the immune microenvironment. Pick-Seq combines high-resolution multi-parameter imaging with micro-region retrieval for RNA sequencing.

**Method:** Frozen breast carcinoma and formalin-fixed, paraffin-embedded tonsil sections were stained with immunofluorescence (IF) for cytokeratin and B and T cell markers. CyteFinder® Imaging System performed whole-slide scanning. 40μm diameter micro-regions were retrieved with CytePicker® Retrieval Module. RNA was amplified, sequenced, and differential gene expression analysis was performed. Identified genes were selected for Pick-Seq-informed IF panels. Cell compositions of each micro-region were deconvolved with CIBERSORT.

**Results:** Breast cancer micro-regions containing T cells, tumour cells, and tumour-infiltrating lymphocytes were retrieved for RNA sequencing. CIBERSORT showed an association between T-cell quantity observed by IF imaging and relative abundance of T-cell RNA. Tonsil micro-regions from one T-cell zone and two adjacent follicles were retrieved for RNA sequencing. Hierarchical clustering of differentially expressed genes confirmed B-cell markers in follicles and T-cell markers in the T-cell zone. CIBERSORT revealed distinct cellular compositions between T-cell zones and the B-cell follicles. Principle component analysis of gene expression found that micro-regions picked from the two follicles clustered independently from each other, and from the T-cell zone micro-regions. Fragments per kilobase million analysis revealed differing expression of genes between the adjacent follicles. Staining confirmed differential protein expression, indicating that only one follicle contained a germinal center in the section.

**Conclusion:** We demonstrate high-resolution multi-parameter imaging of both frozen and FFPE sections for retrieval of micro-regions for transcriptomic analysis using Pick-Seq. RNA expression analysis differentiated the tumour and T cell micro-regions in breast carcinoma, and between follicle and T-cell areas in tonsil and uncovered differences between adjacent follicles that were confirmed by IF imaging. These data demonstrate the power of Pick-Seq as a tool for spatially-focused RNA sequencing and biomarker discovery.

**Disclosure:** Funded by RareCyte, Seattle, US, Harvard Medical School, Cambridge, US

**Corresponding author:** Tessa Pei-Ching Tsai

### 4. Phenotype dictates outcome in synchronously resected primary colorectal tumours and matched liver metastases

#### Kathryn Pennel^1^, Colin Steele^1^, Antonia Roseweir^1^, Rene Jackstadt^2^, Colin Nixon^2^, Xabier Cortes-Lavaud^2^, Campbell Roxburgh^1^, Hester van Wyk^1^, Donald McMillan^1^, Paul Horgan^1^, Owen Sansom^2^, Joanne Edwards^1^

##### ^1^University of Glasgow, Glasgow, UK, ^2^Cancer Research UK Beatson Institute, Glasgow, UK

**Background:** 5-year survival of patients with resectable colorectal liver metastases is 25-40%. These patients do not generally benefit from immunotherapy although the majority are MMR proficient. The view that high inflammatory infiltrate confers better prognosis, is overly simplified and it is likely the balance of the immune cells influences outcomes. As neutrophils are associated with poor prognosis and many express chemokine receptor CXCR2, we hypothesised that CXCR2-positive neutrophils drive stromally dense phenotypes resulting in poor outcome in some patients.

The current study examines, in-depth the relationships between, phenotype, cellular infiltrate, tumour cell signalling, clinicopathological features and outcomes in primary and secondary colorectal cancer.

**Method:** A unique cohort of synchronously resected primary colorectal tumours and matched liver metastases (n=46) were stained for markers of immune cell infiltration (CD4, CD8, CD68, MPO) and inflammatory signalling pathways (CXCL2, MMP9, HIF-1α, CRP) using immunohistochemistry. Tumours were phenotypically subtyped using Klintrup-Mäkinen grade (KM), tumour stroma percentage and Ki67 proliferation-index.

**Results:** Patients with immune phenotype (high KM grade) in both primary and secondary sites had the best prognosis and those with high intra-tumour-stroma exhibited worst prognosis (p=0.006). Presence of high levels of macrophages (CD68) associated with CXCR2 positive cells (p=0.018) and when these were both present in high numbers in both sites associated with stromal phenotype (p=0.021), tumour budding (p=0.002) and worse prognosis (p=0.002). Intra-tumour neutrophils (MPO) associated with CXCR2 expression(p=0.042). Macrophages, MMP9, and C-Reactive Protein expression increased between primary tumours and matched liver metastases, whereas lymphocyte infiltration (CD4, CD8), HIF-1α and CXCR2 expression was not observed to change.

**Conclusion:** We observed an increase in cancer-specific survival in patients with high immune cell infiltrate when compared with patients with high levels of intra-tumour stroma. Patients whose tumours exhibited this stromal phenotype had poor prognosis and were more likely to have infiltration of myeloid-cells including CXCR2-positive cells.

**Disclosure:** Funded by Medical Research Council, Swindon, UK

**Corresponding author:** Kathryn Pennel

### 5. Abstract withdrawn

### 6. Role of cholesterol in colon cancer and its impact on AOM/DSS induced mouse intestinal tumourigenesis

#### Shyamananda Singh Mayengbam^1^, Manoj Kumar Bhat^1^

##### ^1^National Centre for Cell Science, Savitribai Phule Pune University, Pune, India

**Background:** Clinical studies show a significant correlation in alteration of blood cholesterol level with colon cancer. Numerous studies have reported that blood cholesterol level often decreases in colon cancer patient, which is also negatively correlated with stage of tumour. We investigate the role of cholesterol in lipid and glucose metabolism of colon cancer along with various other signalling molecules.

**Method:** For understanding the role of cholesterol in colon cancer, long term colony formation, enzyme activity assay, glucose & lactate estimation were performed in HCT116 p53+/+ and p53-/- cells in the presence or absence of low density lipoprotein cholesterol (LDLc) and high density lipoprotein cholesterol (HDLc). Immunoblot and confocal microscopy for studying molecular events associated with cholesterol and colon cancer. C57BL/6J mice were used for in vivo isograft and chemically induced (AOM/DSS) colon cancer model.

**Results:** In vivo studies shows that mice fed on high cholesterol diet and high fat diet increases the incidence of AOM/DSS induced polyp formation, indicative of colon cancer when compared to mice fed with normal diet. Moreover, our in vitro result shows that, supplementation of LDLc and HDLc also increases colon cancer cell proliferation through ERK activation. We found that treatment of LDLc and HDLc increases glycolytic enzyme activities thereby enhancing glucose utilization and lactate production which in turns triggers the overexpression of monocarboxylate transporter 4 (MCT-4) for lactate efflux. Apart from increased enzyme activity, it also facilitates intracellular cholesterol accumulation and lipid droplet formation along with the up-regulation of LDL receptor (LDLR).

**Conclusion:** Both in vitro and in vivo results show a positive correlation of cholesterol with colon cancer cell proliferation and incidence. The role of cholesterol in lipid and glucose metabolism in colon cancer cells needs further investigation. Deciphering the underlying molecular mechanism of cholesterol associated events in colon cancer will help in better management of colon cancer.

**Disclosure:** Funded by Intramural grant from National Centre for Cell Science, Department of Biotechnology, Government of India.

**Corresponding author:** Manoj Kumar Bhat

### 7. Exploration of molecular signalling underpinning the DNA Damage Response Deficiency (DDRD) assay in Colorectal Cancer; Data from the S:CORT consortium (Stratification in COlorRecTal cancer)

#### Sudhir Malla^1^, Keara Redmond^1^, Andrew Blake^2^, Enric Domingo^2^, Susan Richman^3^, Michael Youdell^2^, Ian Tomlinson^4^, Louise Brown^5^, Tim Maughan^2^, Mark Lawler^1^, Philip Dunne^1^, on behalf of S:cort Consortium

##### ^1^Queen's University Belfast, Belfast, UK, ^2^University of Oxford, Oxford, UK, ^3^University of Leeds, Leeds, UK, ^4^University of Birmingham, Birmingham, UK, ^5^University College London, London, UK

**Background:** The DNA Damage Response Deficiency (DDRD) assay was developed by Almac (Craigavon, Northern Ireland, UK) as a predictive tool in breast cancer (BC) for response to DNA-damaging chemotherapy, based on tumour biology associated with Fanconi Anaemia/BRCA pathway loss. Following studies in BC, the S:CORT consortium tested the hypothesis that the DDRD assay might also predict response to oxaliplatin in colorectal cancer (CRC). In addition to the clinical endpoints, this study also set out to describe the underlying tumour biology represented by DDRD-positivity in CRC.

**Method:** A comprehensive data analysis was performed on 361 primary FFPE samples from a subset of the FOCUS metastatic CRC clinical trial, including assessment of consensus molecular subtypes (CMS), colorectal intrinsic subtypes (CRIS), DDRD calls, and DDRD scores. To explore the tumour microenvironment (TME) content and biology, MCPcounter (R package) and gene set enrichment analysis (GSEA) using the ‘Hallmark’ collection from genepattern was used. Additionally, 198 BC samples from TRANSBIG cohort (GSE7390), used in the development of DDRD assay, were included for a comparative analysis with CRC GSEA finding.

**Results:** CMS1 subtypes are proportionally higher in DDRD-positives and CMS4 in DDRD-negatives (Fisher’s exact test, P = 0.0002). Additionally, DDRD-positives are significantly infiltrated with cytotoxic lymphocytes (t-test, P < 0.0001) with a moderate linear correlation against DDRD scores (Pearson’s correlation r = 0.43598, P < 0.00001). Allograft rejection, interferon-α, and interferon-γ response are the top three upregulated gene-sets in DDRD-positives (FDR adjusted, P < 0.25). Of note, we observe CRC-specific upregulation of apical surface and spermatogenesis in DDRD-positives, and Hedgehog signalling and NOTCH signalling gene-sets in DDRD-negatives.

**Conclusion:** Our finding suggests DDRD-positive tumours represents a similar biology between the two cancer types in terms of TME infiltrates with high tumour-infiltrating lymphocytes and activation of interferon-related signalling. This distinct immune-specific DDRD biology suggests a potential immunotherapeutic implication in CRC.

**Disclosure:** Funded by Department for Education, London, UK, Cancer Research UK, London, UK, Medical Research Council, Swindon, UK

**Corresponding author:** Sudhir Malla

### 8. A potent synergy between FOXG1 overexpression and Wnt signaling drives cell cycle re-entry in quiescent glioblastoma stem cells

#### Faye Robertson^1^, Eoghan O'Duibhir^1^, Harry Bulstrode^2^, Steve Pollard^1^

##### ^1^University of Edinburgh, Edinburgh, UK, ^2^University of Cambridge, Cambridge, UK

**Background:** Glioblastoma is a malignant brain tumour which is universally fatal. Stem cells within the tumour exist in a quiescent state, evade destruction and reactivate, causing relapse. These stem cells are known to overexpress the transcription factor FOXG1.

**Method:** We use an in vitro model of quiescence in mouse neural stem cells, incorporating a doxycycline inducible human FOXG1 overexpression cassette, to identify, through high content pharmacological screening, a synergistic relationship between high FOXG1 expression and inhibition of glycogen synthase kinase 3 (GSK3) in driving cells into an active, proliferative state. We quantify this effect using EdU incorporation assays and demonstrate the high efficiency of cell cycle re-entry with colony forming assays. We use pharmacological and genetic approaches to demonstrate that the synergy is effected by canonical Wnt signaling and that it is relevant in human glioblastoma stem cells (GSCs).

**Results:** The effect of GSK3 inhibition can be phenocopied both by Wnt3a and by inducible constitutively active beta-catenin, suggesting that the synergy is effected through beta-catenin, the key downstream effector of canonical Wnt signaling. Furthermore, the combined effect of FOXG1 overexpression and GSK3 inhibition on exit from quiescence can be abrogated by Wnt inhibitors. This effect is present in patient-derived human GSCs and it is abolished by excision of FOXG1. Additionally, we show that the groucho-binding domain of the FOXG1 protein is necessary for the response of human GSCs to GSK3 inhibition, consistent with a putative mechanism whereby FOXG1 may sequester TLE1/groucho, a co-repressor at Wnt target genes. Finally, we have developed a mouse model of glioblastoma by excision of NF-1 and Pten and overexpression of EGFRvIII in cells with inducible active beta-catenin and FOXG1.

**Conclusion:** Targeting the synergistic relationship between FOXG1 and beta-catenin may provide an exciting therapeutic opportunity in preventing relapse and improving the prognosis of glioblastoma.

**Disclosure:** Funded by Cancer Research UK, London, UK

**Acknowledgement:** A version of this abstract has been published previously, see http://abstracts.ncri.org.uk/abstract/wnt-beta-catenin-synergises-with-foxg1-to-drive-exit-from-quiescence-in-neural-stem-cells-including-glioblastoma-stem-cells/

**Corresponding author:** Faye Robertson

### 9. 5-FU-based treatments: investigation in p53-proficient and -deficient colorectal cancer

#### Fiammetta Falcone^1^, Tamas Sessler^1^, Andrea Lees^1^, Gerard Quinn^1^, Peter Gallagher^1^, Daniel Longley^1^, Simon McDade^1^

##### ^1^Centre for Cancer Research & Cell Biology Queen's University Belfast, Belfast, UK

**Background:** Colorectal cancer (CRC) is one of the most common cancers. 5-fluorouracil (5-FU) remains the backbone treatment of CRC, which is most frequently combined with Oxaliplatin. However, 20-30% of patients relapse with a treatment-refractory disease, likely correlated to their molecular background. Mutations in TP53 are observed in at least 50% of all primary CRC. While TP53 status has been shown to influence Oxaliplatin response, correlation between 5-FU and p53 status is still not well understood.

**Method:** Using panel of p53 isogenic models of colorectal cancer, we conducted phenotypic and functional genomics analyses of the effects of 5-FU, and its combination with Oxaliplatin (clinically relevant doses). Integrative analysis of genome-wide 5-FU GeCKO CRISPR screen was performed to identified novel targets.

**Results:** In contrast to Oxaliplatin, 5-FU induced cell death is largely p53-independent. However, we observed that 5-FU causes dramatic increase in S-phase in p53-deficient cells, which coincides with increased DNA damage. This effect is not observed in p53-proficient cells due to cell cycle inhibitory effects of p21. Remarkably, we found that DNA damage and S-phase arrest are accompanied by a significant increase in expression of the immune-regulator programmed death-ligand 1 (PD-L1). GeCKO CRISPR screen in HCT116 p53 isogenic models identified Caspase-8 inhibitor CFLAR/FLIP as a negative regulator of both p53-dependent and -independent death. Interestingly, FLIP expression can be attenuated by Entinostat (Class I HDAC inhibitor). Importantly, we observed that combination of Entinostat with 5-FU significantly impacts long-term survival in a p53-independent manner, while its combination with Oxaliplatin enhances p53-dependent effects on cell death and long-term survival.

**Conclusion:** Our investigation supports two major novel combination treatments for 5-FU-based therapies correlated to p53-status. First, in p53-deficient patients, we propose the use of 5-FU-based treatments (5-FU, FOLFOX) in combination with anti-PD-L1 treatment. Second, in p53-proficient patients, we propose the combination of 5-FU or FOLFOX with class I Entinostat.

**Disclosure:** Funded by Centre for Cancer Research & Cell Biology Queen's University Belfast, Belfast, UK

**Corresponding author:** Fiammetta Falcone

### 10. Deciphering the molecular signalling underpinning early-dissemination and metastasis from stage I colorectal cancer

#### Philip Dunne^1^, Maurice Loughrey^2^, Helen Coleman^2^, Aoife McCooey^2^, Susan Richman^3^, Andrew Blake^4^, Keara Redmond^2^, Dion Morton^5^, Ian Tomlinson^5^, Simon Leedham^4^, Rachael McBride^2^, Jeffrey Campbell^2^, Tim Maughan^4^, Mark Lawler^2^, on behalf of S:cort Consortium (Stratification in COlorRecTal cancer)

##### ^1^Centre for Cancer Research & Cell Biology Queen’s University Belfast, Belfast, UK, ^2^Queen’s University Belfast, Belfast, UK, ^3^University of Leeds, Leeds, UK, ^4^University of Oxford, Oxford, UK, ^5^University of Birmingham, Birmingham, UK

**Background:** Reduction in colorectal cancer (CRC) mortality will largely involve prevention, earlier detection and treatment optimisation. Bowel cancer screening (BCS), detects asymptomatic tumours much earlier, with stage I CRC (T1/2, N0, M0) now accounting for 48% of BCS-detected tumours, compared to 9% outside screening. We hypothesise that within early lesions detected by BCS, there are highly aggressive early-disseminating tumours that would otherwise be detected at later stage, but due to BCS they are being caught earlier while potentially curable. We propose that molecular profiling can discriminate between lesions based on their probability of developing lymph node (LN+) and/or distant metastasis (dM+).

**Method:** To identify factors associated with early-dissemination, we have molecularly profiled a feasibility cohort (n=41) of T1 CRC, enriched for aggressive tumours (N+ and/or dM+ T1’s). Sample collection to expand this cohort to n=300 is ongoing.

**Results:** Histological factors (including immune/fibroblast content) that are prognostic in stage II/III CRC are not significantly associated with eventual metastatic relapse in T1 CRC. Molecular profiling revealed a significant shift in transcriptional signalling between aggressive and non-aggressive tumours. Intrinsic stem-like biology in combination with stress-response activation and, intriguingly, signalling normally associated with development of Parkinson’s disease is elevated in CRC tissue from aggressive lesions. Preliminary analysis with in vitro and in vivo models of CRC have confirmed the ability of these intrinsic factors to promote early-dissemination and metastatic spread, which reflects the aggressive clinical phenotype.

**Conclusion:** In order to find any effective treatment you have to first understand the biology underpinning disease. Our data indicates that the ability to undergo early-dissemination, leading to either LN+ or dM+, is established and, most importantly, detectable, even in the earliest pathologically staged T1 tumours. This study may ultimately enable the development of tailored disease management plans for the increasing numbers of early-stage patients being diagnosed as a result of BCS.

**Disclosure:** Funded by Cancer Research UK, London, UK, Medical Research Council, Swindon, UK

**Corresponding author:** Philip Dunne

### 11. The p53/p21 axis suppresses 5FU-induced, ATM/ATR-STAT3-mediated activation of PD-L1

#### Tamas Sessler^1,2^, Fiammetta Falcone^2^, Peter Gallagher^2^, Daniel Longley^2^, Simon McDade^2^

##### ^1^Centre for Cancer Research & Cell Biology, Forensic Geoscience Group Queen's University Belfast, Belfast, UK, ^2^Queen's University Belfast, Belfast, UK

**Background:** The immune system plays an important role in the response to chemotherapeutic treatments and there is increasing evidence to suggest that this is at least in part due to upregulation of PD-L1. Elevated expression of PD-L1 has been shown to promote cancer cell survival as PD-L1 is able to inactivate T-cells, inhibiting their activity, proliferation and promoting apoptosis. Given the importance of 5FU in cancer treatment, little is known about its effect on PD-L1 expression.

**Method:** HCT116, LoVo and RKO with/without p53 were treated with 5FU alone/in combination with oxaliplatin and FUdR for 48h. PD-L1 expression was measured at mRNA level, immunoblotting and surface staining. Cells were screened using different siRNA with 5FU treatment. Involvement of the DNA damage pathway was assessed by ATM, ATR and CHK1 inhibitors and siRNAs.

**Results:** 5FU caused an increased S-phase arrest and a more sustained activation of the DNA damage repair pathway in the absence of p53 or p21. PD-L1 expression increased upon 5FU treatment which was more pronounced in p53-/- or p21-/- cells. By using FUdR, a metabolite of 5FU that acts through inhibition of thymidylate synthase (TS), we were able to reproduce the effect of 5FU on PD-L1 expression. Thymidine depletion can lead to stalled replication fork that in the absence of p53/p21 axis, cause elongated activation of ATR and ATM pathway. Inhibiting ATM/ATR by small molecule can reduce PD-L1 upregulation. Using siRNA we identified STAT3 as a key regulator of the 5FU-mediated PD-L1 upregulation.

**Conclusion:** Upon 5FU treatment, we demonstrated a marked PD-L1 upregulation in CRC models in the absence of p53 or p21. This effect is caused by inhibition of TS resulting in prolonged ATM pathway activity and in turn by STAT3 activation. This would indicate a possible therapeutic window using PD-1/PD-L1 inhibitors in p53 negative CRC with 5FU treatment.

**Disclosure:** Funded by Cancer Research UK, London, UK

**Acknowledgement:** A version of this abstract has been published previously, see http://abstracts.ncri.org.uk/abstract/the-molecular-mechanism-behind-pd-l1-upregulation-upon-5-fluorouracil-treatment-in-colorectal-cancer-cells/

**Corresponding author:** Tamas Sessler

### 12. Loss of BCL-3 expression increases sensitivity to irradiation induced DNA damage in colorectal cancer cells

#### Christopher Parker, Adam Chambers, Ann Williams

##### ^1^University of Bristol, Bristol, UK

**Background:** In locally advanced rectal cancer, pre-operative long-course chemoradiotherapy (LCCRT) is an important addition to surgical resection, however up to 40% of patients do not respond. The proto-oncogene BCL-3 is upregulated in a subset of colorectal cancer patients and expression is associated with poorer patient outcome. We aimed to investigate whether BCL-3 expression confers resistance to DNA damaging agents used in LCCRT to determine whether BCL-3 is a useful marker for predicting patient response or as a target in therapy.

**Method:** BCL-3 expression in colorectal cancer cell lines was knocked down by siRNA (2D) and knocked out by CRISPR-Cas9 D10A based genome editing (3D). Cells were irradiated with a Caesium-137 source and responses were measured by crystal violet staining, γ-H2AX foci imaging, Western blotting and DNA damage repair reporter assays.

**Results:** Suppression of BCL-3 expression increases irradiation induced cell death in colorectal cancer cells. This is accompanied by an increased number of double strand breaks as shown by γ-H2AX foci and upregulation of double strand break dependent signalling (ATM and Chk2 phosphorylation). Reporter assays demonstrate that the increase in irradiation induced double strand breaks on loss of BCL-3 expression is due to a significant reduction in the rate of homologous recombination.

**Conclusion:** This study demonstrates a novel role for BCL-3 in promoting resistance to DNA damaging agents in colorectal cancer. The results indicate that increased BCL-3 expression seen in some colorectal cancer could aid homologous recombination allowing more efficient repair of DNA after damage, enhancing tumour survival. This suggests that targeting BCL-3 during rectal cancer treatment could enhance response to LCCRT.

**Disclosure:** Funded by Wellcome Trust, London, UK, Medical Research Council, Swindon, UK

**Corresponding author:** Christopher Parker

### 13. classifieR: an interactive web interface for the molecular classification of colorectal cancer from RNA-sequencing data

#### Gerard Quinn^1^, Tamas Sessler^1^, Wendy Allen^1^, Sarah Maguire^1^, Phillip Dunne^1^, Darragh McArt^1^, Harper VanSteenhouse^2^, Peter Gallagher^1^, Andrea Lees^1^, Dan Longley^1^, Bruce Seligmann^2^, Mark Wappett^1^, Simon McDade^1^

##### ^1^Queen's University Belfast, Belfast, UK,^2^BioSpyder Technologies, Carlsbad, US

**Background:** Colorectal cancer (CRC) is the 3rd most common form of cancer worldwide with ~700,000 deaths per year. Next generation sequencing (NGS) is leading the drive towards personalised precision cancer medicine. However, problems with data analysis of Next Generation Sequencing (NGS) data present difficulties in translating research into clinical assays. Currently colorectal patient samples can be stratified into distinct molecular subgroups based on gene expression, however this requires an experienced bioinformatician, this bottleneck can additionally prevent the adoption of these classifiers in the clinic.

**Method:** classifieR is developed in R with Shiny. Available for both Windows and MACOS to handle larger datasets. classifieR allows users to upload their own RNA sequencing data and specifically modified R packages CMSclassifier, CRISclassifier, DoRoTheA and MCP-counter are run in the backend.

**Results:** We developed the classifieR app, a dynamic and interactive web interface which allows for the characterization of RNA sequencing data into molecularly defined subtyping algorithms of colorectal cancer - providing CRIS subgroup, CMS subtype, an estimation of immune populations and transcription factor activity for each sample. The app can normalize raw count data through DeSeq2 and generate CRIS, CMS, MCP-counter scores and DoRothEA transcription factor activity scores for a large number of samples in a short space of time. Data can also be visualized on each page.

**Conclusion:** classifieR provides a framework which enables labs without access to a dedicated bioinformation to get information on the molecular makeup of their samples, providing an insight into patient prognosis and druggability. This application is freely available online.

**Disclosure:** Funded by Department for Education Northern Ireland, BioSpyder Technologies, Carlsbad, US

**Corresponding author:** Gerard Quinn

### 14. The gut microbiome and response to neoadjuvant chemotherapy in breast cancer

#### Kirsty Ross^1^, Rodanthi Papadopoulou^2^, Ben Nichols^2^, Martin Macleod^1^, Judith Fraser^1^, Sophie Barrett^1^, Jeff Evans^2^, Konstantinos Gerasimidis^2^, Iain Macpherson^1,2^

##### ^1^Beatson West of Scotland Cancer Centre, Glasgow, UK, ^2^University of Glasgow, Glasgow, UK

**Background:** Pre-clinical data suggest that the activity of cytotoxic chemotherapy may be dictated by the gut microbiome. Pathological complete response (pCR) to neoadjuvant chemotherapy (NACT) predicts improved event-free and overall survival in patients with all breast cancer subtypes. This prospective study investigates the compositional and functional changes in the gut microbiome during NACT and explores correlations with pathological response.

**Method:** Female patients receiving NACT for breast cancer at the BWoSCC were enrolled from Aug2017-Mar2019. Stool samples were collected at 3 timepoints: 1) before 1^st^ cycle NACT, 2) during NACT, 3) after final cycle of NACT. DNA was extracted and the V4 region of the 16S rRNA gene was amplified for 2x250 bp next-generation sequencing. After surgery each patient’s response was categorised as pCR (ypT0/is ypN0) or non-pCR.

**Results:** To date 21 patients have been recruited (18 currently evaluable.) Median age and BMI were 56 (range 33-72) and 28.6 kg/m^2^(range 19.6-55.6) respectively. Most (n=9, 42.9%) had HER2+ cancers whilst 8 (38.1%) had TNBC and 4 (19%) had (ER+HER2-). NACT included FEC-T in 9 (42.9%), FEC-TH in 6 (28.6%) and other regimes in 6 (28.6%). pCR was observed in 4 of 18 evaluable patients (22.2%.) Taxonomic richness was non-significantly higher in responders vs. non-responders at timepoints; 1) mean 515 vs. 367; p = 0.059, 2) mean 496 vs. 330; p = 0.061 and 3) mean 548 vs. 293; p = 0.069. In patients with 3 timepoint samples available (n=5), lack of response was associated with an increasing proportional abundance of Bacteroides, after completion of NACT (mean 0.57 vs. 0.13; p=0.038.)

**Conclusion:** Early data demonstrate non-significant trends in compositional differences in the gut microbiome structure of patients with pCR versus non-pCR. These include lower richness and higher proportional abundance of Bacteroides in the non-pCR group.

**Disclosure:** Funded by University of Glasgow, Glasgow, UK, Think Pink Scotland, Glasgow, UK

**Corresponding author:** Kirsty Ross

### 15. GYS1 inhibition and synthetic lethality with mitochondrial inhibitors in breast cancer

#### Ellen de Heer^1^, Christos E. Zois^1^, Syed Haider^2^, Daniel Ebner^1^, Andrew Ratcliffe^3^, Adrian Harris^1^

##### ^1^University of Oxford, Oxford, UK, ^2^Institute of Cancer Research, London, UK, ^3^Novintum Bioscience, London, UK

**Background:** Elevated glycogen levels and upregulation of key players in the glycogen metabolism are characteristics of various tumour types and associated with treatment resistance and poor clinical outcomes. We studied the role of glycogen synthase (GYS1) in breast cancer cell growth and metabolism, and evaluated its role in sensitivity to mitochondrial inhibitors.

**Method:** Gene expression of key players in glycogen metabolism (including GYS1, GYS2 and glycogen phosphorylase [PYGL, PYGB]) was analysed in the METABRIC dataset, containing genomic and transcriptomic profiles of 2000 breast cancer cases. Using siRNAs targeting GYS1, the impact of acute GYS1 knockdown on proliferation was studied in a broad panel of breast cancer cell lines and in a spheroid model of the triple-negative cell line MDAMB231. shGYS1 breast cancer cell lines were used for a synthetic lethality screening with approved anti-cancer drugs and mitochondrial inhibitors with various mitochondrial targets.

**Results:** High expression of GYS1 was correlated with poor survival in triple-negative breast cancer patients within the METABRIC data set. Downregulation of GYS1 significantly decreased cell growth of breast cancer cell lines BT474, SKBR3, MCF7, T47D, MDAMB453, MDAMB231, CAL51, MDAMB436, MDAMB468, HCC1143, HCC1937, HCC1806 (p<0.001, *n*=3) but not of SUM159PT. MDAMB231 spheroid growth was significantly impaired upon GYS1 inhibition (p<0.001, *n*=3). Finally, shGYS1 cancer cell lines were more sensitive to mitochondrial inhibitors (including gamitrinib-triphenylphosphonium and Novintum NBS037).

**Conclusion:** Inhibition of GYS1 decreases breast cancer cell proliferation and enhances the anti-cancer effect of mitochondrial inhibitors. Investigation of the underlying mechanism of action of this synthetic lethality is currently ongoing, and hypothesised to be related to changes in mitochondrial metabolism.

**Disclosure:** Funded by Breast Cancer Research Foundation, New York, US, Cancer Research UK, London, UK

**Corresponding author:** Ellen de Heer

### 16. Molecular and functional studies on a sub-population of T cells resistant to Galectin-9

#### Thi Bao Tram Tran^1^, Aurore Gelin^1^, Philippe Rameau^1^, Cyril Catelain^1^, Valentin Baloche^1^, Pierre Busson^1^

##### ^1^Gustave Roussy Institute, Villejuif, France

**Background:** Galectin-9 (Gal-9) is a member of the galectin family, which recognizes glycans containing β-galactoside bonds. Although lacking a secretion signal, Gal-9 is detected in the extracellular environment. It can be released by malignant cells and exhibit immunosuppressive effects, especially in nasopharyngeal carcinoma (Klibi et al., 2009, Blood), and melanoma (Enninga et al., 2016, Melanoma Res.). One aspect of gal-9 immunosuppressive effects is the induction of T-cell apoptosis. However, a fraction of T-cells from PBMCs appears to be consistently resistant to gal-9. Our aim is to investigate the phenotype and functional properties of this T-cell sub-population.

**Method:** In the current study, we applied a 7-day selection by Gal-9 treatment at 45 nM on PBMCs activated by anti-CD3/CD28 antibodies. The surviving T cell subsets were then stimulated by Gal-9, but at a lower concentration, followed by CyTOF and metabolomic analyses.

**Results:** Selected T cells were not only less prone to apoptosis but also retain a higher rate of cell proliferation and DNA synthesis. By using mass cytometry and meta-clustering algorithm, we observed an enrichment of CD4^+^ T cells co-expressing ICOS, CTLA-4, and PD-1, beside a regression of CD8^+^ T cells. We also found a subgroup of CD4^+^ T cells expressing CCR4 in the absence of FoxP3 suggesting a selective advantage for an atypical population of T-regs. The metabolomic profile of the selected subsets, on the other hand, showed significant increases in proline metabolism, putting forward a potential mechanism to explain their proliferative feature. Intriguingly, we also found an increased ratio of S-adenosylmethionine/methionine in selected cells, suggesting possible modifications in DNA methylation.

**Conclusion:** Overall, the profile of the surviving T cell subset gradually takes shape, suggesting a predominant suppressive activity. With a better understanding of how Gal-9 reshapes T cell population, we hope to elucidate how it contributes to immune evasion in tumour microenvironment.

**Disclosure:** Funded by Bristol-Myers Squibb Foundation, New York, US

**Corresponding author:** Thi Bao Tram Tran

### 17. A screen for epigenetic reprogramming reveals LSD1 inhibitor as a potential intervention to promote differentiation in pancreatic cancer

#### Frances Willenbrock^1^, James Wantling^1^, Eric O'Neill^1^

##### ^1^University of Oxford, Oxford, UK

**Background:** Pancreatic ductal adenocarcinoma (PDAC) responds poorly and rapidly gains resistance to conventional chemotherapeutics. Since this resistance may derive from proliferation of stem-cell like cells, inducing differentiation in these cells should increase their susceptibility to therapy. Epigenetic silencing of the set of genes responsible for differentiation commonly occurs and there is therefore potential to “reprogram” these cells by altering their epigenetic status. Our work looks at the effect of demethylase inhibitors in stabilising a more differentiated status in pancreatic cancer.

**Method:** We established a screen in which the PDAC cell line PSN1 was stably transfected with a reporter consisting of the RASSF1A promoter region linked to luciferase. Hypermethylation of the RASSF1A promoter is associated with adverse prognosis. This screening is therefore suitable as a proxy for detecting genome-wide demethylation events upon therapeutic intervention. Clones responding to treatment with the DNA methyltranferase-1 inhibitor, by increasing luciferase activity were further screened for synergy with known inhibitors of epigenetic modifiers. The effect of inhibitors on the differentiation status of parental PSN1 cells was validated by qPCR and western blotting.

**Results:** We identified lysine-specific demethylase 1 inhibitor, GSK-LSD1, as a positive hit in this screen. GSK-LSD1 increased expression levels of E-cadherin, claudin 1 and occludin mRNA and decreased Zeb2 mRNA expression. By optimising scheduling and media conditions we achieved durable responses in cellular differentiation status upon drug withdrawal, indicating we are rewiring the epigenome (e.g. gene specific DNA methylation, increased H3K4me2, and decreased H3K9me2) to stably alter cell phenotype.

**Conclusion:** Our screen is a rapid, sensitive method for detecting drug combinations that alter the epigenome in cancer cells. Use of GSK-LSD1 increased E-cadherin and other markers of differentiation, indicating that cells switch phenotype. Altering tumour cell phenotype to be well differentiated is likely to be an effective strategy to improve prognosis and responses to therapy.

**Disclosure:** Funded by Cancer Research UK, London, UK

**Corresponding author:** Frances Willenbrock

### 18. Prioritization of cancer therapeutic targets using CRISPR–Cas9 screens nominates the Werner Syndrome RecQ helicase as a synthetic lethal target in MMR-deficient tumours

#### Gabriele Picco^1^, Fiona M. Behan^1^, Francesco Iorio^1^, Emanuel Gonçalves^1^, Charlotte M. Beaver^1^, Giorgia Migliardi^2^, Rita Santos^3^, Yanhua Rao^3^, Francesco Sassi^2^, Marika Pinnelli^2^, Rizwan Ansari^1^, Sarah Harper^1^, David Adam Jackson^1^, Rebecca McRae^1^, Rachel Pooley^1^, Piers Wilkinson^1^, Dieudonne van der Meer^1^, David Dow^3^, Carolyn Buser-Doepner^3^, Andrea Bertotti^2^, Livio Trusolino^2^, Euan A. Stronach^3^, Julio Saez-Rodriguez^4^, Kosuke Yusa^5^, Mathew Garnett^1^

##### ^1^Wellcome Trust Sanger Institute, Cambridge, UK, ^2^Candiolo Cancer Institute, Strada Provinciale, Italy ^3^GlaxoSmithKline (GSK), London, UK, ^4^Institute for Computational Biomedicine, Heidelberg University, Heidelberg, Germany, ^5^Stem Cell Genetics, Institute of Frontier Life and Medical Sciences, Kyoto, Japan

**Background:** Microsatellite instability (MSI) is caused by deficient DNA mismatch repair (MMR) and occurs in more than 20 tumour types. MSI is frequent in colon, ovarian, endometrial and gastric cancers. Werner syndrome (WRN) helicase has an important role in maintenance of genome stability, DNA repair, replication and telomere maintenance.

**Method:** To identify new oncology targets, we performed CRISPR-Cas9 genome-wide screens in 324 human cancer cell lines. Integrating genomic data, biomarker analyses and target tractability, we prioritized over 600 candidate targets which are required for the fitness of cancer cells.

**Results:** WRN was selected as a promising new candidate synthetic-lethal target in MSI tumours from multiple cancer types. CRISPR and RNAi-based studies verified that WRN is selectively essential MSI cell lines and dispensable in MSS cancer cell lines. Moreover, we demonstrated that WRN is required to sustain in vivo growth of MSI colorectal cancer (CRC) cells. Mechanistically, the activity of WRN helicase domain is essential for MSI cell viability, and we demonstrated that WRN inhibition in MSI CRC cell lines induces double-stranded DNA breaks that cause massive genome instability, promoting apoptosis both in vitro and in vivo. Finally, we investigated the causal link between MMR-deficiency and WRN-dependency.

**Conclusion:** Collectively, our study establishes WRN as a synthetic lethal vulnerability in MSI cancers, representing an unexplored opportunity to develop a novel targeted therapy for MSI cancers.

**Disclosure:** Funded by Wellcome Trust Sanger Institute, Cambridge, UK

**Corresponding author:** Gabriele Picco

### 19. Uncovering the long tail of oesophageal cancer driver genes for patient stratification

#### Lorena Benedetti^1^, Thanos Mourikis^1^, Elisabeth Foxall^1^, Damjan Temelkovski^1^, Joel Nulsen^1^, Juliane Perner^2^, Matteo Cereda^3^, Christopher Yau^4^, Rebecca Fitzgerald^2^, Michael Howell^1^, Paola Scaffidi^1^, Francesca Ciccarelli^1^

##### ^1^The Francis Crick Institute, London, UK, ^2^University of Cambridge, Cambridge, UK, ^3^Italian Institute for Genomic Medicine, Torino, Italy, ^4^University of Birmingham, Birmingham, UK

**Background:** The incidence of oesophageal adenocarcinoma (OAC) in the UK is among the highest in the world and the five-year survival rate is below 20%. Its heterogeneous landscape, with high mutation and copy number burden where few cancer driver genes recur across patients, limits the choices of targeted therapies

**Method:** We developed a new method based on machine learning that, unlike most available methods, identifies altered cancer driver genes in each individual patient. Once cancer genes were identified in each patient, we clustered patients by recurrently perturbed biological processes to allow patient stratification. Finally, we experimentally validated representative genes in pre-cancer and cancer models.

**Results:** We applied our method to OACs from the Oesophageal Cancer Clinical and Molecular Stratification (OCCAMS) Consortium, a UK-wide initiative of tissue collection aiming at characterising OAC molecular landscape, stratifying patients and identifying new therapeutic targets. We identified cancer driver genes in each of the analysed patients, most of which are rare or patient-specific. However, they converge towards the perturbation of well-known biological processes related to cancer, including intracellular signalling, cell cycle regulation, proteasome activity and Toll-like receptor signalling. Interestingly, these genes are also altered in pre-cancer Barrett’s oesophagus, suggesting early clonal alteration. We mimicked the alteration of these genes in OAC lines and Barrett’s models, observing a growth promoting phenotype. Moreover, reverting their alteration led to cell death, pointing at dependencies that can be exploited in therapy.

**Conclusion:** Our machine learning approach allows the identification of cancer driver genes in individual patients independently of their alteration recurrence. This is particularly suitable for cancers with highly unstable mutational landscapes, like OAC, where recurrent alterations are rare. Our study proposes a new stratification of OAC patients and unravels vulnerabilities exploitable in therapy (Mourikis, et al. 2019 Nature communications, in press).

**Disclosure:** Cancer Research UK [C43634/A25487], London, UK, Cancer Research UK King’s Health Partners Centre at King’s College London, London, UK, Computational analyses were done using the High-Performance Computer at the National Institute for Health Research (NIHR) Biomedical Research Centre based at Guy's and St Thomas' NHS Foundation Trust and King's College London, London, UK. Funding for sample sequencing was through the Oesophageal Cancer Clinical and Molecular Stratification (OCCAMS) Consortium as part of the International Cancer Genome Consortium and was funded by a programme grant from Cancer Research UK (RG66287), London, UK.

**Corresponding author:** Lorena Benedetti

### 20. Investigating COX isoform dependency in intestinal tumourigenesis

#### Noha-Ehssan Mohamed^1^, Rachel Ridgway^1^, Owen Sansom^1^

##### ^1^Cancer Research UK Beatson Institute, Glasgow, UK

**Background:** Inflammation is known to play important roles in sporadic colorectal cancer as well as in colitis-associated cancer. Cyclooxygenase-2 (COX-2), the inducible COX isoform responsible for prostaglandin production, is highly expressed in colorectal adenomas and adenocarcinomas. Targeting COX-2 using non-selective (e.g. aspirin) as well as selective (e.g. celecoxib) COX-2 inhibitors reduces the incidence of colorectal carcinoma and prevents adenoma recurrence. Recently, it has been shown that COX-1 expression is elevated in response to COX-2 deletion in melanoma cell lines and in fibroblasts, resulting in production of prostaglandins. Whether a similar compensation happens in intestinal tumours is not known.

**Method:** To fully investigate COX isoform dependency, a genetically-engineered mouse (GEM) with a knockout of *Ptgs2* (gene coding for COX-2) was crossed to the small intestinal adenoma *Apc*^Min/+^ mouse, to investigate tumour initiation and progression. To investigate possible compensation by COX-1, another GEM where *Ptgs1* (gene coding for COX-1) is knocked into the *Ptgs2* locus, characterized by a full loss of COX-2 protein and overexpression of COX-1 protein (the COX-1^KI/KI^ mouse), was used. The COX-1^KI/KI^ mouse was crossed to the *Apc*^Min/+^ mouse.

**Results:** In the *Apc*^Min/+^ adenoma mouse, COX-2 knock out delayed tumour initiation, prolonged survival, and decreased intestinal tumour formation. Similarly, in the *Apc*^Min/+^ COX-1^K1/KI^ mouse, tumour initiation in the small intestine (SI) was delayed as reflected by a decrease in the total number of tumours developed as well as the size of the tumours in SI collected from 120 days old mice. Survival benefit was evident upon ageing *Apc*^Min/+^ COX-1^KI/KI^ mice, and this was associated with a decrease in SI tumour numbers. These tumours were characterized by being less proliferative and highly infiltrated by T-cells.

**Conclusion:** COX-2 is the isoform controlling intestinal tumour initiation and progression. COX-1 does not compensate for COX-2 in intestinal tumourigenesis.

**Disclosure:** Funded by Cancer Research UK Beatson Institute, Glasgow, UK

**Corresponding author:** Noha-Ehssan Mohamed

### 21. Genetically raised serum bilirubin levels and respiratory cancer: a cohort study using UK Biobank

#### Laura Horsfall^1^, Stephen Burgess^2^, Ian Hall^3^, Irwin Nazareth^1^

##### ^1^University College London, London, UK, ^2^MRC Biostatistics Unit, Cambridge, UK, ^3^University of Nottingham, Nottingham, UK

**Background:** Moderately raised serum bilirubin levels are associated with lower rates of respiratory cancer in large observational studies. It is unclear whether these relationships reflect antioxidant properties of bilirubin protecting against the carcinogenic effect of smoke oxidants or are due to confounding by lifestyle factors. We report the results of the first large-scale analysis of the causal relationship between bilirubin levels and respiratory cancer using a Mendelian randomisation approach.

**Method:** This research included unrelated participants of white British ancestry participating in the UK Biobank Resource. Average bilirubin levels in people homozygous for the minor T allele of rs887829 in the UDP-glucuronosyltransferase 1-1 gene are 8-10 µmol/l (80-100%) higher than those without this genotype. Using multivariable Poisson regression, we analysed the relationship between rs887829 T homozygosity and the incidence of respiratory cancers derived from national registers. The results were stratified by smoking behaviour with never smokers used as a negative control group.

**Results:** The analysis included 363,059 people, 1,962,584 person-years (PYs) at risk and 1188 respiratory cancer events. One in ten participants were homozygous for the T allele of rs887829 (n=35,881). There was no relationship between the genotype and respiratory cancer in never smokers or people smoking less than 20 cigarettes per day. The incidence rate in people homozygous for rs887829 T allele smoking 20 or more cigarettes per day was 10 per 10,000 PYs versus 17 per 10,000 PYs for the other genotypes. The adjusted incidence rate was 43% lower in rs887829 TT homozygotes relative to the other genotypes (incidence rate ratio: 0.57; 95%CI: 0.38 to 0.86; p=0.0065).

**Conclusion:** Moderately raised bilirubin may help protect people exposed to high levels of smoke oxidants against respiratory cancers. The role as a therapeutic target and a low-cost causal biomarker for disease risk stratification requires further research. Wellcome Trust funded:209207/Z/17/Z

**Disclosure:** Funded by Wellcome Trust, London, UK

**Corresponding author:** Laura Horsfall

### 22. Nr4a1-loss causes an acceleration of Myc-driven lymphomagenesis and an induction of gene of the B7-CD28

#### Alexander Deutsch^1^, Katrin Pansy^1^, Christine Beham-Schmid^1^, Andreas Prokesch^1^, Julia Feichtinger^1^

##### ^1^Medical University of Graz, Graz, Austria

**Background:** In aggressive lymphomas, low expression of *NR4A1* is associated with poor cancer-specific survival and its overexpression suppresses lymphoma cell growth indicating its tumor suppressor properties.

**Method:** To comprehensively study the function of *Nr4a1*-loss in lymphomagenesis, we intercrossed the *EµMyc* lymphoma-mouse with the *Nr4a1-/-* mouse. Furthermore, we transplanted lymphoma cells of *EµMyc*-*Nr4a1-/-* and *EµMyc*-*Nr4a1+/+* mice into immune-competent C57BL/6 mice and immune-deficient Fox-Chase-SCID beige mice. Finally, we determined the expression levels of the immune regulatory genes by RQ-PCR in our DLBCL patient cohort.

**Results:** Loss of *Nr4a1* in the *EµMyc* lymphoma model significantly accelerated lymphomagenesis. RNA-Seq data revealed upregulation of immune regulator genes of the B7-CD28 family in *EµMyc-Nr4a1*-/- lymphomas. Transplanting lymphoma cells with or without *Nr4a1*-loss into immune-competent mice led to accelerated lymphoma-development and a decreased survival in the absence of Nr4a1 and to no differences in immune-incompetent mice, indicating that the loss of *Nr4a1* results in a suppression of anti-lymphoma immune response. Gene expression analysis of primary and engrafted lymphomas revealed that *Nr4a1* is specifically implicated in the regulation of *Pd1-Pdl1-Pdl2* and *Ctla4-CD80-CD86* axis. Furthermore, flow cytometry analyses demonstrated that tumors transplanted from *EµMyc*-*Nr4a1-/-* mice exhibited a significantly higher percentage of infiltrating T cells. Interestingly, the infiltrating CD8+ T cells displayed higher expressed Pd1 on their surface in transplanted tumors derived from *EµMyc*-*Nr4a1-/-* mice, whereas Ctla-4 has not been investigated so far. In the human setting, we detected a significant negative association of NR4A1 expression levels and the *PD1- PDL1- PDL2-* and *CTLA4- CD80*-*CD86* in DLBCL confirming our mouse data.

**Conclusion:** Our data suggest that the tumor suppressive function of *Nr4a1* is mediated by the regulation of Pd1-Pdl1-Pdl2 and Ctla4-Cd80-Cd86 axis in aggressive lymphomas. Thereby, it seems that *Nr4a1* loss significantly contributes to the immune evasion of aggressive lymphomas and that it might act as a potential target for anti-lymphoma therapy.

**Disclosure:** Funded by MEFOgraz Vereinigung Forschungsförderung, Graz, Austria, Medical University of Graz, Graz, Austria

**Corresponding author:** Alexander Deutsch

## Prevention

### 23. Smoking cessation for cancer prevention: Can incentives play a role? Evidence from a Cochrane review

#### Caitlin Notley^1^, Sarah Gentry^2^, Jonathan Livingstone-Banks^3^, Linda Bauld^4^, Rafael Perera^3^, Jamie Hartman-Boyce^3^

##### ^1^Norwich Medical School, Norwich, UK, ^2^University of East Anglia, Norwich, UK ^3^Cochrane, University of Oxford, Oxford, UK, ^4^University of Edinburgh, Edinburgh, UK

**Background:** Smoking remains the leading preventable cause of cancer globally. Financial incentives are effective for smoking cessation, but long-term cessation is necessary for cancer prevention.

**Method:** Systematic review of randomised controlled trials, allocating individuals, workplaces, groups or communities to incentives or control. Including mixed populations and pregnant women. The outcome was abstinence from smoking at longest follow-up (at least six months from intervention start or to the end of pregnancy).

**Results:** 33 mixed-population studies met inclusion criteria, including 21,600 participants in community settings, clinics, workplaces, and drug clinics. The relative risk (RR) for quitting with incentives at longest follow-up compared with controls was 1.49 (95% confidence interval (CI) 1.28 to 1.73; 31 RCTs, adjusted N = 20,097; I^2^ = 33%). We conducted a sensitivity analysis to explore the effect of incentives offered up until long term follow-up compared to those where longest follow-up occurred after the incentive schedule had ended. Results were not sensitive to the exclusion of six studies where incentives were offered at long term follow-up (RR 1.40 95% CI 1.16 to 1.69; 25 RCTs; adjusted N = 17,058; I^2^ = 36%). We included 10 studies of 2,571 pregnant smokers. Together, nine of ten trials with usable data delivered a RR at longest follow-up (up to 24 weeks post-partum) of 2.38 (95% CI 1.54 to 3.69; 9 RCTs; N = 2273 participants; I^2^ = 41%), favouring incentives.

**Conclusion:** Overall there is high quality evidence that incentives improve smoking cessation at long term follow-up in mixed population studies and thus may have a role to play in cancer prevention. The effect of incentives appears to be sustained over time (both while in place and following discontinuation). There is moderate quality evidence that incentives for pregnant women improve smoking cessation rates, both at the end of pregnancy and post-partum.

**Corresponding author:** Caitlin Notley

### 24. Depressive symptoms and participation in breast and cervical cancer screening

#### Claire Niedzwiedz^1^, Kathryn Robb^1^, Srinivasa Vittal Katikireddi^1^, Jill Pell^1^, Daniel Smith^1^

##### ^1^University of Glasgow, Glasgow, UK

**Background:** Globally, more than 2 million women are diagnosed with breast or cervical cancer every year. Depressive symptoms have been implicated in cancer-related mortality, but the potential mechanisms through which these associations may operate are not well understood. Previous studies have investigated depression as a determinant of screening behaviour, but few have examined individual symptoms and accounted for personality factors. We aimed to assess how depressive symptoms (overall and individual symptoms) are associated with participation in breast and cervical cancer screening within the UK.

**Method:** 273 402 women in the UK Biobank cohort who were eligible for breast cancer screening (aged 50-70 years) and/or cervical cancer screening (<65 years) at baseline recruitment (2006-10) and those with follow-up data (2014-March 19) were identified. Depressive symptoms (4 items from Patient Heath Questionnaire) were self-reported at baseline. The primary outcomes were reporting being up to date with breast and cervical cancer screening. For prospective analyses, patterns of screening participation from baseline to follow-up were derived. Logistic regression was used to analyse associations, adjusted for potential confounders (including socio-demographic factors, disability, health behaviours, and neuroticism).

**Results:** More severe depressive symptoms (range 0-12) were associated with reduced screening for breast (OR=0.960, 95% CI: 0.950,0.970) and cervical cancer (OR=0.958, 95% CI: 0.950,0.966). Recent feelings of tiredness were consistently associated with decreased breast and cervical cancer screening participation. Prospective analyses revealed higher baseline depressive symptoms were related to decreased cervical cancer screening at follow-up (OR=0.955, 95% CI: 0.913,0.999; equivalent to a difference of 4.08% between the highest and lowest depressive symptom score), but not with breast cancer screening.

**Conclusion:** Depressive symptoms (especially tiredness and lethargy) were associated with reduced screening participation, particularly for cervical cancer. More severe depressive symptoms may act as a barrier for cancer screening participation and could be an indicator for more proactive strategies to improve uptake.

**Disclosure:** Funded by Medical Research Council, Swindon, UK

**Corresponding author:** Claire Niedzwiedz

## Early detection, diagnosis and prognosis

### 25. Renal cell carcinoma patients under 60 should be screened for colorectal carcinoma

#### Joseph John^1^, Eleanor Burden^2^, Edward Smyth^1^, Adam Chambers^1^

##### ^1^Taunton and Somerset NHS Foundation Trust, Taunton, UK, ^2^Royal Devon and Exeter Hospital, Exeter, UK

**Background:** The link between renal cell carcinoma (RCC) and colorectal cancer (CRC) is widely recognised but poorly quantified. This systematic review describes the current available evidence and considers whether we should take additional steps to screen for CRC in patients diagnosed with RCC.

**Method:** Literature searches were performed using Pubmed and Web of science. Six hundred and twelve papers were returned, of which papers were selected which quantified the risk of antecedent, synchronous or subsequent CRC in RCC patients.

**Results:** Seven studies met our inclusion criteria. The inclusion criteria used in each study were heterogenous. Three papers were single centre studies assessing their institutional records, giving risks of CRC in the presence of RCC between 3.67 and 4.70%. Four were national or regional population-based studies from the USA (3) and Norway (1). Standardised incidence ratios (SIR) were calculated in three of these. The largest study (n = 194,329 urological cancer patients) identified a modestly elevated SIR of CRC in patients with RCC (1.14, 95% confidence interval 1.04 - 1.25), with an increasing SIR with decreasing age of RCC diagnosis. Of the further two studies reporting SIRs, one indicated an increased SIR of 3.1 - 3.4 (p < 0.05, n = 551 RCCs), and one reported no significantly different SIR of colon cancer in RCC patients, but excluded rectal cancer from analysis.

**Conclusion:** The highest quality available evidence suggests an association between RCC and CRC, with a possible stronger association in younger patients. The advent of highly sensitive and specific quantitative faecal immunochemical testing (QFIT) means we should consider non-invasive screening for CRC in RCC patients who fall below the current age for national bowel cancer screening. Such screening would constitute effective resource allocation.

**Disclosure:** Funded by Taunton and Somerset NHS Foundation Trust, Taunton, UK

**Corresponding author:** Joseph John

### 26. SWATH mass spectrometry: Quantitative mapping of soft tissue sarcomas by digital proteome profiling

#### Lukas Krasny^1^, Martina Milighetti^1^, Alexander T.J. Lee^1^, Aik-Choon Tan^2^, Robin Jones^3^, Paul H. Huang^1^

##### ^1^The Institute of Cancer Research, London, UK, ^2^University of Colorado, Aurora, US, ^3^Royal Marsden Hospital, London, UK

**Background:** Soft tissue sarcomas (STS) are a rare and heterogeneous group of cancers. Our knowledge of STS biology is limited and although progress has been made in the genetic characterization of these diseases, comprehensive molecular profiling at the protein level has not been undertaken. One of the challenges associated with tumour proteomics is the presence of formalin-induced protein crosslinks in formalin-fixed paraffin-embedded (FFPE) tissue which complicates conventional proteomic workflows.

**Method:** In this study, we used digital proteome profiling by sequential window acquisition of all theoretical fragment ion spectra (SWATH) mass spectrometry (MS) to characterise a discovery cohort of STS cases (n=39) across 4 histological subtypes. Validation of findings was performed in an independent cohort of STS specimens (n=64) using a conventional proteomic approach that involves TMT labelling and off-line fractionation.

**Results:** Using SWATH-MS, we quantified 2865 proteins in the discovery cohort from starting material equivalent to a 5μm FFPE tissue section. Significantly altered expression levels of 884 proteins were found by ANOVA (FDR<0.05) in at least one of the four STS subtypes. Further analysis of these proteins by principal component analysis (PCA) and hierarchical clustering (HC) revealed distinct separation of samples into three subgroups corresponding to STS histological subtypes. Gene set enrichment analysis (GSEA) was employed to identify underlying biological processes and candidate therapeutics that are enriched in individual subgroups. In the validation cohort, 5642 proteins were quantified with 2114 proteins showing significantly altered expression levels in at least one of the STS subtypes (ANOVA, FDR<0.05) and analysis by PCA and HC showed similar histological subtype separation and biological pathways as the discovery cohort.

**Conclusion:** We demonstrate the potential of SWATH-MS for the molecular characterization of STS with minimal consumption of readily available FFPE tissue material. To our knowledge, this study is the most comprehensive analysis of the STS proteome to date.

**Disclosure:** Funded by Biomedical Research Centre at The Royal Marsden NHS Foundation Trust, London, UK, The Institute of Cancer Research, London, UK, The Pathological Society, London, UK, The Royal Marsden Cancer Charity Sarcoma Research Fund, London, UK, Breast Cancer Now, London, UK

**Corresponding author:** Lukas Krasny

### 27. Integrating Germline and Somatic Genetic Test Result Data into th English National Cancer Registration and Analysis Service

#### Fiona McRonald^1^, Brian Shand^1^, Sophia Richardson^1^, Katrina Lavelle^1^, Sally Vernon^1^, Caroline Brook^1^, Francesco Santaniello^1^, Joanna Pethick^1^, Margreet Luchtenborg^1^, Steven Hardy^1^, Jem Rashbass^1^

##### ^1^National Disease Registration, Public Health England, London, UK

**Background:** The National Cancer Registration and Analysis Service, run by Public Health England, collects, collates and quality assures data on every cancer diagnosed or treated within English NHS hospitals. We are now linking this population-level clinical data to germline and somatic genetic test results collected from NHS laboratories.

**Method:** We have established regular, high quality, somatic test result feeds from eleven NHS genetics and pathology laboratories, covering approximately 80% of solid tumour molecular diagnostic testing within England. We also have germline data submissions from ten genetics laboratories; we have developed a novel method to pseudonymise germline demographics upon upload, thus protecting the identity of healthy people undergoing predictive genetic testing. We have developed the NCRAS registration system to capture source laboratory data in diverse formats; records are standardised by a combination of computational methods and manual registration by a core group of staff specially trained in interpreting molecular test results.

**Results:** Our pilot work covering 12 months’ worth of somatic testing data has recorded and linked >60,000 genetic test results on >26,000 tumours, covering >1,100 distinct combinations of gene and tumour site. In addition, we have collated data on germline tests on the *BRCA1* and *BRCA2* genes and released summary variant counts on >22,000 individuals back to the NHS clinical genetics community. The released aggregate data are used to calculate variant frequencies and assist in national consensus reclassification of BRCA1/2 variants of uncertain clinical significance.

**Conclusion:** This work will enable audit of the scope, availability and usage of molecular diagnostics within NHS cancer pathways and services, and will support epidemiological research and clinical trials. Patient-level and tumour-level linkage of molecular result data embedded within NCRAS is already changing clinical management for high risk BRCA1/2 families, and future work will allow new discoveries about associations between individual DNA aberrations, treatments, and overall outcomes and survival.

**Disclosure:** Funded by Public Health England, London, UK

**Corresponding author:** Steven Hardy

### 28. Survival outcomes in oropharyngeal cancer: a decision tree analysis

#### Francesca De Felice^1,2^, Laia Humbert-Vidan^2^, Mary Lei^2^, Andrew King^2^, Teresa Guerrero Urbano^2^

##### ^1^Sapienza University of Rome, Rome, Italy, ^2^Guy’s and St Thomas’ NHS Foundation Trust

**Background:** Our aim was to evaluate overall survival (OS) using decision tree algorithms in oropharygeal cancer patients.

**Method:** In total 273 patients with newly diagnosed oropharyngeal cancer were identified from March 2010 to December 2016. All patients were treated with definitive intensity-modulated radiotherapy (IMRT). The open-source R software was used. OS was estimated by Kaplan-Meier method. Nine predictor variables, including gender, age, primary tumor site, alcohol, tobacco smoking, HPV status, clinical T classification, clinical N classification and early responders, were investigated. Important explanatory variables were selected using the random forest approach. A classification tree that optimally partitioned patients with different OS rates was then built.

**Results:** The 5-year OS for the entire population was 78.1%. The main important variables were HPV status, N stage and early complete response to treatment. Patients were partitioned in five groups: i) patients with HPV-related oropharyngeal cancer (12% probability of death); ii) patients who had HPV-negative disease without nodal involvement at diagnosis (32% probability of death); iii) patients who had HPV-negative cancer, N stage < 2c and were early responders (36% death probability); iv) patients who had HPV-negative cancer, N stage ≥ 2c, and were early responders (83% death probability); v) patients who had HPV-negative cancer, with nodal involvement at diagnosis and were not early responders (71% death probability).

**Conclusion:** This classification tree could help to guide future research in oropharyngeal cancer field. Further analysis on a validation cohort is required to confirm our results.

**Corresponding author:** Francesca De Felice

### 29. Assessment of tissue composition with digital pathology in colorectal cancer

#### Enric Domingo^1^, Aikaterini Chatzipli^2^, Susan Richman^3^, Andrew Blake^1^, Claire Hardy^2^, Celina Whalley^4^, Keara Redmond^5^, Ian Tomlinson^4^, Philip Dunne^5^, Steven Walker^6^, Andrew Beggs^4^, Ultan McDermott^2^, Graeme Murray^7^, Leslie Samuel^8^, Matt Seymour^3^, Philip Quirke^3^, Tim Maughan^1^, Viktor Koelzer^9^

##### ^1^University of Oxford, Oxford, UK, ^2^Wellcome Trust Sanger Institute, Cambridge, UK, ^3^Leeds Institute of Cancer and Pathology, Leeds, UK, ^4^University of Birmingham, Birmingham, UK, ^5^Queen’s University Belfast, Belfast, UK, ^6^Almac Diagnostics, Craigavon, UK, ^7^University of Aberdeen, Aberdeen, UK, ^8^Aberdeen Royal Infirmary, Aberdeen, UK, ^9^University of Zurich, Zurich, Switzerland

**Background:** The tumour microenvironment is a key feature to understand cancer biology. Quantification of tissue composition is usually based on visual pathological review (VPR) or deconvolution of whole genome molecular data. The former is a direct measurement with modest reproducibility while the latter is an indirect measurement of unclear accuracy and is expensive. Here we test digital pathology coupled with machine learning as a new tool to assess tissue composition.

**Method:** As part of the Stratification in COloRecTal cancer (S:CORT) programme, over 500 colorectal cancer (CRC) paraffin blocks from resections and biopsies were sequentially sectioned for RNA/DNA extractions and two Haematoxylin and Eosin stained (H&E) sections. RNA expression microarrays, targeted DNA sequencing and DNA methylation arrays were applied. Tissue composition was obtained by a deep neural net (DNN) algorithm after supervised training on >1,500 tissue areas. Tumour purity estimates (TPE) were obtained from VPR and RNA/methylation arrays. Copy number alterations were adjusted using different TPE and compared. Similar analyses were performed with TCGA CRCs.

**Results:** DNN estimates including area and cell counts were obtained for tumour, desmoplastic stroma, inflamed stroma, mucin/hypocellular stroma, muscle, necrosis and white space. DNN estimates on the same H&Es obtained matching results (r=1.0). Comparison of paired H&Es showed very high correlations (r~0.85). TPE by VPR consistently underestimated purity which resulted in ~10% overestimation of copy number calls. Conversely, TPE from either RNA or methylation deconvolution showed consistent overestimation resulting in ~10% of copy number undercalls.

**Conclusion:** Tissue composition analysis with DNN allows analytical robustness, automatization and standardization and provides very high reproducibility at single cell resolution. DNN-based TPE are more accurate than VPR or deconvolution from genome-wide omic platforms which tend to under and overestimate tumour purity respectively. DNN could be used to better plan and assess downstream molecular analyses and investigate tissue-based metrics as potential biomarkers in clinical trials.

**Disclosure:** Funded by Medical Research Council, Cancer Research UK, London, UK

**Corresponding author:** Enric Domingo

### 30. Obesity, body image and past screening experiences: impacts on breast screening participation

#### Kate McBride^1^, Sam Hogan^1^, Freya MacMillan^1^

##### ^1^Western Sydney University, Parramatta, Australia

**Background:** Obesity is an urgent global health issue which increases risk of chronic disease, such as breast cancer. Evidence suggests individuals with a higher body mass index (BMI) are less likely to engage in preventative health screens, such as breast screening programs, due to self-stigmatisation and a prior poor experiences, despite obesity being the number one risk factor for breast cancer.

**Method:** An online survey, investigating breast screening participation, body shame and past screening experiences was distributed to women across Australia via targeted social media marketing. Descriptive statistics, chi-square tests of independence and generalised linear regression were used to analyse the data.

**Results:** Among the convenience sample of women who completed the survey (n=892), negative past screening experiences were correlated with reduced levels of rescreening (p = 0.0001). Higher BMI was also associated with reduced rescreening (p = 0.007) compared to those with a lower BMI. Increased body shame scores were also linked to negative previous screening experiences (p <0.0001). These data also suggest body image disturbances may be correlated with BMI. Low body shame was associated with higher healthcare seeking scores (p <0.0001).

**Conclusion:** Body image, obesity and past screening experiences influence how women access breast screening programs. Having a higher BMI appears to contribute to negative screening experiences via increased body image shame, both of which may reduce screening, and in particular rescreening among obese women. Consideration of these issues is warranted if screening participation is to be optimised in this group of higher risk women, as well as education for mammographic staff on sensitive handling of obese women.

**Disclosure:** Funded by Western Sydney University, Parramatta, Australia

**Corresponding author:** Kate McBride

### 31. Occupational exposure and smoking adjusted risk of bladder cancer. Population based cohort studies in the Nordic countries

#### Kishor Hadkhale^1^, Jan Ivar Martinsen^2^, Elisabete Weiderpass^3^, Kristina Kjaerheim^2^, Par Sparen^4^, Laufey Tryggvadottir^5^, Elsebeth Lynge^6^, Eero Pukkala^7^, Tom K Grimsrud^2^

##### ^1^Tampere University, Tampere, Finland, ^2^Cancer registry of Norway, Oslo, Norway, ^3^International Agency for Research on Cancer, Lyon, France, ^4^Karolinska Institute, Stockholm, Sweden, ^5^Icelandic cancer registry, Reykjavik, Iceland, ^6^University of Copenhagen, Copenhagen, Denmark, ^7^Finnish cancer registry, Helsinki, Finland

**Corresponding author:** Kishor Hadkhale

Portions of this abstract published previously, See https://trepo.tuni.fi/bitstream/handle/10024/104597/978-952-03-0896-4.pdf?isAllowed=y&sequence=1 and see p. 17 here https://www.ancr.nu/dyn/resources/File/file/5/7085/1518700687/virrat_2018_program_after_the_weekend_final.pdf

### 32. Non-invasive methylation test to detect cervical pre-cancer in self-collected vaginal and urine specimens

#### Belinda Nedjai^1^, Caroline Reuter^1^, Hollingworth Toni^1^, Janet Austin^1^, Louise Cadman^1^, Jack Cuzick^1^, Attila Lorincz^1^

##### ^1^Queen Mary University of London, London, UK

**Background:** The implementation of HPV testing as a primary screen will soon become the norm worldwide. Because HPV testing is a very sensitive method, but not specific enough, the choice of an appropriate triage strategy for hrHPV positive women will be one of the future key issues facing the cervical screening community. Clinician taken samples are the gold standard but self-sampling including urine may be a useful alternative. We have developed a triage classifier for the detection of CIN2+, based on DNA methylation of HPV16, HPV18, HPV31 and HPV33 and the human gene *EPB41L3*. We will test S5 classifier on two non-invasive specimens: a self-collected vaginal sample and urine. We aim to assess whether S5 can identify women who are CIN2+ using self-collected samples and comparing several collection devices.

**Method:** Women attending the colposcopy clinic at The Royal London Hospital as a consequence of abnormal screening cytology and/or a positive HPV result were recruited as part of the ‘Self-sampling for vaginal HPV. 503 women provided a urine sample using the Colli-Pee™ device. In total 600 women provided self-collected vaginal samples using either Flocked swab and Diagene or HerSwab and Qvintip. DNA was extracted, Bisulfite converted, followed by pyrosequencing assays for the 6 S5 markers. Average methylation was calculated to generate the S5 score.

**Results:** S5 showed a good and statistically significant separation between <CIN2 and CIN2+ samples for both urine and vagina self-samples (p=<0.0001). The area under the ROC curve was 0.7254 (p=<0.0001) for urine samples and 0.7388 (p=<0.0001) for vaginal self-samples. At the pre-defined cut-off of 0.8, the sensitivity for urine samples was 66% and specificity 72% and vaginal self-samples was 71% and specificity 68%.

**Conclusion:** We demonstrated that S5 can be successfully amplified in urine and vaginal self-collected samples and that the classifier is able to correctly identify CIN2+ women.

**Disclosure:** Funded by Cancer Research UK, London, UK

**Corresponding author:** Belinda Nedjai

### 33. Non-Attendance in Two-week wait and Urgent Colorectal Cancer Referrals

#### Vincenza Scanella^1^, Harpreet Sekhon Inderjit Singh^2^, Rabiya Aseem^2^, Jason Smith^2^, Nikhil Pawa^2^

##### ^1^Imperial College London, London, UK, ^2^West Middlesex University Hospital, Isleworth, UK

**Background:** Colorectal cancer (CRC) is the second commonest cause of cancer death in the UK, attributing to 10% of cancer mortality. Survival is dependent upon early detection. The Two-week-wait (2WW) referral pathway was implemented in the UK in July 2000 to reduce delays in diagnosis and decrease mortality associated with CRC. Non-attendance of these appointments has adverse clinical outcomes for patients. The reasons for non-attendance are not clearly understood with no quantitative research previously performed. This study aims to determine the association between Age, Sex, Ethnicity, Geographical location (postcode) and Socioeconomic status (SES) on non-attendance in 2WW and urgent CRC referrals.

**Method:** A retrospective analysis of a prospectively collated database of 2WW and urgent CRC referrals at our unit from January 2016 to December 2018 was performed. Variables regarding patient age, sex, attendance status, ethnicity (Census 2011), postcode, and socioeconomic status were recorded. Chi-squared, Spearman’s Rank and multivariate analyses were performed to determine the relationship between these variables and non-attendance. The White-British population was used as the control group.

**Results:** A total of 9,829 patients (49.45% male, 50.55% female, mean age 64.42 years) were analysed with an overall non-attendance rate of 12.31% (1,210/9,829). There was an increased non-attendance risk in 1) Younger populations (Age <55 years: OR=1.189 CI=1.049-1.349 p=0.0076); 2) Three ethnic cohorts: Asian Other (OR=0.6778 CI=0.5151-0.892 p=0.0067), Black Other (OR=0.3852 CI=0.2046-0.7255 p=0.0045) and Not Stated (OR= 0.8102 CI=0.2162-0.3321 p=0.011); 3) Six postcodes (p <0.05) of which non-attendees in TW3 also had a significant risk of social deprivation. There was no correlation between gender or SES and non-attendance. Multivariate analysis confirmed the association between age and non-attendance (p=0.0012).

**Conclusion:** This novel study has identified high-risk groups for non-attendance. Further qualitative research into high-risk groups needs to be performed to allow early detection and diagnosis of CRC and improve clinical and oncological outcomes.

**Corresponding author:** Harpreet Sekhon Inderjit Singh

### 34. Clinical utility of circulating microRNAs in malignant germ cell tumours

#### Matthew Murray^1^, Nicholas Coleman^1^

##### ^1^University of Cambridge, Cambridge, UK

**Background:** The protein biomarkers AFP/HCG have limited sensitivity/specificity for diagnosing malignant germ-cell-tumours (GCTs). We previously showed that microRNAs from the miR-371-373 and miR-302/367 clusters are universally overexpressed in all malignant GCT tissues, regardless of patient age, tumour site or histological subtype, but are not co-ordinately over-expressed in any other cancer type or disease state. Here we provide an overview of our research studying these microRNAs as GCT biomarkers and consider prospects for future clinical applications.

**Method:** We have developed a highly sensitive pre-amplified qRT-PCR technique for robust detection of microRNAs from the miR-371-373 and miR-302/367 clusters in biospecimens. Our method has been adopted by multiple other groups internationally and validated in over 2,000 patients. Our pipeline includes quality control checks, use of an exogenous spike-in control and normalisation against the endogenous microRNA miR-30b-5p, prior to data analysis. Cost analysis and patient/public involvement initiatives regarding acceptability of circulating microRNA testing are underway.

**Results:** Results from our group and others show that a four-serum miRNA panel (miR-371a-3p, miR-372-3p, miR-373-3p and miR-367-3p) has high sensitivity/specificity for diagnosing malignant GCTs. Individually, miR-371a-3p shows the most utility as it is most dynamic and most accurately reflects disease activity. Levels of these microRNAs are useful for disease-monitoring and early detection of relapse.

**Conclusion:** Circulating microRNAs should improve future clinical management of patients with malignant GCTs. Potential benefits include: (i) reducing CT scans in follow-up, lowering cumulative radiation exposure to patients and offering cost savings to healthcare systems; (ii) identifying patients with apparent clinical stage I (CSI) seminoma who have persistently elevated serum microRNA levels post-orchidectomy, suggesting micrometastatic disease. Such patients may benefit from adjuvant chemotherapy to prevent subsequent recurrence. This approach will also prevent overtreatment of the majority of CSI patients who are not destined to relapse. Importantly, circulating microRNA testing appears highly acceptable to patients.

**Disclosure:** Funded by St. Baldrick's Foundation, Monrovia, US, Isaac Newton Trust, Cambridge, UK

**Corresponding author:** Matthew Murray

### 35. Interim results from the IMPACT study: evidence for PSA screening in *BRCA2* mutation carriers

#### Elizabeth Page^1^, Elizabeth Bancroft^2^, Mark Brook^1^, Melissa Assel^3^, Judith Offman^4^, Zsofia Kote-Jarai^1^, Andrew Vickers^3^, Hans Lilja^3^, Ros Eeles^1^

##### ^1^The Institute of Cancer Research, London, UK, ^2^Royal Marsden NHS Foundation Trust, London, UK, ^3^Memorial Sloan Kettering Cancer Center, New York, US, ^4^King’s College London, London, UK

**Background:** Mutations in *BRCA2* cause a higher risk of early-onset aggressive prostate cancer (PrCa). The IMPACT study is evaluating targeted PrCa screening using PSA in men with germline *BRCA1/2* mutations. The objective was to report the utility of PSA screening, PrCa incidence, positive predictive value of PSA, biopsy and tumour characteristics after three years’ screening, by BRCA status.

**Method:** Men aged 40–69 years with germline pathogenic *BRCA1/2* mutation and male controls testing negative for a familial *BRCA1/2* mutation, were recruited. Participants underwent PSA screening for three years, and if PSA >3.0ng/ml, men were offered prostate biopsy. Statistical analyses included Poisson regression offset by person-years follow-up, chi-squared tests for proportions, t-tests for means, and univariate logistic regression was applied to PSA predictors.

**Results:** 3,027 subjects (2932 unique individuals) were recruited (919 *BRCA1* carriers, 709 *BRCA1* non-carriers; 902 *BRCA2* carriers; 497 *BRCA2* non-carriers). After 3 years screening 527 men had PSA >3.0ng/ml, 357 biopsies performed, and 112 PrCas diagnosed (31 *BRCA1* carriers, 19 *BRCA1* controls; 47 *BRCA2* carriers, 15 *BRCA2* controls). A higher compliance with biopsy was observed in *BRCA2* carriers compared with controls (82% vs 66%). Cancer incidence rate per 1,000 person years was higher in *BRCA2* carriers than non-carriers (19.4 vs 12.0;p=0.03); *BRCA2* carriers were diagnosed younger (61 vs 64years;p=0.04) and were more likely to have clinically-significant disease than *BRCA2* non-carriers (73% vs 40%;p=0.03). No differences in age or tumour characteristics were detected between *BRCA1* carriers and controls. The 4 kallikrein-marker model discriminated better (AUC=0.73) for clinically-significant cancer at biopsy than PSA alone(AUC=0.65).

**Conclusion:** After three years’ screening, compared with non-carriers, *BRCA2* mutation carriers were associated with higher incidence of PrCa, younger age of diagnosis and clinically-significant tumours. Therefore, systematic PSA screening is indicated for men in this age group. Further follow-up is required to assess the role of screening in *BRCA1* mutation carriers.

**Disclosure:** Funded by Cancer Research UK, London, UK, The Ronald and Rita McAulay Foundation, Hamilton, Bermuda, NIHR BRC at The Institute of Cancer Research, London, UK, The Royal Marsden NHS Foundation Trust, London, UK

**Corresponding author:** Ros Eeles

### 36. Development and validation of a risk prediction model for lung cancer with common health examination indexes

#### Zhangyan Lyu^1,2,3,4^, Ni Li^1,2,3,4^, Fengwei Tan^1,2,3,4^, Jiang Li^1,2,3,4^, Chunqing Lin^1, 2,3,4^, Hongda Chen^1, 2,3,4^, Jiansong Ren^1,2,3,4^, Jufang Shi^1,2,3,4^, Kai Su^1,2,3,4^, Fang Li^1,2,3,4^, Xiaoshuang Feng^1,2,3,4^, Luopei Wei^1,2,3,4^, Xin Li^1,2,3,4^, Yan Wen^1,2,3,4^, Gang Wang^5^, Shuohua Chen^5^, Shouling Wu^5^, Min Dai^1,2,3,4^, Jie He^1,2,3,4^

##### ^1^National Cancer Center, Beijing, China, ^2^National Clinical Research Center for Cancer, Beijing, China, ^3^Cancer Hospital, Beijing, China, ^4^Chinese Academy of Medical Sciences and Peking Union Medical College, Beijing, China, ^5^Kailuan General Hospital, Beijing, China

**Background:** Lung cancer has been the most common cancer and leading cause of cancer-related death for several decades worldwide, especially in China, the most populous country. Low-dose computed tomography (LDCT) has been proven to reduce lung cancer mortality. A user-friendly lung cancer risk prediction model could help standardize the selection of high-risk population for LDCT screening and alter individuals’ lifestyle factors to lower their risk. We thus sought to develop and internally validate a simple model for lung cancer based on a prospective cohort study in China.

**Method:** A total of 138,150 people was prospectively observed from 2006 to 2015 for lung cancer incidence. Stepwise multivariable-adjusted logistic regressions with *P*_entry_=0.15 and *P*_stay_=0.20 were conducted to select the candidate variables included in the prediction model. Concordance statistics (C-statistics) and Hosmer–Lemeshow tests were used to evaluate discrimination and calibration, respectively. Ten-fold cross-validation was used for internal validation.

**Results:** During a median of 9-year follow-up, a total of 1088 (0.79 %) lung cancer cases were identified. The simple model including age and smoking generated a C-statistics of 0.71. The full model additionally included sex, alcohol consumption, body mass index (BMI), low-density lipoprotein cholesterol (LDL-C), and C-reactive protein (CRP) showed significantly better predictive performance regarding discrimination (C-statistics=0.73, *P*<0.01). In 10-fold cross-validation, the average C-statistic across the 10 test sets was similar (0.73). Model calibrated well across deciles of predicted risk (*P*_HL_=0.48). The predicted risk of lung cancer in the top decile was 0.04% vs. 2.36% in the bottom decile (Odds ratio [OR]=98.16).

**Conclusion:** We developed and internally validated an easy-to-use risk prediction model for lung cancer among the Chinese population that could provide guidance for LDCT screening and early detection of lung cancer.

**Corresponding author:** Ni Li, Min Dai, and Jie He

### 37. Hospital activity before and after diagnosis of monoclonal gammopathy of undetermined significance (MGUS)

#### Maxine Lamb^1^, Eleanor Kane^1^, Alexandra Smith^1^, Eve Roman^1^

##### ^1^University of York, York, UK

**Background:** Monoclonal gammopathy of undetermined significance (MGUS; ICD-O-3=9761/1) is a premalignant clonal disorder that progresses to myeloma at ~1% per year. Whilst MGUS is often diagnosed incidentally and other symptoms of myeloma are absent, several follow-up studies have reported relationships with co-morbidities and poor survival. Illness patterns before MGUS diagnoses have, however, not been examined in relation to those seen after diagnosis. Here we compare hospital activity of people with MGUS and patients with mature B-cell malignancies to that in the general population.

**Method:** The study is set within the UK’s Haematological Malignancy Research Network (HMRN, www.hmrn.org), which contains two established population-based cohorts: a patient cohort of haematological malignancies (cases), and a control cohort comprising 10 age-, sex- and area-matched individuals for cases diagnosed 01/01/2009-31/12/2015. HMRN operates with Section 251 support, and both cohorts are linked to national Hospital Episode Statistics. Inpatient and outpatient visits from five years before to three years after diagnosis were counted, excluding haematology.

**Results:** Over the study period, cases with MGUS (n=2,239) had significantly higher hospital activity rates compared to controls (n=22,390). Before diagnosis, monthly attendance rates per 100 persons averaged 30.6 (95%CI:30.3-30.9) among cases and 20.9 (20.9-21.0) in controls, with activity rates in the controls remaining constant over time. The difference was driven by outpatient attendances and activity in cases remained high after diagnosis. Outpatient specialties with high activity before diagnosis (including rheumatology, orthopaedics, dermatology and nephrology) were similar to those found after. This unusual pattern of activity was not seen in any other haematological malignancies or precursor conditions.

**Conclusion:** MGUS patients have increased hospital activity unrelated to haematology several years before diagnosis, and the pattern is sustained after diagnosis. The underpinning specialities we observed are consistent with the post-diagnostic literature. That the pattern is evident at least five years before diagnosis impacts on causal and pathogenic hypotheses.

**Disclosure:** Funded by Bloodwise, London, UK, Cancer Research UK, London, UK

**Corresponding author:** Maxine Lamb

### 38. Methylation Sensitive High Resolution Melting (MS-HRM) Assay for the Detection of *BRCA1* and *BRCA2* Promoter Hypermethylation

#### Diana Pelka^1^, Jack Grant^1^, Sasha Hansel^1^, David Moore^1^, Phil Bennett^1^, Geraldine Thomas^2^, Gareth Gerrard^1^

##### ^1^Sarah Cannon Molecular Diagnostics, London, UK, ^2^Imperial College London, London, UK

**Background:** *BRCA1* & *BRCA2* genes encode key components of the DNA double-strand break repair pathway. Cancers driven by loss of *BRCA1/*2 are associated with sensitivity to PARP inhibitors (PARPi), such as olaparib, through the synthetic-lethality of concomitantly blockading the single-strand repair pathways mediated by PARP. Monoallelic *BRCA1/2* mutations require a ‘second-hit’ to the unaffected allele, since only tumours with complete abrogation of *BRCA1/2* are targetable with PARPi. One recognised second-hit mechanism is gene promoter hypermethylation, which can also cause gene silencing in the absence of mutations. We sought to use extant MS-HRM protocols (based on a validated *MLH1* assay) using primer and control kits from MethylDetect ApS, Denmark, to implement a *BRCA1*/2 promoter hypermethylation assay.

**Method:** 14 triple-negative, grade 3 invasive ductal breast carcinoma (BC) and 13 prostate adenocarcinoma (PA) FFPE samples were sourced from the Imperial College Healthcare Tissue Bank. They were macro-dissected to obtain DNA from paired tumour and normal tissue. 100ng DNA from each was bisulphite-treated (EpiTect Fast, Qiagen) in a 20μL reaction and 3μL used in the MS-HRM reaction, along with CpG-flanking primers for either *BRCA1* or *BRCA2*. Kit provided methylation controls were used for IQC. The MS-HRM reactions were run on a Qiagen RotorGeneQ, using EpiTect HRM mastermix (Qiagen) and analysed with the RotorGene v2.3.1.49 software.

**Results:** 7/13 (53.8%) and 0/12 BC samples showed *BRCA1* and *BRCA2* promoter hypermethylation, respectively; none of the 13 PA samples were hypermethylated for either gene. 1 *BRCA1* and 2 *BRCA2* samples failed to yield usable results (both BC).

**Conclusion:** The detection of *BRCA1* hypermethylation in over half of the BC samples in this limited-scale implementation of a low-cost, rapid and sensitive assay, demonstrates the potential utility of this approach for stratifying patients for PARPi therapy.

**Corresponding author:** Gareth Gerrard

### 39. Explaining socio-economic differences in bladder cancer survival

#### Beth Russell^1^, Mieke Van Hemelrijck^1^, Truls Gårdmark^2^, Lars Holmberg^1^, Pardeep Kumar^3^, Andrea Bellavia^4^, Christel Häggström^5^

##### ^1^King's College London, London, UK, ^2^Karolinska Institute, Stockholm, Sweden, ^3^Royal Marsden NHS Foundation Trust, London, UK, ^4^Havard T.H Chan School of Public Health, Boston, US, ^5^Uppsala University, Uppsala, Sweden

**Background:** There is increasing evidence that socioeconomic status (SES) may influence the survival of bladder cancer (BC) patients. However, the underlying mechanisms behind the proposed association are yet to be elucidated. Therefore, this novel study aims to disentangle the heterogeneity in the survival outcomes of different SES groups by identifying any potential mediators of the relationship.

**Method:** The Bladder Cancer Database Sweden (BladderBaSe) was used to select patients diagnosed between 1997 and 2014 with Tis/Ta-T4 disease. Education level was used as a proxy for SES. Accelerated failure time models were used to investigate the association between SES and survival. Mediation analysis, using the four-way decomposition method, was then conducted to assess the role of several potential mediators. The mediation analysis was then stratified by non-muscle invasive bladder cancer (NMIBC) and muscle invasive bladder cancer (MIBC) patients. All analyses were fully adjusted.

**Results:** 37,755 patients were identified from BladderBaSe (74% NMIBC and 26% MIBC). Patients diagnosed with NMIBC and MIBC who had a medium/high SES were found to have a significantly increased overall and BC-specific survival when compared to those with a low SES. Optimal treatment was found to be a weak mediator in non-metastatic MIBC patients (2%). Age was found to mediate the relationship by 31%, and hospital type by 4% in the NMIBC patients only. The time from referral to transurethral resection of the bladder tumour (TURBT) was a considerable mediator (14%) in the MIBC patients only.

**Conclusion:** Mediation analysis suggested that the hypothesised relationship between SES and survival was contributed to by several factors with some being avoidable e.g. a delay in time between referral and TURBT, and patients receiving optimal treatment. Other factors such as age and hospital type are less manageable nevertheless highlight the importance of standardization of clinical care across SES groups.

**Disclosure:** Funded by King's College London, London, UK

**Corresponding author:** Mieke Van Hemelrijck

### 40. An Analysis of Prognostic Biomarkers of the Systemic Inflammatory Response in Cancers of Unknown Primary

#### Mark Stares^1^, Rebekah Patton^1^, Gillian Knowles^2^, Rachel Haigh^2^, Lucy Dobbs^2^, Barry Laird^2^, Colin Barrie^2^, Sally Clive^2^

##### ^1^NHS Lothian, Edinburgh, UK, ^2^University of Edinburgh, Edinburgh, UK

**Background:** Cancers of unknown primary (CUP) represent between 3-5% of all malignancies worldwide. In favourable clinico-pathological subsets median survival is 24 months but can be as little as 1-3 months in ‘poor prognosis’ patients. To rationalise investigations and treatment appropriately it is essential to provide accurate prognostic data. Biomarkers of the systemic inflammatory response (albumin and CRP combined in the modified Glasgow prognostic score (mGPS)) predict survival in cancers with an established site. We sought to establish their prognostic utility in CUP.

**Method:** A prospective data collection was undertaken of patients with provisional or confirmed CUP (using NICE guideline definitions) referred to the CUP service at the Edinburgh Cancer Centre between 2010-2019. Patients with favourable clinico-pathological subgroups were excluded. mGPS at the time of clinical diagnosis was recorded. Survival, defined as the date of biopsy until death, or censorship if alive, was calculated.

**Results:** Data were available for 247 patients. Median survival was 2.2 months. 87% of patients had an elevated CRP (>10mg/l) at diagnosis. mGPS stratified median survival from 11.7 months with mGPS 0 to 1.6 with mGPS 2 (p<0.001). 77% of patients with mGPS 0 were alive at three months compared to only 30% of those with mGPS 2.

**Conclusion:** mGPS, a biomarker of the systemic inflammatory response, is a strong prognostic factor in patients with ‘poor prognosis’ CUP. This simple score, derived from routine blood tests, could be used alongside clinical assessment, standardised investigation and performance status to provide additional objective prognostic information. This may facilitate discussions with patients and assist decisions regarding further investigations and treatment. In patients with mGPS of 2 it may support early conversations about advanced care planning. We would advocate future work validating this finding in other cohorts of CUP patients, and, if supported, incorporating this biomarker into CUP clinical pathways and trials.

**Corresponding author:** Rebekah Patton

## Treatment

### 41. Bioelectical impedance changes during chemotherapy for early breast cancer: The Cando-2 study

#### Stephen Wootton^1^

##### ^1^Faculty of Medicine, University of Southampton, Southampton, UK

**Background:** Excess adiposity and/or lack of lean tissue may be important determinants of chemotherapy outcomes. Bioelectrical Impedance Analysis (BIA) appears readily accessible in clinical settings but changes in hydration during treatment may violate the core assumptions that predict body composition. The measured values of resistance, reactance and phase angle remain secure and may in themselves differentiate between patients in terms of their resilience or response to treatment. The aim of this study was to better characterise the changes in impedance measures in women with early breast cancer during six cycles of standard neo/adjuvant chemotherapy (CANDO-2).

**Method:** Female patients with stage 1-3 invasive breast cancer were recruited from the breast oncology clinic at University Hospital Southampton between September 2014 and September 2015 and received standard-of-care neo/adjuvant chemotherapy (6 x 3 weekly cycles FEC100-T100). Segmental BIA (Seca mBCA515) was measured in 29 women prior to each cycle and after the 6^th^ cycle; impedance measurements were expressed as standard deviation scores (SDS) against device-specific healthy reference population values.

**Results:** The mean SD scores for resistance at 50 kHz (R5), reactance at 50kHz (Xc50), impedance ratio (R200:R5) and phase angle (PhA) at baseline were all within one SD of the median for the reference population. With each cycle of chemotherapy the mean R50 Z score rose progressively, especially from cycle 4 when docetaxel was commenced (P<0.01) with a corresponding fall in IR reflecting an increase in ECW and oedema. Mean Xc50 and PhA SD scores fell markedly with successive cycles reflecting loss of cellular integrity (P<0.01) with more than half of the patients with SD values greater than -2SD by the end of treatment.

**Conclusion:** Impedance measures offer the opportunity to objectively characterise systemic changes in physiological and metabolic state during treatment and may mark important differences between patients in their resilience to chemotherapy.

**Disclosure:** Funded by Breast Cancer Now, London, UK

**Corresponding author:** Stephen Wootton

### 42. Assessment of the effect of oncolytic virotherapy in combination with cavitational ultrasound in the treatment of colorectal liver metastases using a precision cut tumour slice model

#### Marcos Kostalas^1^, Joshua Owen^2^, Claudia Hill^2^, Christopher Smith^3^, Nicola Annels^3^, Robert Carlisle^2^, Hardev Pandha^3^

##### ^1^Royal Surrey County Hospital, Guildford, UK, ^2^University of Oxford, Oxford, UK, ^3^University of Surrey, Guildford, UK

**Background:** Oncolytic virotherapy is a powerful emerging tool in the treatment of cancer. Clinical trials have confirmed the therapeutic effects of oncolytic virotherapy in the treatment of multiple solid tumours. A limitation of this novel treatment is the restricted ability of oncolytic viruses to reach and to penetrate target tumours following intravenous administration. One proposed method of overcoming this is through the concurrent application of ultrasound and specialised cavitational nuclei that expand and violently collapse at specific frequencies enabling greater tissue penetration of anti-cancer agents.

**Method:** Tissue cores, up to 8mm in diameter were obtained from patients with colorectal liver metastases. Cores were sliced using a vibrating microtome to 300µm thickness. These were then treated with oncolytic vaccinia virus. Ultrasonic exposures were carried out using the System for Acoustic Transfection (SAT) chamber. This system was based on prior design but modified to allow a decrease in the exposure area for the prepared tumour slices.

**Results:** Initial experiments found that vaccinia virus in combination with cavitational ultrasound and sulphur hexafluoride microbubbles significantly increased staining for cleaved caspase 3 in treated organotypic cultures of colorectal liver meatsatases compared with vaccinia virus alone. The study also found that the combination of oncolytic virus treatment and cavitational ultrasound enabled the utilisation of lower viral concentrations whilst maintaining similar levels of staining for cleaved caspase 3 and therefore virus activity compared with higher concentrations of virus.

**Conclusion:** Overall, we found that the combined treatment of colorectal liver metastases with vaccinia virus and exposure to cavitational ultrasound and sulphur hexafluoride microbubbles improved the anti-cancer effects of oncolytic vaccinia virus. Combining this treatment with oncolytic virotherapy is a promising technique to improve the anti-cancer effect following the systemic administration of oncolytic vaccinia virus.

**Disclosure:** Funded by Liver Cancer Surgery Appeal, Guildford, UK

**Corresponding author:** Marcos Kostalas

### 43. Addressing the variation in adjuvant chemotherapy treatment for colorectal cancer (CRC): can a regional intervention promote national change?

#### Daniel Swinson^1^, John C. Taylor^2^, Jenny F. Seligmann^1,3^ Rebecca Birch^2,3,4^, Alice Dewdney^5^, Hannah Rossington^2,3,4^, Victoria A. Brown^6^, Jo Dent^7^, Philip Quirke^1,8^, Eva J. A. Morris^2,3,4,^

##### ^1^St James's University Hospital, Leeds, UK, ^2^Leeds Institute of Data Analytics, University of Leeds, Leeds, UK,^3^Leeds Teaching Hospitals Trust, Leeds, Leeds, UK, ^4^Cancer Epidemiology Group, Leeds, UK, Section of Epidemiology and Biostatistics, Leeds, UK, Leeds Institute of Cancer & Pathology, Leeds, UK, ^5^Department of Radiotherapy, Weston Park Hospital, Sheffield, UK, Sheffield Teaching Hospitals, Sheffield, UK, ^6^Queen’s Centre for Oncology and Haematology, Hull, UK, Hull University Hospitals NHS Trust, Hull, UK, ^7^Department of Oncology, Huddersfield Royal Infirmary, Calderdale and Huddersfield NHS Foundation Trust, Huddersfield, UK, ^8^Section of Pathology and Tumor Biology, Leeds, UK, Leeds Institute of Cancer & Pathology, Leeds, UK

**Background:** Analysis of routine population-based data has previously shown that surgery for patients with rectal cancer can vary widely. Through access to the Systemic Anti-Cancer Treatment (SACT) database we have quantified variation in adjuvant chemotherapy across England and in detail across a large representative region (Yorkshire and Humber).

**Method:** National Cancer Registry and Analysis Service (NCRAS) provided data on individuals aged ≥18 years with stage II and III CRC who underwent major resection from 01/01/14 – 31/03/16. Chemotherapy data was obtained from the SACT dataset. Rates of chemotherapy were calculated from multilevel mixed logistic regression and adjusted for age, sex, socioeconomic status, Charlson comorbidity index score and stratified by tumour stage and site. A questionnaire addressing different clinical scenarios was sent to oncologists across the region.

**Results:** The national adjusted chemotherapy treatment rate ranged from 2% to 43% & from 19% to 80% for patients with stage II and stage III cancers. Larger variation was seen for rectal than colon cancer, 10% to 70% vs 14% to 60%. Similar variation was seen in region and across subgroups. A regional questionnaire obtained responses from 15 of 16 MDTs. Widest variation in opinions were observed for high risk stage II patients both with deficient and proficient mismatch repair tumours and stage IIIB patients over the age of 70.

**Conclusion:** Variation is seen across England in the use of adjuvant chemotherapy. Open discussion in our region has enabled consensus agreement on an algorithm for colon cancer, with one pending for rectal cancer.

**Table:** 

		Percentage received chemotherapy	Percentage of treated received combination-chemotherapy
Stage	Stage II	5% – 28%	0% – 63%
	Stage III	41% – 73%	45% – 79%
Site	Colon	27% – 47%	31% – 72%
	Rectal	17% – 59%	30% – 87%
Age	Age<70	33% – 68%	48% – 91%
	Age≥70	12% – 38%	9% – 63%
Prior radiotherapy	No	20% – 58%	29% – 100%
	Yes	14% – 80%	0% – 85%

**Disclosure:** Funded by Yorkshire Cancer Research Charity, Harrogate, UK

Table 1. Abstract 43.

**Corresponding author:** Daniel Swinson

### 44. Ad5_NULL_-A20 – a precision virotherapy that efficiently and selectively targets αvβ6 positive cancers following intravenous administration

#### Alan Parker^1^, James Davies^1^, Gareth Marlow^1^, Hanni Uusi-Kerttula^1^, Gillian Seaton^1^, Luke Piggott^1^, Richard Clarkson^1^, John Chester^1^

##### ^1^Cardiff University, Cardiff, UK

**Background:** Oncolytic virotherapies hold immense promise as anti-cancer agents due to their ability to self-amplify within tumours, lyse cells inducing immunogenic cell death, and express therapeutic modalities encoded within the viral genome. To develop a tumour selective adenovirus (Ad), we refined the Ad capsid to ablate all native means of infection, generating the basal vector, Ad5_NULL_. We incorporated a peptide, A20 (NAVPNLRGDLQVLAQKVART), that binds the tumour selective integrin, αvβ6, with high affinity, thus generating the “precision virotherapy” Ad5_NULL_-A20. Ad5_NULL_-A20 demonstrated ten-million-fold reduction in hepatic uptake following intravenous administration, and efficiently purged peritoneal metastases following intraperitoneal delivery in an in vivo model of ovarian cancer.

**Method:** We evaluated the ability of Ad5_NULL_-A20 to infect and kill αvβ6^+^ pancreatic and triple negative breast cancer (TNBC) cell lines in vitro, using reporter assays to quantify infectivity, and viability assays to monitor cell survival. We generated orthotopic, PDX models of TNBC and investigated the ability of Ad5_NULL_-A20 to home to tumours, quantifying viral genomes recovered from tumours and other organs 72 hours following intravascular delivery.

**Results:** We identified 7/9 pancreatic and 2/4 TNBC lines to be αvβ6^+^. Ad5_NULL_-A20 selectively transduced αvβ6^+^ cells, the oncolytic version killing cells in an αvβ6 dependent fashion. To evaluate systemic targeting of Ad5_NULL_-A20 to tumours, mice were orthotopically implanted with TNBC PDX tumours expressing low/medium/high levels of αvβ6. qPCR revealed remarkably improved tumour: liver ratios for Ad5_NULL_-A20 versus unmodified Ad5 in all tumour types tested, ranging from >50 fold in mice with αvβ6^low^ PDX to >160 fold in αvβ6^high^ PDX model. Viral genomes recovered from tumours increased as a function of αvβ6 positivity.

**Conclusion:** Ad5_NULL_-A20 represents an exciting platform with significant potential to treat αvβ6-expressing cancers either by intravenous or intraperitoneal approaches. Ad5_NULL_-A20 is well suited to arming with immunological transgenes for added efficacy, and for onward clinical translation.

**Disclosure:** Funded by Cancer Research UK, London, UK, Cancer Research Wales, Cardiff, UK

**Corresponding author:** Alan Parker

### 45. Overcoming sorafenib resistance in hepatocellular carcinoma by fasting

#### Jelena Krstic^1^, Isabel Reinisch^1^, Maria Depaoli^1^, Natascha Berger^2^, Christoph Noessing^3^, Markus Galhuber^1^, Ines Anders^4^, Martina Auer^1^, Elisabeth Moyschewitz^1^, Beate Rinner^5^, Martin Pichler^6^, Roland Malli^1^, Andreas Prokesch^1^

##### ^1^Medical University of Graz, Graz, Austria, Gottfried Schatz Research Center for Cell Signaling, Metabolism and Aging, Graz, Austria, ^2^Department of Obstretics and Gynaecology, Medical University of Graz, Graz, Austria, ^3^Cancer Research UK Beatson Institute, London, UK, ^4^Medical University of Graz, Graz, Austria, Core Facility Alternative Biomodels and Preclinical Imaging, Graz, Austria, ^5^Division of Biomedical Research, Medical University of Graz, Graz, Austria, ^6^Division of Oncology, Medical University of Graz, Graz, Austria

**Background:** Fasting is suggested as adjuvant for cancer treatment, since it increases cancer cell sensitivity to chemo- and targeted therapy in various cancer models. The mechanisms of this pleiotropic effect of fasting (i.e. starvation) are not fully elucidated, especially in therapy-resistant cancers. We investigated whether starvation can sensitize therapy-resistant hepatocellular carcinoma (HCC) cells to sorafenib, the only first-line treatment currently in use.

**Method:** HepG2 cells were grown in growth or starvation medium and treated with sorafenib for 24h. Cell viability was analyzed by colorimetric assay and by flow cytometry. Energy metabolism was assayed using XF96 Seahorse analyzer. Components of growth pathways (mTOR and Ras/Raf/MEK/ERK) were analyzed by western blot. p53 knock-out cells were generated using Crispr/Cas9 system. For xenograft assays, cells were injected subcutaneously into the flanks of NMRI-*Foxn1*^*nu*^ mice, which were then treated with vehicle or sorafenib and fed *ad libitum* or intermittently fasted during four weeks.

**Results:** Sorafenib-resistant HepG2 cells were sensitized by starvation, with viability decreasing below 15% after 24h of combined treatment. Similar effect was observed in xenografts: tumors grew at a slower rate only when sorafenib-treated mice were exposed to an intermittent fasting regimen. Mechanistically, bioenergetic profiling suggested abrogated oxidative phosphorylation in HepG2 cells, as an early event in sensitization. Starvation-supported sorafenib also blocked the two major growth pathways, mTOR and Ras/Raf/MEK/ERK. Furthermore, the sensitizing effect of starvation was blunted in p53-deficient cells indicating dependency on p53-mediated cell death.

**Conclusion:** We tested whether starvation can augment the effects of sorafenib in therapy-resistant HCC. Combined treatment could sensitize resistant cancer cells in vitro as well as in xenografts and resulted in complete inhibition of mitochondrial respiration and ATP production, multiple growth pathway blockage, and subsequent p53-mediated cell death. Thus, our study suggests fasting and sorafenib treatment as potential polytherapy for HCC.

**Disclosure:** Funded by Austrian Science Fund (FWF), Vienna, Austria

**Corresponding author:** Jelena Krstic

### 46. Cell culture under perfusion conditions reduces cellular metabolic stress and mimics the in vivo physiological environment in pancreatic cancer

#### Daniel Hughes^1^, Frances Willenbrock^2^, Zahir Soonawalla^3^, Srikanth Reddy^3^, Michael Silva^3^, Somnath Mukherjee^3^, Eric O'Neill^2^

##### ^1^University of Oxford, Oxford, UK, ^2^Department of Oncology, University of Oxford, Oxford, UK, ^3^Oxford University Hospitals, Oxford, UK

**Background:** Tumour responsiveness to chemotherapy in PDAC varies significantly across patients. This reinforces the need to establish an accurate model that preserves the tumour microenvironment in the ex-vivo, allowing a personalized drug screen for patients to identify optimal regime of therapy. Current standard of 2D cell culture is that the media is exchanged at defined time points (i.e. static culture). However, this is not a physiologically representative model as cells are maintained in either a nutrient and substrate ‘hyper-replete’ or deplete environment, rather than an epistatic supply. Moreover, episodic media replacement creates significant and abrupt changes to the culture environment and availability of substrates which induces cellular metabolic stress. We evaluated the role of perfusion culture to establish whether this technique reduced metabolic stress and creates a physiologically representative model.

**Method:** Cells were cultured either in static culture (serving as a control, with media exchange every 72 hours) or in a nutritionally replete condition with a constant low perfusion rate of media exchange over 7 days. Daily media extraction was performed in order to evaluate the metabolic activity and viability of cells. Tissue slices were created from core biopsies and then cultured either in static culture (as control) or under perfusion conditions.

**Results:** Under conventional static culture, a decrease in the quantity of metabolic substrates was observed over time indicating Metabolic activity of cells in static culture was suppressed (noted by down regulation of mTOR pathway products). Under perfusion conditions, glucose concentration was maintained over 7 days, indicating cells were able to maintain metabolic activity. Tissue sections were similarly evaluated for metabolic stress and will be presented.

**Conclusion:** This platform highlights the considerable metabolic stress that cells undergo whilst under conventional static culture. Perfusion culture serves as a technique to reduce cellular metabolic stress in addition to creating a physiologically representative model ex vivo.

**Disclosure:** Funded by Cancer Research UK, London, UK

**Corresponding author:** Daniel Hughes

### 47. 18F-FDG-PET in Guided Dose-Painting with Intensity Modulated Radiotherapy in Oropharyngeal Tumours - Final Toxicity and Disease Outcomes of FiGaRO Phase I Multicentre Feasibility Study

#### Delali Adjogatse^1^, Andriana Michaelidou^2,3,4^, Yae-Eun Suh^1^, Lucy Pike^2,3,4^, Christopher Thomas^2,3,4,5^, Owain Woodley^4^, Beatriz Sanchez Nieto^7^, Tom Rackley^8^, Nachi Palaniappan^8^, Vetrisudar Jayaprakasam^9^, Mererid Evans^8^, Mary Lei^1^, Sally Barrington^2,3,4^, Teresa Guerrero Urbano^1^

##### ^1^Dept of Oncology, Guy's and St Thomas NHS Foundation Trust, London, UK, ^2^King's College London and Guy's and St Thomas PET Centre, London, UK, ^3^School of Biomedical Engineering and Imaging Sciences, King's College London,^4^King's Health Partners, London, UK, ^5^Department of Medical Physics, Guy's and St Thomas NHS Foundation Trust, London, UK, ^6^Medical Physics Dept, Velindre University NHS Trust, Cardiff, UK, ^7^Faculty of Physics, Pontificia Universidad Catolica de Chile, Santiago, Chile, ^8^Dept of Oncology, Velindre University NHS Trust, Cardiff, UK, ^9^Wales Research and Diagnostic PET Imaging Centre, Cardiff, UK

**Background:** The FiGaRO trial assessed the feasibility and safety of delivering a PET-guided radiotherapy (RT) boost to the FDG-avid primary tumour, following 1 cycle of platinum-based induction chemotherapy, in patients with intermediate and high risk locally advanced oropharyngeal cancer.

**Method:** Patients underwent a planning 18FDG-PET-CT scan, immobilised in the treatment position, after one cycle of induction chemotherapy. The volume of persistent FDG-avidity in the primary tumour was escalated to 71.5Gy/30# (75.85Gy BED), delivered using a simultaneous integrated boost (SIB-IMRT) technique. The clinical radical (primary/nodal) and elective (nodal) volumes were treated to 65Gy/30# and 54Gy/30# respectively. RT was delivered with concomitant platinum-based chemotherapy (following 2 cycles of induction chemotherapy). The primary outcome was incidence of radiation-induced mucosal ulceration at 12 months post-treatment, with an incidence of 10% or less deemed acceptable. Secondary outcomes included acute and other late toxicities and disease outcomes (NCICTCAEv.4.0, RTOG and LENTSOMA scales).

**Results:** Twenty-four patients were treated between 2014 and 2018, in 2 UK centres. Median follow-up was 36 months (range 4-56). Pre-defined planning target volume and organ at risk dose constraints were met in all cases. All patients completed treatment. There were no incidents of acute grade 4 toxicity. There were no cases of persistent mucosal ulceration at 12 months. RTOG grade 3 mucosal toxicity at 12 months was recorded in 1 patient (atrophy and telangiectasia). This patient had mucosal ulceration at 6 months which resolved by 12 months. One patient was feeding-tube dependent at 12 months, however mucosal integrity was intact. Local control was 83.3% (n=20), and loco-regional control was 79.2% (n=19). Overall survival at 1- and 3-years was 87.5% and 82.9% respectively. Disease-free survival was 83.3% at 1 year and 78.95% at 3 years.

**Conclusion:** PET-guided dose escalated chemo-radiotherapy, following induction chemotherapy, is feasible and associated with favourable mucosal toxicity rates.

**Disclosure:** Funded by Oncology Research Fund, Guy's and St Thomas NHS Foundation Trust, London, UK

**Corresponding author:** Delali Adjogatse

### 48. Does recruitment to cancer clinical trials at the Leicester Royal Infirmary reflect the ethnic diversity of those receiving systemic anticancer therapy?

#### Adrian Nicholson^1^, Mohammed Karolia^1^, Emily de Groot^2^, Sarah Nicholson^1^, Devinder Dhillon^1^, Lynne Howells^3^, Harriet Walter^3^, Anne Thomas^3^

##### ^1^University Hospitals Leicester, Leicester, UK, ^2^University of Leicester Medical School, Leicester, UK, ^3^Leicester Cancer Research Centre, Leicester, UK

**Background:** Participation in cancer clinical trials has been associated with improved outcome. However, specific barriers to participation exist within racial and ethnic minority populations. According to the 2011 census, Leicester has one of the most diverse population within the UK (8th highest). We sought to determine whether recruitment to cancer clinical trials reflects the racial and ethnic diversity of patients treated with systemic anticancer therapy (SACT) within the Leicester Cancer Centre (LCC).

**Method:** A retrospective review using the SACT Dataset of racial and ethnic diversity of patients (categorised using SACT codes) receiving treatment within a cancer clinical trial was compared to those receiving standard treatment from January 2016 to December 2018.

**Results:** From Jan 2016 - Dec 2018, 11,080 patients received SACT. 1,140 (10.3%) were treated within a clinical trial. Of patients receiving standard SACT: 7,788 (78.4%) were white, 871(8.8%) Asian/Asian British, 163 (1.6%) black/African/Caribbean/Black British, 39 (0.4%) Mixed/Multiple ethnic groups, 87 (0.9%) other and 992 (10.0%) not stated/unknown; versus 908 (79.6%), 96 (8.4%), 10 (0.9%), 5 (0.4%), 6 (0.5%) and 115 (10.1%) respectively treated in clinical trials. Of those treated in a Phase I trial 78.9% were white. The male:female ratio of patients receiving SACT was 1:1. Of those receiving SACT, a greater percent of males received treatment within a clinical trial versus females (13.1% vs 7.46% p=<0.005). The median age of patients treated within a trial was 66 years (range 2-89) and standard SACT 67 years (range 1-100).

**Conclusion:** When compared to SACT, our results demonstrate higher recruitment to trials of white and Asian/Asian British patients. Interestingly, despite a diverse population, 78.5 % treated with SACT were white. Further work is required to identify whether recruitment barriers exist, which may have implications in terms of safety and efficacy of the trial.

**Corresponding author:** Adrian Nicholson

### 49. Identification and validation of natural anti-drug resistant and anticancer stem cell agents in TNBC stem cells

#### Prem Kushwaha^1^, Shashank Kumar^1^

##### ^1^Central University of Punjab, Punjab, India

**Background:** Cancer drug resistance reduces the effect of the drug(s) in cancer cells. Triple-negative breast cancer (TNBC) is an aggressive disease with a poor therapy outcome. Drug resistance and self-renewal properties of stem cells make it an attractive target for anticancer drug discovery. ABC transporter and stemness markers are positively involved in stem cell drug resistance. Nowadays natural remedies are of interest due to lesser side effects and cost-effective. Taken together, there is an urgent need to identify novel natural anti-drug resistant and anticancer agents against TNBC stem cells.

**Method:** Different scientific literature database were used to prepare the list of phytochemicals present in Bulbine spp. (Asphodelaceae). Molecular docking and simulation approach was used to identify the ABC transporter inhibitor from the enlisted phytochemicals. Literature shows that the enlisted phytochemicals are soluble in the polar solvent. Thus, we prepared the methanolic (polar) extract of Bulbine spp. Anti-drug resistance and anticancer activity of the extract was examined by using MTT, drug efflux, and colony formation assay. Further, the therapeutic potential of the extract was studied in terms of apoptosis induction and reduction in stemness markers (Oct4, Sox2, Nanog, and Myc) at the transcriptional level.

**Results:** *In silico* study revealed potent phytochemical showing better binding affinity with ABC transporter in comparison to standard inhibitors. Extract inhibited the mammosphere formation and reduced colony formation in TNBC cells. Treatment showed reduced drug efflux activity, down-regulated stem cell markers and induced apoptosis in the cells.

**Conclusion:** Present *in silico* and in vitro study suggest that Bulbine spp. phytochemicals have anti-drug resistance and anti-cancer stem cell potential. The phytochemicals may act as the lead candidate for drug development against triple negative breast cancer stem cells.

**Disclosure:** Funded by Department of Science and Technology, New Delhi, India

**Corresponding author:** Prem Kushwaha

### 50. Silvestrol sensitises breast cancer cells to radiation

#### Thomas Webb^1^

##### ^1^East Kent Hospitals University NHS Foundation Trust, Kent, UK

**Background:** eIF4A is an RNA helicase that forms part of the machinery of translation initiation. Proteomic analysis demonstrated eIF4A expression to be at least two-fold greater in a radioresistant derivative of T47D breast cancer cells compared to parental cells. Inhibition of eIF4A has previously been shown to resensitise lymphomas to chemotherapeutic agents that cause DNA damage. The objective of this work is to investigate whether small molecule inhibition of eIF4A using silvestrol sensitises breast cancer cells to radiotherapy in tissue culture.

**Method:** T47D cells were incubated in media containing 0 nM to 1 nM silvestrol either for 24 hours prior to irradiation at 0 Gy to 10 Gy (delivered by LINAC) or continually for six days post irradiation. MTT viability and clonogenic assays were used to quantify response.

**Results:** Pre-treatment of T47D cells with 1 nM silvestrol caused a 34% reduction (p = 0.014) in viability on irradiation at 2 Gy compared to treatment with a DMSO control, as assessed by MTT assay. Maintenance of cells in 1 nM silvestrol for six days following irradiation at 2 Gy caused a 58% reduction (p = <0.01) in viability. Clonogenic assays performed on cells maintained in 1 nM silvestrol following irradiation showed a Dose Modifying Factor (DMF) of 1.4 at 10% surviving fraction.

**Conclusion:** Low concentrations of silvestrol sensitise T47D breast cancer cells to radiation with minimal effect on unirradiated cells. This highlights the possible usefulness of eIF4A inhibition in potentiating radiation-induced damage at the tumour site without causing systemic toxicity.

**Disclosure:** Funded by Isaac Schapera Trust, London, UK

**Corresponding author:** Thomas Webb

### 51. Capivasertib plus fulvestrant versus placebo plus fulvestrant in metastatic ER positive breast cancer (FAKTION): A randomised, double-blind, placebo-controlled, phase II trial

#### Robert Jones^1^, Margherita Carucci^2^, Angela Casbard^2^, Rachel Butler^3^, Catherine Bale^4^, Fouad Alchami^5^, Pavel Bezecny^6^, Johnathan Joffe^7^, Sarah Moon^8^, Chris Twelves^9^, Ramachandran Venkitaraman^10^, Simon Waters^1^, Sacha Howell^11^

##### ^1^Velindre University NHS Trust, Cardiff, UK, ^2^Cardiff University, Cardiff, UK, ^3^North Bristol NHS Trust, Bristol, UK, ^4^Betsi Cadwaladr University Health Board, Bangor, UK, ^5^Department of Cellular Pathology, University Hospital of Wales, Cardiff, UK, ^6^Blackpool Teaching Hospitals NHS Foundation Trust, Blackpool, UK, ^7^Royal College of Physicians of London, London, UK, ^8^University Hospitals of Morecambe Bay NHS Trust, Milnthorpe Cumbria, UK, ^9^University of Leeds and St. James's Institute of Oncology, Leeds, UK, ^10^The Ipswich Hospital NHS Trust, Ipswich, UK, ^11^The University of Manchester and The Christie NHS Foundation Trust, Manchester, UK

**Disclosure:** Funded by Cancer Research UK and AZ educational grant

**Corresponding author:** Robert Jones

This abstract has been published previously, see http://abstracts.asco.org/239/AbstView_239_267745.html

### 52. FOxTROT: an international randomised controlled trial in 1052 patients evaluating neoadjuvant chemotherapy for colon cancer

#### Dion Morton^1^, on behalf of the FOxTROT investigators

##### ^1^University of Birmingham, Birmingham, UK

**Disclosure:** Funded by Cancer Research UK, London, UK, Yorkshire Cancer Research, Harrogate, UK, Amgen Pharma, Cambridge, UK

**Corresponding author:** Dion Morton, on behalf of the FOxTROT investigators

This abstract has been published previously, see http://abstracts.asco.org/239/AbstView_239_267235.html and https://ascopubs.org/doi/abs/10.1200/JCO.2019.37.15_suppl.3504

### 53. ABC-06 | A randomised phase III study of Active Symptom Control (ASC) with/without mFOLFOX after progression to Cisplatin-Gemcitabine chemotherapy for patients with advanced biliary tract cancers

#### Angela Lamarca^1^, Daniel H Palmer^2^, Harpreet Singh Wasan^3^, Paul J. Ross^4^, Yuk Ting Ma^5^, Arvind Arora^6^, Stephen Falk^7^, Roopinder Gillmore^8^, Jonathan Wadsley^9^, Kinnari Patel^10^, Alan Anthoney^11^, Anthony Maraveyas^12^, Tim Iveson^13^, Justin S. Waters^14^, Claire Blesing^10^, Safia Barber^15^, David Ryder^15^, John Ramage^16^, Linda Davies^17^, John A. Bridgewater^18^, Juan W Valle^19^

##### ^1^The Christie NHS Foundation Trust, Manchester, UK, ^2^University of Liverpool, Liverpool, UK, ^3^Department of Cancer Medicine, Hammersmith Hospital, London, UK, ^4^Guy's and St Thomas' NHS Foundation Trust, London, UK, ^5^University of Birmingham, Birmingham, UK, ^6^University Hospital of Nottingham NHS Trust, Nottingham, UK, University of Nottingham, Nottingham, UK, ^7^Bristol Haematology and Oncology Centre, Bristol, UK, ^8^Royal Free Hospital, London, UK, ^9^Weston Park Hospital, Sheffield, UK, ^10^Oxford University Hospitals, Oxford, UK, ^11^Leeds Teaching Hospitals NHS Trust, Leeds, UK, ^12^Castle Hill Hospital, Hull, UK, ^13^University Hospital Southampton NHS Foundation Trust, Southampton, UK, ^14^Kent Oncology Centre, Maidstone, UK, ^15^Clinical Trials Unit, University of Manchester, Manchester, UK, ^16^Hampshire Hospitals NHS Foundation Trust, Basingstoke, UK, ^17^Health Economics Department, University of Manchester, Manchester, UK, ^18^University College London Cancer Institute, London, UK, ^19^Institute of Cancer Sciences, University of Manchester, Manchester, UK, The Christie NHS Foundation Trust, Manchester, UK

**Disclosure:** Funded by Cancer Research UK, London, UK, StandUp2Cancer, London, UK, The Alan Morement Memorial Fund, Stansted, UK, The Christie Charity, Manchester, UK, Cholangiocarcinoma Foundation, Herriman, US, European Society For Medical Oncology, Lugano, Switzerland, Sociedad Española de Oncología Médica, Madrid, Spain, Conquer Cancer Foundation, Alexandria, US

**Corresponding author:** Angela Lamarca

This abstract has been published previously, see http://abstracts.asco.org/239/AbstView_239_248927.html

### 54. Should major surgery be offered to elderly patients with early-stage lung cancer? An emulated trial using observational data in England

#### Camille Maringe^1^, Clémence Leyrat^1^, Sara Benitez Majano^1^, Aimilia Exarchakou^1^, Matthew Smith^1^, Bernard Rachet^1^, Aurélien Belot^1^

##### ^1^London School of Hygienen and Tropical Medicine, London, UK

**Background:** Older cancer patients often have suboptimal cancer treatment and poorer cancer outcomes than younger patients: early-stage non-small cell lung cancer (NSCLC) patients diagnosed after 70 years of age in England show up to 60% reduced probabilities of receiving major surgery compared to younger patients. As older cancer patients are generally excluded from clinical trials, the evidence supporting aggressive cancer management is scarce and has to rely on non-randomised studies that are more likely to be prone to bias. For instance, when using observational data to estimate the causal effect of surgery on survival, immortal-time bias is an issue because of the waiting-time between diagnosis and surgery.

**Method:** To measure the causal effect of receiving a major surgery for lung cancer patients aged over 70 years at diagnosis on 1-year survival probability, we emulated a randomised trial using population-based cancer registry data in England, linked to Hospital Episode Statistics and Lung Cancer Audit Data.

**Results:** The inclusion criteria led to the selection of 2309 patients. The intervention arm was surgery within six months following diagnosis compared to no surgery within six months. At diagnosis, patients were cloned and entered both arms. Observations were censored when they deviated from the protocol, and this dependent censoring was accounted for using inverse-probability-of-censoring weights when estimating survival probabilities. There were 83% (95% CI: 81-85) and 71% (95% CI: 68-74) of treated and non-treated patients who survived the first year after diagnosis, respectively. This corresponded to 14 days of life saved in the first year after diagnosis.

**Conclusion:** Our results suggest a strong benefit on survival of surgery among older patients with early-stage NSCLC. We used an innovative study design, enabling us to control for confounding and immortal-time bias, and allowing a transparent reporting of the methods.

**Disclosure:** Funded by Cancer Research UK grant C7923/A18525, London, UK

**Corresponding author:** Camille Maringe

### 55. Efficacy and safety of the combination of nivolumab (NIVO) plus ipilimumab (IPI) in patients with symptomatic melanoma brain metastases (MBM; CheckMate 204)

#### Hussein Tawbi^1^, Peter Forsyth^2^, F. Stephen Hodi^3^, Christopher Lao^4^, Stergios Moschos^5^, Omid Hamid^6^, Michael B. Atkins^7^, Karl Lewis^8^, Reena P. Thomas^9^, John A. Glaspy^10^, Sekwon Jang^11^, Alain Algazi^12^, Nikhil I. Khushalani^2^, Michael A. Postow^13^, Anna C. Pavlick^14^, Marc Ernstoff^15^, David A. Reardon^3^, Agnes Balogh^16^, Jasmine Rizzo^16^, Kim Margolin^17^

##### ^1^University of Texas MD Anderson Cancer Center, Houston, US, ^2^Moffitt Cancer Center and Research Institute, Tampa, US, ^3^Dana-Farber Cancer Institute, Boston, US, ^4^University of Michigan, Ann Arbor, US, ^5^University of North Carolina Lineberger Comprehensive Cancer Center, Chapel Hill, US, ^6^The Angeles Clinic and Research Institute, Los Angeles, US, ^7^Georgetown Lombardi Comprehensive Cancer Center, Washington, US, ^8^University of Colorado Comprehensive Cancer Center, Aurora, US, ^9^Stanford University Hospital, Stanford, US, ^10^Jonsson Comprehensive Cancer Center University of California, Los Angeles, US, ^11^Inova Schar Cancer Institute, Falls Church, US, Virginia Commonwealth University, Richmond, US, ^12^University of California–San Francisco, San Francisco, US, ^13^Memorial Sloan Kettering Cancer Center and Weill Cornell Medical College, New York, US, ^14^New York University Langone Medical Center, New York, US, ^15^Roswell Park Cancer Institute, Buffalo, US, ^16^Bristol-Myers Squibb, New York, US, ^17^City of Hope, Duarte, US

**Disclosure:** Funded by Bristol-Myers Squibb Foundation, New York, US

**Corresponding author:** Peter Forsyth

This abstract has been published previously, see https://abstracts.asco.org/239/AbstView_239_259645.html

### 56. Xentuzumab (BI 836845), an IGF-neutralizing antibody, combined with exemestane and everolimus in hormone receptor-positive (HR+) locally advanced/metastatic breast cancer: Randomized Phase 2 Results

#### John Crown^1^, Marie-Paule Sablin^2^, Javier Cortés^3,7^, Jonas Bergh^4^, Seock-Ah Im^5^, Yen-Shen Lu^6^, Noelia Martínez^7^, Patrick Neven^8^, Keun Seok Lee^9^, Serafín Morales^10^, J. Alejandro Pérez-Fidalgo^11^, Douglas Adamson^12^, Anthony Goncalves^13^, Aleix Prat^14^, Guy Jerusalem^15^, Laura Schlieker^16^, Rosa-Maria Espadero^17^, Thomas Bogenrieder^18^, Dennis Chin-Lun Huang^19^, Peter Schmid^20^

##### ^1^St Vincent University Hospital, Dublin, Ireland, ^2^Institut Curie, Paris, France, ^3^Vall d’Hebron Institute of Oncology (VHIO), Barcelona, Spain, ^4^Karolinska Institutet and Karolinska University Hospital, Stockholm, Sweden, ^5^Seoul National University Hospital, Seoul, South Korea, ^6^National Taiwan University Hospital, Taipei, Taiwan, ^7^IOB Institute of Oncology, Quironsalud Group, Madrid and Barcelona, Spain, ^8^UZ Leuven, Campus Gasthuisberg, Lueven, Belgium, ^9^National Cancer Center, Goyang, South Korea, ^10^Hospital Universitario Arnau de Vilanova de Lleida, Lleida, Spain, ^11^Hospital Clinico Universitario Valencia, Spain, Biomedical Research Institute INCLIVA, Valencia, Spain, el Centro de Investigación Biomédica en Red de Cáncer, Madrid, Spain, ^12^Tayside Cancer Centre, Ninewells Hospital, Dundee, UK, ^13^Institut Paoli Calmettes, Marseille, France, ^14^Hospital Clínic de Barcelona Servicio de Oncología Médica, Barcelona, Spain, ^15^Centre Hospitalier Universitaire de Liège, Belgium, Liège University, Liège, Belgium, ^16^External statistician on behalf of Boehringer Ingelheim Pharma GmbH & Co. KG, Bracknell, UK, Staburo GmbH & Co. KG. Staburo GmbH, Munich, Germany, ^17^Boehringer Ingelheim España S.A, Barcelona, Spain, ^18^Boehringer Ingelheim RCV, Vienna, Austria, ^19^Boehringer Ingelheim Taiwan Limited, Taipei, Taiwan, ^20^Centre for Experimental Cancer Medicine, Barts Cancer Institute, London, UK, Queen Mary University of London, London, UK

**Disclosure:** Funded by Boehringer Ingelheim, Bracknell, UK

**Corresponding author:** Peter Schmid

This abstract has been published previously, see p. 1484, Publication Number**:** P6-21-01 https://www.sabcs.org/SABCS/2018/AllAbstracts_2018-12-03_Updated.pdf

### 57. PARP inhibition causes replication stress in preclinical models of high risk neuroblastoma and synergises with inhibition of ATR

#### Harriet Southgate^1^, Nicola J Curtin^1^, Deborah A Tweddle^1^

##### ^1^Newcastle University

**Background:** Neuroblastoma (NB) is the commonest extra-cranial malignant solid tumour of childhood and one of the most difficult to cure. Around 50% of high-risk NBs have *MYCN* oncogene amplification (MNA) that promotes rapid DNA replication, leading to errors and replication stress (RS). Cells with RS are acutely dependent on the DNA damage sensor kinase ATR. PARP inhibition results in unrepaired single strand DNA breaks progressing to replication, further increasing RS. We hypothesise that combining PARP and ATR inhibition will lead to greater cytotoxicity due to increased RS. Aim: To assess synergism between PARP inhibition and ATR inhibition in high risk NB cell lines and to measure RS.

**Method:** Human NB cell lines: SHSY5Y and SKNAS (non-MNA), and NGP and N20_R1 (MNA). The PARPi olaparib and the ATRi VE-821 were used. CHK1^S345^ and H2AX^S129^ phosphorylation was assessed using Western blotting to determine ATR activity and RS respectively. RS was also determined by □H2AX foci formation using immunofluorescent microscopy. Cytotoxicity was assessed by XTT cell proliferation assay (Roche) and colony formation assay.

**Results:** Olaparib (5 µM) treatment increased CHK1^S345^ and H2AX^S129^ phosphorylation after treatment for 24 hours in all cell lines. H2AX^S129^ phosphorylation was further increased with the addition of VE-821 (1 µM). ATR inhibition prevented CHK1^S345^ phosphorylation, as expected. The number of □H2AX foci exhibited in the cell lines by immunofluorescence increased after treatment with olaparib (1 µM) which was further increased with the addition of VE-821 (1 µM). In cytotoxicity assays, combination index analysis (Calcusyn) showed that ATR inhibition by VE-821 is synergistic with olaparib at sub lethal concentrations (<1 µM) (CI value 0.04-0.89), although this effect is lost at higher concentrations.

**Conclusion:** ATR inhibition by VE-821 is synergistic with olaparib at sub lethal concentrations (<1 µM) and further increases the replication stress caused by PARP inhibition.

**Disclosure:** Funded by Children's Cancer and Leukaemia Group, Leicester, UK, Little Princess Trust, Hereford, UK

**Corresponding author:** Harriet Southgate

### 58. Making Outcome-Based Payment for Cancer Medicines a Reality in the NHS

#### Amanda Cole^1^, Patricia Cubi-Molla^1^, Jack Pollard^2^, Duncan Sim^3^, Richard Sullivan^4^, Jon Sussex^2^, Paula Lorgelly^4^

##### ^1^Office of Health Economics, London, UK, ^2^RAND Europe, Cambridge, UK ^3^Cancer Research UK, London, UK, ^4^King's College London, London UK

**Background:** Many cancer medicines are made available on the NHS based on a price discount agreed between the NHS and the manufacturer. However, uncertainty in the evidence base and associated value proposition can prolong price negotiation and risk delaying patient access. More flexible ways for the NHS to pay for medicines, such as Outcome-Based Payment (OBP), which links a medicine’s price to NHS patients’ treatment outcomes, could provide a solution. Cancer Research UK and Greater Manchester Health and Social Care Partnership commissioned the Office of Health Economics, RAND Europe, and King’s College London to explore the feasibility of introducing an OBP approach for new cancer drugs in England.

**Method:** Literature reviews of patient outcome metrics and existing payment schemes linking outcomes with pricing. Interviews with NHS and government stakeholders, healthcare professionals, and pharmaceutical industry representatives. Focus groups and survey with people affected by cancer. Preliminary review of NHS Health and Cancer data.

**Results:** Implementation of OBP in the NHS is possible and desirable for some new cancer medicines and is most relevant where substantial uncertainty remains about the effectiveness of a medicine based on clinical trial data. There is no single ‘best buy’ OBP scheme to apply generally. Variation in schemes will be contingent upon factors such as cancer site and stage, patient demographics and the nature of the evidence uncertainty. Future OBP schemes in the NHS should include both clinical and quality-of-life patient outcome measures: survival; disease progression, relapse or recurrence; long-term side effects; and return to normal activities. Issues around real-world data present key barriers to implementing OBP in the NHS.

**Conclusion:** Significant advances in understanding the principles based on which an OBP approach might be used for new cancer medicines for the NHS in England have been achieved. Future research will establish the necessary steps for implementing a pilot OBP scheme.

**Disclosure:** Funded by Cancer Research UK, London, UK; Greater Manchester Health and Social Care Partnership, Manchester, UK

**Corresponding author:** Duncan Sim

### 59. Real world treatment sequencing patterns in secondary breast cancer (SBC): Pathway visualisation using national datasets

#### Ashley Horne^1^, Maria McMenemy^2^, Holly Ennis^3^, Lauren Murdoch^3^, Grace Ding^1^, Olga Oikonomidou^1^, Caroline Michie^1^, Larry Haywood^1^, David Cameron^1^, Christina Lilley^1^, Peter Hall^4^, Alison Stillie^1^, Aisling Hennessy^1^, Frances Yuille^1^

##### ^1^Edinburgh Cancer Centre, Western General Hospital, Edinburgh, UK, ^2^Lothian Analytical Services, NHS Lothian, Edinburgh, UK, ^3^Edinburgh Clinical Trials Unit, University of Edinburgh, Edinburgh, UK, ^4^Edinburgh Cancer Research Centre, University of Edinburgh, Edinburgh, UK.

**Background:** Treatment pathways in metastatic breast cancer are complex. The accelerated adoption of new medicines has resulted in an uncertain evidence base supporting their use. Uncertainties are related to the mismatch between trial-recruited and real-world populations and variation in the order of sequential drugs. Published examples describing real-world practice in SBC are scarce, mainly due to the complexity of the clinical pathways that rely on a mixture of chemotherapy, endocrine therapy and biologicals, often over a long period. We demonstrate how new opportunities in routine healthcare data allow a highly granular description of real-world treatment pathways and how this varies in light of patient (pt) case-mix.

**Method:** Scottish nationally available data source datasets for linkage included the National Cancer Registry, Scottish Morbidity Record, the National Cancer Quality Audit and the national Prescribing Information System. Scottish CHI number was the universal identifier for linkage. Key baseline characteristics included age, de-novo presentation, prior adjuvant treatments, co-morbidities, concomitant medications and socioeconomic status. Targeted and random sampling manual review was used to quantify missing data. R version 3.6 was used for analysis.

**Results:** 345 pts were identified of which 276 had ER+HER2- SBC between 2012-2017. First line therapy included 68% (235 patients) endocrine therapy, 17% (59 pts) chemotherapy, 14% (50 pts) received no treatment. Subsequent treatment decisions, including best supportive care and death, have been tracked to identify 70 unique pathways with up to 8 lines of treatment. Graphical representation of treatment pathways is made using Sankey plots. Detailed data quality reports describe missing data rates over time and a comprehensive guide for analysts has been produced as a wiki [https://blogs.ed.ac.uk/canceroutcomes/edinburgh-cancer-informatics-wiki/].

**Conclusion:** It is now possible to describe treatment sequences using routine, nationally available administrative healthcare data. Pathways are complex and do not always conform to standard guidelines. Interpretation requires modern graphical visualisation methods.

**Disclosure:** Funded by NHS Lothian, Edinburgh, UK, The Association of the British Pharmaceutical Industry (ABPI) Scotland Collaboration Group (SCG), Edinburgh, UK

**Corresponding author:** Ashley Horne

### 60. The continual reassessment method for phase I clinical trial design: is it always more efficient than the 3+3?

#### Samantha Hinsley^1^

##### ^1^Clinical Trials Unit, Cancer Research UK, Glasgow, UK

**Table:** 

Design	Prior	Cohort size	P(Correct MTD)	
			Mean	Range
3+3	N/A	3	45.7	29.8–63.2
CRM	1	3	49.3	28.3–92.2
		2	39.1	11.9–84.2
	2	3	36.1	9.7–81.2
		2	38.7	10.1–83.8
	3	3	45.8	19.9–56.0
		2	41.2	20.9–62.9

**Background:** It is becoming widely suggested that the continual reassessment method (CRM) is superior to the 3+3 for phase I clinical trials for reaching the maximum tolerated dose (MTD) more efficiently and accurately. The literature in this area is growing, but the majority of comparisons consider a high number of dose levels. In many phase I trials, the number of dose levels may only be 3, and it is unclear whether the CRM is beneficial in this situation.

**Method:** Simulations were performed to compare the 3+3 and CRM, using 3 dose levels, maximum sample size=18 and upper acceptable limit of toxicity=1/3 (all in line with a 3+3 trial of 3 dose levels). Three different priors and cohort sizes of 2 and 3 were investigated for the CRM, and 10000 trials were simulated under six different “true” scenarios of dose limiting toxicity rates.

**Results:** Simulations show that the 3+3 and CRM are largely comparable, and no benefit of the CRM is observed in this setting (brief summary of data in Table 1). This is because the accuracy of the CRM relies heavily on the prior, and where this is not close to the true scenario, the CRM can perform poorly.

**Conclusion:** From these results, we can argue that for a trial considering 3 dose levels, where the prior rates of toxicities are unclear, the CRM is not more efficient unless the sample size is increased. Therefore, the growing view that the CRM is superior to the 3+3 across the board is not always accurate.

Table 1. Abstract 60. Average probability of choosing the correct MTD across all true scenarios

**Disclosure:** Funded by Clinical Trials Unit, Cancer Research UK, Glasgow, UK

Corresponding author: Samantha Hinsley

### 61. Comparisons of overall survival in women diagnosed with early stage cervical cancer during 2013-2016, treated by radical hysterectomy using minimal access surgery (MAS) or open approach in England

#### Andy Nordin^1^, Rebecca Elleray^2^, Robin Crawford^3^, John Broggio^2^, Lucy Elliss-Brookes^2^, Georgios Lyratzopoulos^4^

##### ^1^East Kent Gynaecological Oncology Centre, Queen Elizabeth the Queen Mother Hospital, Margate, UK, ^2^Public Health England, London, UK, ^3^Cambridge University Hospitals NHS Foundation Trust, Cambridge, UK, ^4^University College London, London, UK

**Background:** Given published previous RCT and other evidence suggesting survival differences by type of surgical approach for radical hysterectomy in early stage cervical cancer,^1,2^ we analysed population-based cancer registration data in England.^3^

**Method:** Cohort definition: Women diagnosed during 2013-2016 with early stage diagnosis (stage IA2, IB, IB1) of cervical cancer, treated by radical hysterectomy using either open or minimal access surgery (MAS) approach. Cancer registration data were linked to Hospital Episodes Statistics data to determine the surgical approach; to chemotherapy and radiotherapy data to define whether patients also received adjuvant therapy; and to mortality data for survival follow-up. Outcome: Overall survival. Patients were followed up to 31/12/2017 (follow-up range 0.35-4.99 years, median/mean follow-up 3.06/3.04 years).

**Results:** Among 929 women in the study cohort, 564 (61%) were treated by MAS and 365 (39%) by open surgery. Patient characteristics (including age, diagnostic route, stage, deprivation and comorbidity) were similar in each approach group. MAS use increased from 48% in 2013 to 74% in 2016. In Kaplan-Meier analysis, women treated by MAS had significantly worse outcomes at 4 years (98.3% open vs 93.9% minimal access surgery, p=0.03) and subsequently. In unadjusted Cox regression analysis, MAS was associated with a hazard ratio (compared to open access) of 3.3 (p=0.009), becoming slightly larger in multivariable Cox regression (hazard ratio=4.0, p=0.007).

**Conclusion:** Overall survival in women treated with radical hysterectomy for early stage cervical cancer is very high, but in-keeping with prior published evidence,^1,2^ this population-based analysis of ‘real-world’ unselected patients indicates that women treated by MAS may have inferior survival than those treated with open surgery. The analysis has been communicated with stakeholders including NHS England and the British Gynaecological Cancer Society providing evidential support for their public statement.^4^ Further analysis of this data is ongoing.

**Acknowledgement:** This work uses patient data collated by Public Health England.

**Disclosure:** Funded by Public Health England, London, UK

**Corresponding author:** Lucy Elliss-Brookes

### 62. Factors associated with Emergency Admissions for Cancer Patients in Last year of life in a UK region

#### Victoria Cairnduff^1^, Laura Dwyer^1^, Colin Fox^1^, Gregory Fallica^2^, Kelly Shiell-Davis^2^, Anna Gavin^1^

##### ^1^Northern Ireland Cancer Registry, Belfast, UK, ^2^Macmillan Cancer Support, London, UK

**Background:** Emergency admissions towards end-of-life may indicate gaps in routine cancer care. This project aims to examine the demographic, disease and environmental characteristics of people dying with cancer admitted as an emergency in the last year of life to provide information to improve services.

**Method:** Data on all cancer deaths in N.Ireland (NI) in 2015 were linked with hospital episodes relating to emergency admissions in the last year of life. Logistic Regression was carried out using “at least one emergency admission recorded” as the outcome variable.

**Results:** Of 4,224 people dying of cancer in NI in 2015, 74.2%; (n=3,134) had at least one emergency admission is the last year of life recorded and 36.6% (n=1,546) in the last 28 days of life. The factors with a significant positive association to having an emergency admission in last year of life (model I) were tumour site, stage at diagnosis, time from diagnosis to death and place of death and the last 28 days of life (model II) time from diagnosis to death, place of death and reason for last admission.

**Conclusion:** Emergency admission in last year of life is common for cancer patients, especially in the last month where the risk of hospital death was highest. Differences exist by cancer type and age, but no differences by deprivation or rurality were observed. A further economic analysis is now underway to establish estimated costs associated with emergency admissions at end-of-life. These findings will help inform future changes in emergency care for cancer patients at end-of-life in NI.

**Acknowledgements:** The N.Ireland Cancer Registry is funded by the Public Health Agency of N.Ireland.This research has been funded by Macmillan Cancer Support as part of the Macmillan-NICR Partnership. This work uses data provided by patients and collected by the health service as part of their care and support.

**Disclosure:** Funded by Macmillan Cancer Support*, London, UK*

**Corresponding author:** Anna Gavin

### 63. Optimizing Chemotherapy for Frail and Elderly Patients with Advanced Gastroesophageal Cancer (aGOAC): the GO2 Phase III Trial

#### Peter Hall^1^, Daniel Swinson^2^, Simon Lord^3^, Helen Marshall^4^, David Cairns^4^, Sharon Ruddock^4^, Emma Batman^4^, Galina Velikova^4^, Russell Petty^5^, Justin Waters^6^, Jonathan Wadsley^7^, Stephen Falk^8^, Catherine Handforth^9^, Rajarshi Roy^10^, Mano Joseph^11^, Konstantinos Kamposioras^12^, Jonathan Nicoll^13^, Tania Tillett^14^, Sebastian Cummins^15^, Simon Grumett^16^, Zuzana Stokes^17^, Tom Waddell^18^, Anirban Chatterjee^19^, Angel Garcia^19^, Christine Allmark^20^, Matthew Seymour^4^

##### ^1^University of Edinburgh, Edinburgh, UK, ^2^Leeds Teaching Hospitals NHS Trust, Leeds, UK, ^3^University of Oxford, Oxford, UK, ^4^University of Leeds, Leeds, UK, ^5^University of Dundee, Dundee, UK, ^6^Kent Oncology Centre, Maidstone, UK, ^7^Western Park Hospital, Sheffield, UK, ^8^Bristol Haematology and Oncology Centre, Bristol, UK ^9^University of Sheffield, Sheffield, UK, ^10^Castle Hill Hospital, Hull, UK, ^11^The Royal Wolverhampton NHS Trust, Wolverhampton, UK, ^12^Mid Yorkshire Hospitals NHS Trust, Wakefield, UK ^13^North Cumbria University Hospitals NHS Trust, Carlisle, UK ^14^Royal United Hospital Bath NHS Trust, Bath, UK, ^15^Royal Surrey County Hospital NHS Foundation Trust, Guilford, UK, ^16^The Dudley Group NHS Foundation Trust, Dudley, UK, ^17^United Lincolnshire Hospitals NHS Trust, Lincoln, UK ^18^The Christie NHS Foundation Trust, Manchester, UK, ^19^The Shrewsbury and Telford Hospital NHS Trust, Shrewsbury, UK, ^20^National Cancer Research Institute Consumer Forum, London, UK

**Disclosure:** Funded by Cancer Research UK, London, UK

**Corresponding author:** Peter Hall

This abstract has been published previously, see https://abstracts.asco.org/239/AbstView_239_259067.html

### 64. Evaluation of older patients in early phase clinical trials

#### Jessica Lowe^1^, Rosie Lauste^2^, Tine Descamps^3^, Matthew Krebs^1^, Donna Graham^1^, Fiona Thistlethwaite^1^, Louise Carter^1^, Natalie Cook^1^

##### ^1^The Christie NHS Foundation Trust, Manchester, UK, ^2^University of Manchester, Manchester, UK, ^3^Cancer Research UK, London, UK

**Background:** Older patients (pts) make up two thirds of cancer pts but only one third of the cancer trial population. It is unknown whether older pts have poorer outcomes in early phase clinical trials (EPCTs). The aim of this research was to understand if EPCTs at a large specialist cancer centre represented older pts (aged ≥65 years (yrs)) and to understand their clinical outcomes compared to younger (<65yrs) pts.

**Method:** Retrospective data analysis was undertaken for new EPCT pts seen at the Christie NHS Foundation Trust (period covered 1^st^ January 2018 to 31^st^ December 2018). Demographic data was collected, including toxicity and response data from those that received an investigational medicinal product (IMP). Statistical analysis was conducted using cross-tabulation and Fisher Exact Test.

**Results:** A total of 436 pts were seen, including 130 pts ≥65yrs (30%). A random selection of 131 pts <65yrs were assessed as controls. In the older pt group, 101/130 (78%) were deemed to be eligible for a phase 1 clinical trial and 29/101 (29%) were subsequently enrolled on to a trial with an IMP. In the <65yr group 97/131 (74%) of pts were deemed suitable and 21/97 (22%) subsequently went on to an IMP trial. There were no significant differences between these groups (p-value 0.663). Despite older pts having significantly more co-morbidity than younger pts (p-value = 0.0127), they were no more likely to suffer ≥ Grade 3 toxicity (p-value = 0.170), require dose reduction ((p-value = 0.671) or drop out of study than the control group (p-value = 0.982). There was no difference in response to treatment between the two groups (p-value 0.762).

**Conclusion:** Despite there being a significant difference in comorbidities between older and younger pts, we did not find any significant differences between pt outcomes or bias of pt selection within our EPCTs.

**Disclosure:** Funded by Experimental Cancer Medicine Team, The Christie NHS Trust Foundation, Manchester, UK

**Corresponding author:** Jessica Lowe

### 65. A randomised phase III multicentre trial to evaluate the duration of anti-PD1 monoclonal antibody monotherapy in patients with metastatic melanoma (DANTE) – Stage 1 Results

#### Sarah Danson^1^, Ruth Plummer^2^, Christian Ottensmeier^3^, Jane Hook^4^, Helen Marshall^5^, Gurdeep Sagoo^5^, David Meads^5^, Janine Bestall^5^, Galina Velikova^5^, Ferdia Gallagher^6^, Alexandra Smith^5^, Sue Bell^5^, Ellen Mason^5^, Eszter Katona^5^, Simon Rodwell^7^, Pippa Corrie^8^

##### ^1^University of Sheffield, Sheffield, UK, ^2^Newcastle University, Newcastle, UK, ^3^University of Southampton, Southampton, UK, ^4^Leeds Teaching Hospitals NHS Trust, Leeds, UK. ^5^University of Leeds, Leeds, UK, ^6^University of Cambridge, Cambridge, UK. ^7^Melanoma Focus, Cambridge, UK, ^8^Cambridge University Hospitals NHS Foundation Trust, Cambridge, UK

**Background:** Immunotherapy has revolutionised the treatment of melanoma and other tumour types. In contrast to some immune checkpoint inhibitors which are given for a fixed duration of 12 weeks, the anti-PD1 antibodies pembrolizumab and nivolumab are licensed to be given every 2-6 weeks until disease progression, which can extend to several years. This is a significant burden to patients and the NHS. Many responses occur in the first year and can continue even after treatment is stopped. The optimal duration of treatment is a major research priority. We hypothesise that continuing treatment beyond 1 year is unnecessary, exposing patients to the risk of developing immune-related toxicities and incurring considerable costs.

**Method:** DANTE is a randomised multi-stage phase III non-inferiority trial. Patients receiving anti-PD1 monotherapy are registered into DANTE within 1 year of starting treatment. Those who remain progression-free at 1 year are then randomised to either a) stop treatment (with the option to restart anti-PD1 therapy or commence other treatment on progression) or b) continue until disease progression/unacceptable toxicity, or for a minimum of 2 years in the absence of progression/toxicity. Participants are followed up for 4 years. The primary outcome is progression-free survival. Secondary outcomes are quality of life, overall survival, response rate and duration, safety, cost effectiveness and patient acceptance of randomisation.

**Results:** The trial includes 3 interim stages to identify early lack of feasibility of recruitment or efficacy. The trial opened in September 2018 and aims to randomise 1208 participants. The results of the Stage 1 analysis of recruitment rate and patient acceptance of randomisation will be presented.

**Conclusion:** The outcomes of DANTE will be of national and international importance in melanoma and other cancers.

**Acknowledgement:** Relatable work to this abstract has been published previously, see P. Corrie, R. Plummer, C. Ottensmeier, J. Hook, H. Marshall, G. S. Sagoo, J. Bestall, G. Velikova, F. Gallagher, A. Smith, S. Bell, E. Mason, E. Katona, W. Gregory, S. Rodwell, S. Danson, “DANTE: A new randomised trial to evaluate the treatment Duration of ANti‐PD1 monoclonal antibody Treatment in patients with metastatic mElanoma” © 2018 John Wiley & Sons A/S. Published by John Wiley & Sons Ltd. https://onlinelibrary.wiley.com/doi/full/10.1111/pcmr.12738

**Disclosure:** Funded by National Institute for Health Research Health Technology Assessment Programme, London, UK

**Corresponding author:** Sarah Danson

### 66. PanCO: An Open-Label, Single-Arm Pilot Study of Phosphorus-32 Microparticles in Unresectable Locally Advanced Pancreatic Adenocarcinoma with FOLFIRINOX or Gemcitabine + Nab-Paclitaxel Chemotherapies

#### Paul Ross^1^, Natalie Phillips^2^, Zarni Win^2^, Christopher Wadsworth^2^, Thankamma Ajithkumar^3^, Luigi Aloj^3^, Edmund Godfrey^3^, Chinenye Iwuji^4^, Rakesh Ganatra^4^, Sudarshan Kadri^4^, Marion Harris^5^, Daniel Croagh^5^, Morteza Aghmesheh^6^, Adnan Nagrial^7^, Nam Nguyen^8^, Mehrdad Nikfarjam^9^, Alain Hendlisz^10^, Thomas Maher^11^, Anna Kraszewski^11^, Harpreet Wasan^2^

##### ^1^Guy's & St Thomas' NHS Foundation Trust, London, UK, ^2^Imperial College Healthcare NHS Trust, London, UK, ^3^Cambridge University Hospitals NHS Foundation Trust, Cambridge, UK, ^4^University Hospitals of Leicester NHS Trust, Leicester, UK, ^5^Monash Health, Melbourne, Australia, ^6^Southern Medical Day Care Centre, Wollongong, Australia, ^7^Westmead Hospital, Sydney, Australia, ^8^Royal Adelaide Hospital, Adelaide, Australia, ^9^Austin Health, Melbourne, Australia, ^10^Institut Jules Bordet Université Libre de Bruxelles, Brussels, Belgium, ^11^OncoSil Medical Ltd., Sydney, Australia

**Disclosure:** Funded by OncoSil Medical Ltd., Sydney, Australia

**Corresponding author:** Paul Ross

This abstract has been published previously, see http://abstracts.asco.org/239/AbstView_239_251839.html

### 67. BEACON: A Randomised, Phase 3 Study of Encorafenib and Cetuximab +/- Binimetinib vs. Choice of Either Irinotecan or FOLFIRI plus Cetuximab in BRAF V600E–Mutant Metastatic Colorectal Cancer Patients

#### Harpreet Wasan^1^, Tobias Arkenau^2^, Mike Braun^3^, Leslie Samuel^4^, Janet Graham^5^, Scott Kopetz^6^, Axel Grothey^7^, Eric Van Cutsem^8^, Rona Yaeger^9^, Takayuki Yoshino^10^, Jayesh Desai^11^, Fortunato Ciardello^12^, Ashwin Gollerkeri^13^, Kati Maharry^13^, Victor Sandor^13^, Janna Christy-Bittel^13^, Lisa Anderson^13^, Josep Tabernero^14^

##### ^1^Hammersmith Hospital, London, UK, ^2^Sarah Cannon Research Institute, London, UK, ^3^Manchester Academic Health Science Centre and The Christie NHS Foundation Trust, Manchester, UK, ^4^Aberdeen Royal Infirmary, Aberdeen, UK, ^5^Beatson West Of Scotland Cancer Centre, Glasgow, UK, ^6^UT MD Anderson Cancer Center, Houston, US, ^7^West Cancer Center, University of Tennessee, Knoxville, US, ^8^University Hospitals Gasthuisberg Leuven and KU Leuven, Leuven, Belgium, ^9^Memorial Sloan-Kettering Cancer Center, New York, US, ^10^National Cancer Center Hospital East, Kashiwa, Japan, ^11^Royal Melbourne Hospital and Peter MacCallum Cancer Centre, Melbourne, Australia, ^12^University of Campania, Caserta, Italy, ^13^Array BioPharma Inc, Boulder, US, ^14^Vall d’Hebron University Hospital and Vall d’Hebron Institute of Oncology, Barcelona, Spain

**Background:** *BRAF* mutations are identified in up to 15% of metastatic CRC (mCRC) pts and confer poor prognosis. In pts who are refractory to initial therapy, objective response rates (ORR) to standard chemotherapy and biologic combinations are generally <10%, with median progression-free survival (PFS) and overall survival (OS) approximately 2 and 4–6 months (mo), respectively. A 30 pt safety lead-in (SLI) of the BEACON study assessed the safety, tolerability, and efficacy of the combination of encorafenib (ENCO) + binimetinib (BINI) + cetuximab (CETUX) in pts with *BRAF* V600E–mutant mCRC after failure of 1 or 2 prior regimens, demonstrated a confirmed ORR of 48% [95% CI: 29.4, 67.5], including complete responses (CR) in 3 pts, median PFS of 8.0 mo [95% CI: 5.9, 9.3], and mature median OS of 15.3 mo [95% CI: 9.6, NR]. The combination was generally well tolerated, confirming the dose selection of the triplet combination for the randomized portion of the study.

**Method:** The BEACON Study (NCT02928224) is a multicenter, randomized, open-label, 3-arm, phase 3 study to evaluate ENCO+CETUX with/without BINI (vs. investigator’s choice of irinotecan or FOLFIRI + CETUX (control) in pts with *BRAF* V600E‒mutant mCRC whose disease has progressed after 1 or 2 prior regimens in the metastatic setting. 665 pts were randomized. The primary endpoints are OS and ORR (blinded central review) comparing the triplet to the control arm; secondary endpoints include PFS, duration of response, time to response, and comparisons of the doublet to the triplet and control arms.

**Results:** Results of the initial analysis, including efficacy and safety, will be reported.

**Conclusion:** In the SLI, the combination of ENCO+BINI+CETUX showed encouraging activity in pts with *BRAF* V600E‒mutant mCRC. This regimen may be a new standard of care if these results are confirmed in the randomized portion of the study.

**Acknowledgement:** A version of this abstract has been published previously, see S Kopetz, A Grothey, E Van Cutsem, R Yaeger, H Wasan, T Yoshino, J Desai, F Ciardiello, A Gollerkeri, K Maharry, F Loupakis, Y Hong, N Steeghs, T Guren, H Arkenau, P García Alfonso, V Sandor, J Christy-Bittel, L Anderson, J Tabernero, “LBA-006 - BEACON CRC: a randomized, 3-Arm, phase 3 study of encorafenib and cetuximab with or without binimetinib vs. choice of either irinotecan or FOLFIRI plus cetuximab in BRAF V600E–mutant metastatic colorectal cancer”, *Annals of Oncology*, Volume 30, Issue Supplement_4, July 2019, mdz183.004, by permission of Oxford University Press. https://academic.oup.com/annonc/article/30/Supplement_4/mdz183.004/5526665

**Disclosure:** Funded by Array BioPharma, Boulder, US. was the Sponsor. Pierre Fabre Medicament, Paris, France, Merck KGaA, Darmstadt, Germany, Ono Pharmaceutical Co. Ltd. Osaka, Japan were ‘Collaborators'

**Corresponding author:** Harpreet Wasan

### 68. Anti-oestrogen medication use in breast cancer patients and subsequent risk of gastrointestinal cancers: a two country pooled analysis

#### Úna McMenamin^1^, Harlinde De Schutter^2^, Michael B. Cook^3^, Stuart McIntosh^4^, Brian T Johnston^4^, Chris Cardwell^1^

##### ^1^Queen’s University Belfast, Belfast, UK, ^2^Belgian Cancer Registry, Saint-Josse-ten-Noode, Belgium, ^3^National Cancer Institute, Bethesda, US, ^4^Belfast Health & Social Care Trust, Belfast, UK

**Background:** There is a strong male predominance in the incidence of oesophageal and stomach cancer, and to a lesser extent, colorectal cancer, suggesting that oestrogens may be protective against the development of these cancers. In a pooled analysis of two large European breast cancer cohorts, we aimed to evaluate associations between anti-oestrogen medication use and risk of upper GI (oesophageal or stomach) and colorectal cancer.

**Method:** Breast cancer patients were identified from the Belgian Cancer Registry (2004-2014) and the Scottish Cancer Registry (2009-2017). Linkages to national health insurance records (Belgium) and the Prescribing Information System (Scotland) provided detailed information on anti-oestrogen medication use, including tamoxifen and aromatase inhibitors. The primary outcome was GI cancer (UGI or colorectal). Time-dependent Cox regression was used to calculate hazard ratios (HRs) and 95% confidence intervals (CIs) for hormone therapy use and GI cancer risk, adjusting for potential confounders. Fixed effects meta-analysis was used to pool results between the two cohorts.

**Results:** A total of 113,771 breast cancer patients (Belgium: 87,166, Scotland: 26,605), were included. During follow-up, 701 GI cancers occurred including 151 UGI and 550 colorectal. Overall, no association was shown for anti-oestrogen medication use and risk of GI cancer in pooled analysis (adjusted HR: 0.92, 95% CI 0.74, 1.15). In analysis by cancer site, findings were similar for colorectal cancer (pooled adjusted HR: 0.84, 95% CI 0.66, 1.07) and although HRs were raised for UGI cancer, results were non-significant (pooled adjusted HR: 1.47, 95% CI 0.85, 2.53). Similar results were obtained in dose-response analysis.

**Conclusion:** In a two-country study of breast cancer patients, we found little evidence of an association between anti-oestrogen medications and risk of GI cancers of the UGI or colorectum. Given the relatively small number of UGI cancer cases, further studies, preferably through pooled analyses of multiple cohorts, are needed to validate our findings.

**Disclosure:** Funded by Cancer Research UK, London, UK

**Corresponding author:** Úna McMenamin

### 69. TOPARP-B: A Phase II Randomised Trial of the Poly(ADP)-Ribose Polymerase (PARP) Inhibitor Olaparib for Metastatic Castration Resistant Prostate Cancers (mCRPC) with DNA Damage Repair (DDR) Alterations

#### Joaquin Mateo^1^, Nuria Porta^2^, Ursula McGovern^3^, Tony Elliott^4^, Robert Jones^5^, Isabel Syndikus^6^, Christy Ralph^7^, Suneil Jain^8^, Mohini Varughese^9^, Omi Parikh^10^, Simon Crabb^11^, Angus Robinson^12^, Duncan McLaren^13^, Alison Birtle^14^, Jacob Tanguay^15^, Susana Miranda^2^, George Seed^2^, Claudia Bertan^2^, Aude Espinasse^2^, Peter Chatfield^2^, Diletta Bianchini^16^, Emma Hall^2^, Suzanne Carreira^2^, Johann De Bono^17^

##### ^1^Vall d’Hebron Institute of Oncology, Barcelona, Spain, ^2^The Institute of Cancer Research, London, UK, ^3^University College Hospital, London, UK, ^4^The Christie Hospital, Manchester, UK, ^5^The Beatson West of Scotland Cancer Centre, Glasgow, UK, ^6^The Clatterbridge Cancer Centre, Birkenheadl, UK, ^7^St James's University Hospital, Leeds, UK, ^8^Belfast City Hospital, Belfast, UK, ^9^Musgrove Park Hospital, Taunton, UK, ^10^Royal Blackburn Hospital, Blackburn, UK, ^11^Southampton General Hospital, Southampton, UK, ^12^Royal Sussex County Hospital, Brighton, UK, ^13^Western General Hospital, Edinburgh, UK, ^14^Royal Lancaster Infirmary, Lancaster, UK, ^15^Velindre Cancer Centre, Cardiff, UK, ^16^The Royal Marsden NHS Foundation Trust, London, UK, ^17^The Institute of Cancer Research, London, UK,& The Royal Marsden NHS Foundation Trust, London, UK

**Disclosure:** Funded by AstraZeneca UK Ltd, Luton, UK, Cancer Research UK, London, UK

**Corresponding author:** Johann De Bono

This abstract has been published previously, see https://abstracts.asco.org/239/AbstView_239_254043.html for original and CC-BY license.

### 70. Cachexia-related biomarkers predict shortened survival and treatment-related adverse outcomes in a population receiving palliative chemotherapy for lung cancer

#### Joanna Bowden^1,2,3^, Linda Williams^2^, Amanda Swan^3^, Richard Skipworth^4^, Marie Fallon^2^

##### ^1^University of St Andrews, St Andrews, UK, ^2^University of Edinburgh, Edinburgh, UK, ^3^NHS Fife, Kirkcaldy, UK, ^4^NHS Lothian, University of Edinburgh, Edinburgh, UK

**Background:** Optimal patient selection for palliative chemotherapy for lung cancer poses significant challenges. Several factors are associated with adverse outcomes in lung cancer, including poor performance status (measured by Eastern Cooperative Oncology Group, ECOG PS), low body mass index (BMI), weight loss and systemic inflammation. We sought to identify predictive variables for a range of adverse outcomes in a population receiving palliative chemotherapy for lung cancer.

**Method:** A retrospective cohort study of patients who received first-line palliative chemotherapy for lung cancer during 2013-2015 in South East Scotland was undertaken. Demographic and clinical data was extracted from electronic health records. Body composition analysis was conducted using diagnostic computed tomography scans to evaluate muscularity (skeletal muscle index, SMI) and muscle density (muscle attenuation, MA). Established thresholds for variables were utilised where available. Where not available, optimal stratification was used to derive discriminatory thresholds for overall survival (OS). Outcome measures included OS and treatment-related adverse events. Only OS is reported here. Time to event data were analysed using Kaplan-Meier methods and Cox Proportional Hazards regression.

**Results:** 397 patients were included; 259 with non-small cell lung cancer (NSCLC) and 138 small cell lung cancer (SCLC). 295 (80%) had an ECOG PS of 0/1 at diagnosis. Mean BMI was 25.9 (SD=5.3) Median OS was 215 days (95%CI 191, 239). 191 patients (48%) received fewer than 4 cycles of chemotherapy; their median survival was 112 days (95%CI 97-127), p<0.001. Independent predictors of reduced OS were baseline NLR ≥4, Albumin <35, SMI and MA. ECOG PS was not a significant predictor of OS. 4 composite models based on independent predictors were explored.

**Conclusion:** It is possible to identify patients at significant risk of reduced OS and other adverse outcomes at diagnosis. Our predictive models require further validation and could improve patient selection for palliative chemotherapy for lung cancer in the future.

**Corresponding author:** Joanna Bowden

## Living with and beyond cancer

### 71. The impact of type 2 diabetes on survival in patients with cancer independent of its effect on survival in individuals without cancer: a matched cohort analysis

#### Andrew Renehan^1^, Nasra Alam^1^, Alison Weight^1^, Martin Rutter^1^, Matthew Sperrin^1^, Darren Ashcroft^1^, Andrew Renehan^1^

##### ^1^University of Manchester, Manchester, UK

**Background:** Patients with cancer and type 2 diabetes (T2D) have a poorer survival than those without T2D. However, studies fail to account for the adverse prognostic effect of T2D on mortality in individuals without cancer. This study aimed to determine whether individuals with cancer and T2D have reduced survival over and above what would be expected by cancer and T2D acting separately i.e. a cancer-T2D interaction.

**Method:** A matched cohort study was performed in the Clinical Practice Research Datalink (England), linked with national cancer statistics (1998-2015), to derive cohorts with incident versus never cancer, aged 30 to 85 years. We then derived four groups: cancer never T2D (148,467); cancer prevalent T2D (14,124); never cancer never T2D (688,730); never cancer prevalent T2D (57,577). The primary outcome was 10-year overall survival (OS). Results from Cox models with cancer-T2D interactions were expressed as hazard ratios (HRs) and 95% confidence intervals (CIs).

**Results:** In men, the 10-year OS were: cancer never T2D, 32%; cancer prevalent T2D, 28%; never cancer never T2D 68%; never cancer prevalent T2D, 57%. In women, the respective 10-year OS were: 45%, 36%, 78%, 62%. In the interaction model for men, the HR (prevalent T2D versus never T2D) was 1.16 (95% CI: 1.13-1.19); interaction term: 0.99 (95% CI: 0.95-1.04). For women, the HR (prevalent T2D versus never T2D) was 1.29 (95% CI: 1.24-1.33); interaction term: 0.78 (95% CI: 0.74-0.82, p < 0.001).

**Conclusion:** Patients with cancer and T2D had a poorer survival compared with those without T2D. For men, the adverse effect of T2D reflected the adverse prognostic impact of T2D in the non-cancer population; for women, there was evidence that the adverse impact of T2D was lessened among patients with cancer. Understanding these observations through further research will help inform strategies to reduce mortality in patients with cancer and T2D.

**Disclosure:** Funded by Cancer Research UK, London, UK

**Corresponding author:** Andrew Renehan

### 72. Hormone-related diseases and prostate cancer: an English national record linkage study

#### Eleanor Watts^1^, Timothy Key^1^, Ruth Travis^1^, Aurora Perez-Cornago^1^, Raphael Goldacre^1^, Naomi Allen^1^

##### ^1^University of Oxford, Oxford, UK

**Background:** There is evidence that circulating insulin-like growth factor (IGF-I) and testosterone concentrations are related to prostate cancer risk. Acromegaly is associated with clinically high IGF-I concentrations, while Klinefelter’s syndrome, testicular hypofunction and hypopituitarism are associated with clinically low testosterone concentrations. We aimed to investigate whether diagnoses with these conditions are associated with subsequent prostate cancer diagnosis and mortality.

**Method:** Linked English national Hospital Episode Statistics and mortality data from 1999 to 2017 were used to construct and follow-up cohorts of men aged ≥35 years diagnosed with i) acromegaly (n=2,495) and ii) hypogonadal-associated diseases (n=18,763): Klinefelter’s syndrome (n=1,992), testicular hypofunction (n=8,086) and hypopituitarism (n=10,331). We estimated adjusted hazard ratios (HRs) and confidence intervals (CIs) for subsequent prostate cancer diagnosis and death using Cox regression in comparison with an unexposed reference cohort of 4.3 million men, who were admitted to hospital for a wide range of relatively minor surgeries and conditions, among whom there were nearly 130,000 observed prostate cancer diagnoses and 30,000 prostate cancer deaths.

**Results:** For men diagnosed with acromegaly, the subsequent HR for prostate cancer was 1.33 (95% CI 1.09-1.63; P=0.005; n observed cases=96) and the HR for prostate cancer death was 1.44 (95% CI 0.92-2.26; P=0.11; n deaths=19). Diagnosis with Klinefelter’s syndrome was associated with a lower prostate cancer risk (HR=0.58, 95% CI 0.37-0.91; P=0.02; n observed cases=19) and hypopituitarism was associated with a reduced risk of prostate cancer death (HR=0.57, 95% CI 0.42-0.79; P=0.001; n deaths=23).

**Conclusion:** Our results support the hypothesised role of IGF-I and testosterone in prostate cancer development and/or progression. These findings are important because they provide insight into prostate cancer etiology.

**Disclosure:** Funded by Cancer Research UK, London, UK

**Corresponding author:** Eleanor Watts

### 73. The Trigger project: Introducing electronic patient reported outcome measures into radiotherapy services

#### Amy Sharkey^1^, Archie MacNair^1^, Kerlann Le Calvez^2^, Robert Walters^3^, Lesley Smith^4^, David Bloomfield^5^, Annmarie Nelson^6^, John Staffurth^7^, Matthew Williams^2^, Jane Maher^8^

##### ^1^Macmillan Cancer Support, London, UK & Royal College of Radiologists, London, UK, ^2^Imperial College Healthcare NHS Trust, London, UK, ^3^Pelvic Radiation Disease Association, Epsom, UK, ^4^NHS England National Cancer Programme, London, UK ^5^Brighton and Sussex University Hospitals NHS Trust, Brighton, UK, ^6^Marie Curie Research Centre, London, UK, ^7^Velindre NHS Trust, Cardiff, UK, ^8^Macmillan Cancer Support, London, UK

**Background:** Patients receiving pelvic radiotherapy can experience long term gastrointestinal side effects post-radiotherapy. The Trigger project identifies patients experiencing symptoms of radiation-related bowel toxicity using the ALERT-B questionnaire, and directs them to the appropriate clinician. Trigger is a service evaluation project, aiming to prove the utility of electronic patient reported outcome measures (PROMs), and to demonstrate the feasibility of a low-resource project as a model for collecting PROMs. It is a collaboration between Macmillan Cancer Support, the Royal College of Radiologists, and three NHS Trusts: Velindre, Imperial College Healthcare and Brighton and Sussex University Hospitals.

**Method:** Patients register on the Trigger website, hosted by My Clinical Outcomes, and receive periodic emails to complete the short ALERT-B questionnaire electronically, to screen for long-term bowel symptoms which could have been caused by pelvic radiotherapy. If answering “yes” to any of the questions, patients are directed to appropriate services. Six months following the completion of their radiotherapy, patients are sent a separate questionnaire to evaluate the utility of the project.

**Results:** 336 patients registered in first the 9 months across the 3 sites. Patients with a range of different cancers signed up: anal (2%), bladder (1%), prostate (87%), rectal (4%) and gynaecological (6%). 43 patients (of 65 eligible (uptake 66%)) have answered their 6-month post treatment questionnaire thus far, and 72% answered "yes" to at least one of the ALERT-B questions. 85% of responding patients reported they found the Trigger project helpful.

**Conclusion:** These promising results show that electronic PROMS can be introduced in radiotherapy departments using a low resource model. The Trigger project works as a feasibility model, showing patients engage with electronic PROMs projects, and find them useful. PROMs for other tumour types could be collected in a similar manner, based on the low-resource model used here, using site-specific PROMs based on the ALERT-B tool.

**Disclosure:** Funded by Macmillan Cancer Support, London, UK

**Corresponding author:** Amy Sharkey

### 74. Barriers to accessing cancer services in England and Wales by adults with physical disabilities

#### Dikaios Sakellariou^1^, Sally Anstey^1^, Molly Courtenay^1^, Daniel Kelly^1^, Sarah Polack^2^, Eleri Girt^3^, Wendy Wilkinson^3^

##### ^1^Cardiff University, Cardiff, UK ^2^London School of Hygiene and Tropical Medicine, London, UK, ^3^Wales Cancer Network, Cardiff, UK

**Disclosure:** Funded by Tenovus Cancer Care, Cardiff, UK

**Corresponding author:** Dikaios Sakellariou

This abstract has been published previously, see https://bmjopen.bmj.com/content/9/6/e027555

### 75. Cancer Quality of Life Metric Project – Lessons learned from an implementation pilot

#### Alice Simon^1^, Sarah Gelcich^2^, Adam Glaser^2^, Andria Hanbury^3^, Luke Hounsome^4^, Jane Maher^5^, Erik Mayer^6^, Alison Richardson^7^, Simon Rogers^8^, Lesley Smith^1^, Galina Velikova^2^, on behalf of NHS England Cancer Quality of Life Working and Steering Groups^1^

##### ^1^NHS England, London, UK, ^2^University of Leeds, Leeds, UK, ^3^York Health Economics Consortium, York, UK, ^4^Public Health England London, UK, ^5^Macmillan Cancer Support, London, UK, Mount Vernon Cancer Centre, Northwood, UK ^6^Imperial College Healthcare NHS Trust, London, UK, The Royal Marsden NHS Foundation Trust, London, UK, ^7^University of Southampton and University Hospital Southampton NHS Foundation Trust, Southampton, UK ^8^Aintree University Hospital NHS Foundation Trust, Liverpool, UK

**Background:** Quality of life (QoL) outcomes are important to patients. The NHS long term plan recognises this issue. A feasibility and acceptability pilot in cancer patients is testing data collection and production of QoL metric scores to support patient, regional and national level monitoring.

**Method:** Five Cancer Alliances (in seven hospital trusts) are testing data collection processes. Breast, prostate and colorectal cancer patients complete two questionnaires (EQ-5D; EORTC QLQ-C30). Patients are invited to the survey using an electronic platform. Non-responders are reminded with an option to use a paper questionnaire. A subset of patients is asked to repeat the survey 6-months later. Responses are linked to demographic, disease and treatment data held by the National Cancer Registration and Analysis Service. Analyses are testing appropriateness of different summary or sub-scores for benchmarking QoL outcomes. A small-scale test providing individual-level feedback to patients and clinicians is included. A process evaluation includes qualitative interviews and focus groups with administrators, patients and clinicians, plus quantitative monitoring of coverage and uptake.

**Results:** To date, N=3441 patients have been invited to participate with N=1758 (51%) completing questionnaires. N=1003 (57%) completed electronically and N=755 (43%) by paper. Qualitative evaluations highlighted challenges in identifying, inviting and reporting on cancer patients. Patients supported the use of the two questionnaires for metric monitoring, but also recommended inclusion of cancer-specific questionnaires. Patients prefer a traffic-light visual summary of their data rather than figures alone.

**Conclusion:** Both electronic and paper options for completion are necessary. Data collection systems must have as little impact on the delivery of care as possible. Summary data should integrate into care pathways to facilitate support that underpins improvements in patient outcomes. Through the collection of national data and appropriate case-mix adjustments, it will be possible to give clear expectations of outcomes for patients with different tumour sites and clinical characteristics.

**Disclosure:** Funded by NHS England, London, UK

**Corresponding author:** Simon Rogers

### 76. The placebo response in trials of drug treatments for cancer-related fatigue: A systematic review, meta-analysis and meta-regression

#### Maria del Rocio Roji Buqueras^1^, Patrick Stone^2^, Federico Ricciardi^2^, Bridget Candy^2^

##### ^1^University College London, London, UK, ^2^The Marie Curie Palliative Care Research Department, University College London, London, UK

**Background:** Cancer related fatigue (CRF) is one of the most distressing symptoms experienced by cancer patients. There is no gold standard treatment, although multiple drugs have been tested with little evidence of efficacy. Many randomized clinical trials (RCTs) reports in this area commented on the existence of a placebo response (PR). The objective of this systematic review was to establish the magnitude of the PR in RCTs of drugs to relieve CRF and to identify contributing factors.

**Method:** We conducted a systematic review of RCTs comparing drug against placebo, in which the main objective was to treat CRF, in adult cancer patients, at any disease stage, where fatigue was measured using a validated tool. We conducted a meta-analysis using the standardized mean change (SMC) between baseline and final measurement in the placebo group. We undertook a meta-regression to explore factors that may have been associated with the PR (e.g. patient population or trial design). We assessed the risk of bias for each trial using the criteria outlined in the Revised Cochrane tool.

**Results:** We identified 3916 publications of which 23 were included in quantitative analysis. Studies had limitations, which increased potential risk of bias. The pooled SMC estimate was -0.23 (95% CI -0.42 to -0.04). This is a moderate effect size that was statistically significant (p = 0.02). None of the multiple variables analysed in the meta-regression were statistically significantly related to PR.

**Conclusion:** There is some evidence, based on trials with small samples, that the PR in trials testing drugs for cancer related fatigue is moderate but statistically significant. No factors related to PR were identified. Since the PR is relatively large, we recommend that, in uncontrolled studies, potential treatments for CRF should be able to demonstrate an effect size of at least 0.23 before being considered for further evaluation in RCTs.

**Disclosure:** Funded by the Marie Curie I-CAN-CARE Programme grant (MCCC-FPO-16-U), London, UK. Professor Stone is supported by the Marie Curie Chair's grant (MCCC-509537), London, UK.

**Corresponding author:** Maria del Rocio Roji Buqueras

### 77. Is it possible to deliver an effective intervention in tackling fears of cancer recurrence?

#### Conor Wheeler^1^

##### ^1^University of St. Andrew's, St. Andrew's, UK

**Background:** Fear of cancer recurrence (FCR) is the most commonly reported concern post-treatment in the ever growing number of cancer survivors. It is estimated that over 70% of cancer survivors suffer some form of FCR, with high levels linked to anxiety, depression and reduced quality of life. There are screening tools for FCR, however, once identified there is little consensus on how best to manage it. Current interventions adopt a range of approaches, with varying benefit. This review aims to determine which aspects of an FCR intervention appear most effective and considers how these can be integrated into healthcare.

**Method:** This systematic review employed a literature search using Ovid MEDLINE, PubMed and Web of Science databases. Inclusion and exclusion criteria were defined to identify studies evaluating interventions to reduce FCR. Once selected, details of intervention method, control arm, patient population, and outcomes were recorded, also duration of intervention and professional delivering it. Each study was assessed with Cochrane risk of bias tool. Quality of evidence was evaluated using GRADE.

**Results:** Eleven randomised controlled trials (n=2060 participants) were selected for inclusion in the review. 4 trials used face-to-face interventions, 3 online, 2 a combination of both, one telephone consultations and one assessed patient directed follow-up. Most effective in reducing FCR was a blend of online and face-to-face support, followed by group face-to-face therapies. Both mindfulness-based therapies and behavioural modification strategies reported positive effects. Online tools alone were not effective, and patient directed follow-up was worse than standard follow-up. The specific professional delivering intervention and their relationship with the patient appeared important.

**Conclusion:** It is possible to reduce FCR with psychological and support interventions. Group face to face therapy complemented by online resources appears most effective. The challenge ahead is how we deliver this effectively, economically and sustainably to improve quality of life in cancer survivors.

**Corresponding author:** Conor Wheeler

### 78. A reference guide for patient and public involvement contributors. How the ECMC Network PPI group developed a resource for patients and the public

#### Nikki Hayward^1^, Ruth Boyd^2^, Helen Bulbeck^3^, Clare Dickinson^4^, Jayne Doran^5^, Karen Turner^6^, Elspeth Banks^7^, Kate Cleary^8^, Laura Rooney^9^, Ernesto Rogarto^10^, Hannah Brown^11^

##### ^1^Oxford University Hospitals NHS Trust, Oxford, UK, ^2^Northern Ireland Cancer Trials Centre, Belfast, UK, ^3^Brainstrust, Cowes, UK, ^4^The Christie NHS Foundation Trust, Manchester, UK, ^5^Birmingham Experimental Cancer Medicine Centre (ECMC), Birmingham, UK, ^6^University of Birmingham, Birmingham, UK, ^7^Glasgow Experimental Cancer Medicine Centre (ECMC), Glasgow, UK, ^8^Wales Cancer Research Centre, Cardiff, UK, ^9^University of Glasgow, Glasgow, UK, ^10^Leeds Cancer Trials Centre, Leeds, UK, ^11^Experimental Cancer Medicine Centre (ECMC), Cancer Research UK, London, UK

**Background:** Patient and public involvement is now embedded into many aspects of cancer research, including gaining thoughts and opinions on the earliest design phases, through to the provision of lay perspectives on ethics committees, trial steering committees and service provision.

When the ECMC network patient and public involvement group was originally established, they identified a gap in training for lay people involved in early phase cancer research. To address this gap, the group decided to produce a handbook which would support people affected by cancer who were taking part in PPI activities.

**Method:** The handbook evolved over a period of two years, through face to face meetings of the working group, teleconferences and virtual review of content by the wider group. The handbook was produced by a combination of lay members and professionals. Reviews of content by PPI committees across the UK were sought to provide the widest possible perspective prior to deciding on the final version.

**Results:** To collate a condensed version of many aspects of early phase research, and ensure the information could be understood by lay people was challenging and relied on the input and knowledge of a combination of professionals and people affected by cancer.

Feedback from the wider PPI groups gave us clearer direction for the final version, especially with regards to layout. The results of the combined efforts of the ECMC PPI working group, the handbook will be published later this year.

**Conclusion:** Patients and public who get involved with activities to support cancer research are expected to give feedback on complicated proposals which use scientific language, with no previous background in the topic. Providing a handbook for those involved in PPI activity will aid understanding of the projects being reviewed and enable lay people to give feedback with confidence.

**Disclosure:** Funded by Experimental Cancer Medicine Centre (ECMC), London, UK

**Corresponding author:** Nikki Hayward

### 79. The impact of dietetic input in post radiotherapy head and neck cancer patients

#### Robynne Cranston^1^, Nola Lynch^1^, Isobel Bowe^1^, Charles Kelly^2^, Shahid Iqbal^2^, Rachel A Pearson^2,3^

##### ^1^Nutrition and Dietetics Department, Freeman Hospital, NHS Newcastle upon Tyne Hospitals, Newcastle upon Tyne, UK, ^2^Northern Centre for Cancer Care, Freeman Hospital, NHS Newcastle upon Tyne Hospitals, Newcastle upon Tyne, UK, ^3^Newcastle University, Newcastle upon Tyne, UK

**Background:** The impact of regular dietetic input 8 weeks after radiotherapy treatment for head and neck cancer (H&N) is poorly understood. Nutritional status is an important clinical issue in this phase.

**Method:** This study evaluated the impact of dietetic input into an already established once-weekly post radiotherapy (PRT) clinic. Data was collected from two patient cohorts over an 18 month period: Group 1: pre dietetic input (n= 171); Group 2 dietetic input weekly for 8 weeks (n= 191). Percentage weight loss, duration of oral and enteral nutritional support, hospital admission rates and duration of stay, and duration of community dietetic follow-up were recorded. QOL H&N cancer questionnaires were completed week 1 and 6 of PRT.

**Results:** Dietetic follow-up time PRT was reduced from 49 to ≤7 days PRT (p=0.00). Percentage weight loss (beginning of radiotherapy to week 8 PRT) in Group 2 was 5.7% compared with 8.3% Group 1(p=0.00). Patients requiring oral nutritional support (ONS) reduced high calorie supplement drinks from 3 bottles to 1, compared to 3 bottles to 2 in Group 1 (p=0.00). 41.7% of patients in Group 2 discontinued ONS during the PRT period compared to 7.4% in Group 1 (p=0.00). Patients reported areas of their QOL improved; enjoyed life more; and able to enjoy food with managing more solid textures. There was no statistically significant difference in the number of NG tubes placed or duration of feeding after PRTC. Total hospital admission days over 18 months was reduced by 24 days compared to group 1.

**Conclusion:** Intensive weekly dietetic support in head and neck cancer radiotherapy patients for 8-weeks after treatment can provide important gains in nutritional status with reduced need for ONS and improved QOL. Furthermore, this approach may have cost benefits for NHS trusts.

**Disclosure:** Funded by Charlie Bear Cancer Care Charity, Newcastle upon Tyne, UK

**Corresponding author:** Robynne Cranston

### 80. Dietary changes and nutritional support after a pelvic cancer diagnosis: a cross-sectional study

#### Georgios Saltaouras^1^, Helen Lightowler^2^, Shelly Coe^2^, Amanda Horne^3^, Sara Matthews^3^, Loryn Caulfield^3^, Eila Watson^1^

##### ^1^Oxford Institute of Nursing, Midwifery and Allied Health Research, Oxford Brookes University, Oxford, UK, ^2^Department of Sport, Health Sciences and Social Work, Oxford Brookes University, Oxford, UK, ^3^Department of Oncology, Oxford University Hospitals NHS Foundation Trust, Oxford, UK

**Background:** Diet and nutrition is an important aspect of survivorship care that is not routinely addressed. Patients diagnosed with a cancer in the pelvis (anal, bladder, rectal and cancers of the reproductive organs) may benefit from dietary modifications that could improve treatment outcomes and quality of life, and may influence survival. This study aims to explore pelvic cancer survivors’ dietary habits and experiences of nutritional support after diagnosis.

**Method:** People diagnosed with a pelvic cancer, either undergoing (*n*=266) or having completed radiotherapy treatment 6-24 months previously (*n=*405), were invited to participate in a cross-sectional survey. Descriptive and multivariable logistic regression analyses were undertaken. Results are presented for the whole sample, as there were no significant differences between the two groups.

**Results:** 254 (38%) survivors responded; median age 70 years. High overweight and obesity rates (39% and 24%, respectively) and presence of treatment side effects (e.g. bowel changes, appetite issues, fatigue) (82%) were observed. Two thirds of respondents (*n*=170) reported at least one dietary change since diagnosis. Most notable changes included increased intake of vegetables (33%) and fruit (33%) and reduction of sugary foods (48%) and alcohol (41%). Forty-three percent (n=108) received dietary support from the healthcare team, of which 67% (n=72) felt their needs to be well met. Receipt of support from the healthcare team was the only significant predictor of dietary change (OR 3.63, 95% CI: 1.82-7.23). Dietary support was mainly in the form of leaflets from specialist nurses. Sixty-eight percent (n=171) of respondents would like to receive additional dietary support in relation to cancer.

**Conclusion:** This study shows that survivors make dietary changes following a diagnosis of pelvic cancer and highlights an increased need and interest for nutrition information and support. Further development of support services in the area of nutrition is warranted.

**Disclosure:** Funded by Oxford Brookes University, Oxford, UK

**Corresponding author:** Georgios Saltaouras

### 81. Understanding international variation in access to PET-CT for oncology diagnostics: An International Cancer Benchmarking Partnership (ICBP) study

#### Charlotte Lynch^1^, Irene Reguilon^2^, Deanna Langer^3^, Damon Lane^4^, Wai-Lup Wong^5^, Fergus McKiddie^6^, Andrew Ross^7^, Lorraine Shack^8^, Thida Win^5^, Christopher Marshall^9^, Mona-Elizabeth Revheim^10^, Bolette Danckert^11^, Sabina Dizdarevic^5^, Canice McGivern^12^, Anne Hazlett^12^, Cheryl Louzado^13^, Mark MacMillan^8^, Sam Harrison^1^

##### ^1^Cancer Research UK, London, UK, ^2^eConsult, London, UK, ^3^Cancer Care Ontario, Toronto, Canada, ^4^Pacific Radiology, Wellington, New Zealand, ^5^NHS England, London, UK, ^6^NHS Grampian, Aberdeen, UK, ^7^Canadian Association of Nuclear Medicine, Ottawa, Canada, ^8^Alberta Health Services, Edmonton, Canada, ^9^Wales Research and Diagnostic PETIC, Cardiff, UK, ^10^Oslo University Hospital, Oslo, Norway, ^11^Aarhus University, Aarhus, Denmark, ^12^Belfast Health and Social Care Trust, Belfast, UK, ^13^Canadian Partnership Against Cancer, Toronto, Canada

**Background:** PET-CT is an important diagnostic tool within cancer care, with evidence supporting its specificity, accuracy and sensitivity in detecting tumours, metastatic spread, and treatment monitoring. Variation in access to diagnostics has been identified as a potential contributor to international cancer survival differences. This study is the first to our knowledge exploring the differences between PET-CT guidelines, capacity and issues surrounding effective service delivery.

**Method:** Mixed methods including quantitative data collection on 4 access metrics (capacity, use, cost, location) from existing administrative data in 17 jurisdictions across high-income countries (UK, Ireland, Norway, Denmark, Canada, Australia, New Zealand) from the year 2000 onwards. Capacity was measured by scanner quantity and waiting times; use was measured by the number of scans carried out annually in the general and cancer populations. Literature searches were performed for clinical indication evidence. Descriptive comparative analyses were produced of use, capacity and indication guidance for PET-CT services between jurisdictions.

**Results:** 11/17 jurisdictions were able to provide complete data on scanner location and capacity. Number of PET-CT scanners ranged from 0.05 to 0.66 per 100,000. Acquisition of scanners over time showed the greatest increase in Denmark, 0.02 scanners per 100,000 in 2007, to 0.66 in 2017. Indications ranged between jurisdictions, with potentially clinically important differences seen in recommendations for colorectal cancer staging and within recommendations for non-small cell lung vs small-cell lung cancers. Data access, sources and definitions surrounding PET-CT services varied significantly across ICBP jurisdictions.

**Conclusion:** The lack of international PET-CT data availability and service consistency may act as a barrier in monitoring and implementing effective PET-CT services internationally. There is an unmet need in capturing more consistent, richer data relating for PET-CT activity to facilitate sharing of best practice and improve future planning for healthcare provision. Availability of PET-CT should be considered as a proxy for investment in, and quality of cancer care.

**Disclosure:** Funded by Cancer Research UK, London, UK

**Corresponding author:** Charlotte Lynch

### 82. A single centre review of the NICE approved, standard of care, treatment cost savings for patients with metastatic castrate-resistant prostate cancer (mCRPC) enrolled in clinical trials

#### Louisa McDonald^1^, Nicholas Gomm^1^, Anita Soma^1^

##### ^1^Guy's and St Thomas' NHS Foundation Trust, London, UK

**Background:** When a patient is enrolled in a clinical trial, often the study sponsor will provide the standard of care treatment without charge to the study site, either as the comparator drug, to be used in combination with the investigational product or in the form of an extension study post licencing. A treatment cost saving can be seen based on the standard of care treatment the patient would have received.

For patients with metastatic castrate-resistant prostate cancer, the current NICE guidelines recommend the use of either Abiraterone (Zytiga) in combination with prednisone or prednisolone or Enzalutamide (Xtandi). The NICE approved treatment costs are £2,300 and £2,7934.67 respectively.

**Method:** The objective of the review was to analyse the treatment saving based on the standard of care treatment cost. The review looked at 5 industry sponsored studies at Guy’s and St Thomas’ (GSTT) between April 2015 and March 2018. The studies reviewed were for men with metastatic castrate-resistant prostate cancer (mCRPC) and all included either Abiraterone or Enzalutamide as a comparator, an open label extension study or as a cross over treatment.

The cost saving was calculated based on the number of patients randomised at GSTT, the length of time they were on study and the cost of the standard of care drug they would have received off trial, estimated using the NICE approved costings.

**Results:** The total treatment costs saved across 3 years was an estimated £1.4m, with an average per patient cost saving of approximately £25k per year.

**Conclusion:** Significant treatment cost savings were seen where patients were recruited onto clinical trials. In addition to the benefits to patients and future treatments, the economic impact of clinical trials on NHS trusts is vast. It is predicted that such savings will continue to increase with the licensing of more costly treatments such as immunotherapies.

**Corresponding author:** Louisa McDonald

### 83. Patient Perspective of Experimental Cancer Medicine (PpExCaM Survey) – a Patient-led Initiative to Understand the Views of Participating in Early-Phase Trials

#### Mekala Gunaratnam^1^, Emma Hainsworth^1^, Terry Emmery^2^, Tim Meyer^1^

##### ^1^University College London Cancer Institute, London, UK, ^2^Patient participant

**Background:** We set out to co-produce a project with patients to explore key issues relating to participation in early-phase cancer trials. The purpose was to meaningfully involve patients in all stages including the initial identification of areas of enquiry, survey creation and analysis of the findings. Areas of focus included attitudes towards access to genomics data within trials, data security and perceptions around the risks and benefits of participation.

**Method:** A panel of five patient representatives met on three occasions. Training on concepts relating to early-phase trials was provided in the first meeting and follow-up discussion was facilitated. The survey was distributed amongst a cohort of patients recruited to early-phase trials at our site. Panel members interpreted the survey results.

**Results:** 60 copies of the survey were circulated with 48 completed and returned. 88% of respondents completely agreed with the collection of genomics data as part of a trial and 70% wanted just an overview or no information at all about how it would be used. The pattern of response regarding routinely collected data was similarly positive. All respondents stated that trial information they had been given was either very easy to understand or fairly easy to understand, with no negative responses provided. 85% reported having enough time to make a decision. Motivations for participation were divided between altruism (72%), additional monitoring (48%), and access to new treatments (93%).

**Conclusion:** Patients’ attitudes towards providing data and participating in early phase trials were strongly positive, providing reassurance that information was pitched correctly by the study teams. The response rate to the survey was high suggesting the added value of this level of patient involvement.

**Disclosure:** Funded by University College London Hospitals Biomedical Research Centre (BRC), London, UK

**Corresponding author:** Mekala Gunaratnam

### 84. The landscape of hepatocellular carcinoma in the UK in the past 20 years: the HCCUK/NCRAS partnership

#### Anya Burton^1^, Robert Driver^2^, Vinay Kumar^3^, Katherine Cullen^4^, Rhys Pockett^4^, Deborah Fitzsimmons^4^, Graeme Alexander^5^, Tim Cross^6^, Ian Rowe^7^, Aileen Marshall^8^

##### ^1^Public Health England, London, UK, ^2^University of Leeds, Leeds, UK, ^3^University of Liverpool, Liverpool, UK, ^4^Swansea Centre for Health Economics, Swansea, UK, ^5^University College London, London, UK, ^6^Royal Liverpool and Broadgreen Hospitals, Liverpool, UK, ^7^St James University Hospital Leeds, Leeds, UK, ^8^Royal Free Hospital, London, UK

**Background:** The HCC-UK/NCRAS partnership was created in 2017 to facilitate a wide programme of research relating to hepatocellular carcinoma (HCC) using data available within the National Cancer Registration and Analysis Service (NCRAS). NCRAS data includes tumour- and patient-specific variables, diagnosis and treatment information. These individual-level data are linked to multiple datasets including Hospital Episodes Statistics (HES). Aim: The partnership programme is examining the epidemiology of HCC in England, including regional variation in incidence, routes to diagnosis, treatment and survival, as well as the economic burden.

**Method:** HCC cases were identified using ICD10-O-2 code C22.0 and morphology code M8170. Demographic characteristics were explored and European age-standardised incidence and mortality rates per 100,000 person years calculated. Linked HES codes were used to identify the presence and severity of cirrhosis.

**Results:** 62,135 primary liver cancer cases were diagnosed in England between 1997 and 2016. 29,906 of these were HCC. For HCC the mean age at diagnosis was 68.4 years and the male to female ratio was 3.4. Overall 25% of all HCC cases were from the most deprived population quintile. 58% of HCC cases were identified as having cirrhosis and, of these, 42% had decompensated cirrhosis, through linked HES data. The majority of HCC patients did not receive specific anticancer treatment. The two most common Routes to Diagnosis were emergency presentation (35%) and GP referral (30%).

**Conclusion:** HCC incidence and mortality have tripled over the last 20 years; the most deprived individuals are most at risk. HCC is often associated with cirrhosis and more than one in five individuals diagnosed with HCC has advanced cirrhosis such that treatment options for HCC are severely limited. These trends highlight the urgent need to address prevention strategies for both liver disease and hepatocellular carcinoma specifically at regional and population level.

**Disclosure:** Funded by British Association for the study of Liver Disease, Lichfield, UK, funded by unrestricted educational grant from British Technology Group Ltd

**Corresponding author:** Aileen Marshall

### 85. Patients’ experience of nutritional care during cancer treatment

#### Lesley Turner^1^, Arabella Hayter^1^, Fiona Davey^1^, Stephen Wootton^2^

##### ^1^NIHR Cancer and Nutrition Collaboration, London, UK ^2^University of Southampton, Southampton, UK

**Background:** Good nutrition is integral to the prevention of cancer, as well as to the treatment of the disease and end of life care. The NIHR Cancer & Nutrition Collaboration was set up in 2014 to build and maintain a community of practice of researchers, clinicians and patients. The group presented to the Consumer Forum Dragon’s Den at the 2014 NCRI Conference, who suggested conducting a patient survey to gain insight into nutritional care during cancer treatment.

**Method:** A survey was conducted between January-February 2015 comprising 43 questions, to find out: 1) whether patients receive consistent, evidence-based advice; 2) what other nutritional support, advice and care patients would like to receive; and 3) the major gaps in service provision at diagnosis, during and after treatment. The survey was circulated via the Independent Cancer Patients’ Voice and the NCRI Consumer Forum. Results were analysed by the NIHR Cancer and Nutrition Collaboration.

**Results:** Of 96 responses, 72% were female and most participants were aged between 60-69 years (33%) and 50-59 years (29%). Most patients (n=69, 72%) reported receiving no nutritional advice from their healthcare team, either because they were not offered it (76%) or the patients did not know nutritional advice existed (10%). The most commonly reported problems were changes in taste and smell (70%), appetite loss (69%), nausea and vomiting (56%), being unsure of what to eat (56%) and the inability to be physically active (56%).

**Conclusion:** Many patients reported unsatisfactory experiences of nutritional care in relation to cancer. Gaps identified by patients include how to deal with side-effects of chemotherapy, weight changes and specific foods and diets that patients should or should not consume. There is a need for better evidence to allow more reliable and consistent nutritional and dietetic information for those living with and beyond cancer.

**Acknowledgement:** A version of this abstract has been published previously, see http://cancerandnutrition.nihr.ac.uk/wp-content/uploads/2015/10/CancerNutrition-Summary-Report-28.10.15.pdf for original Copyright © 2015 University of Southampton & University Hospital Southampton NHS Foundation Trust.

**Disclosure:** Funded by NIHR Cancer and Nutrition Collaboration, London, UK

**Corresponding author:** Lesley Turner

### 86. Effective clinical cancer treatment, care and management for people with comorbid cancer and dementia: understanding population demographics and intervention priorities and outcomes (CanDem-Int)

#### Michelle Collinson^1^, Ellen Mason^1^, Laura Ashley^2^, Amanda Farrin^1^, Alys Griffiths^3^, June Hennell^4^, Rachael Kelley^3^, Claire Surr^3^

##### ^1^Clinical Trials Research Unit, University of Leeds, Leeds, UK, ^2^School of Social Sciences, Leeds Beckett University, Leeds, UK, ^3^Centre for Dementia Research, Leeds Beckett University, Leeds, UK. ^4^Centre for Dementia Research Expert by Experience group, Leeds Beckett University, Leeds, UK

**Background:** Dementia and cancer are both common among older people, making it likely that many people will have both conditions. People living with dementia and cancer could have complex care needs, making cancer care provision more difficult. However, there is little research in this area to inform practice.

**Method:** We undertook two studies. Study 1 analysed a large dataset from UK GP records. We identified the numbers of people with cancer and dementia, their characteristics, and their NHS service use. Study 2 used ethnographic methods to explore experiences of cancer care for people living with dementia. We used interviews, conversations and observations of cancer care to include the perspectives of people living with cancer and dementia, their families, and hospital staff.

**Results:** Study 1 examined the GP records of 166,000 people living with cancer and/or dementia; 7.5% of people with cancer aged ≥75 also had dementia, and similarly 7.5% of people aged ≥75 with dementia also had cancer. Dementia rates amongst people with the ten most common cancers ranged from 1.2% (brain, other CNS and intracranial tumours) to 5% (bladder cancer). People with both conditions attended 1.5 primary care appointments on average per month, compared to 1.1 for those with dementia alone and 1.4 for those with cancer alone. Study 2 identified a number of cancer care challenges for people living with dementia, including recognition of dementia and difficulties around decision-making, care processes and care environments. Families played important and difficult to replicate roles in their relative’s care.

**Conclusion:** This study provides the best available UK estimates of the size, characteristics and cancer care needs of people living with dementia. It highlights areas where hospitals and staff may be able to improve cancer treatment and care experiences for people living with dementia, and areas for further research.

**Acknowledgement:** A version of this abstract has been published previously for the 29^th^ Alzheimer Europe Conference “Making valuable connections”, see https://www.alzheimer-europe.org/Conferences/The-Hague-2019/Detailed-programme-and-abstracts/P22.-Acute-and-hospital-care.

**Disclosure:** Funded by NIHR Research for Patient Benefit, London, UK

**Corresponding author:** Michelle Collinson

### 87. Cancer Together with other Chronic Health conditions (CATCH): understanding population characteristics and healthcare resource use in general practice

#### Michelle Collinson^1^, Ellen Mason^1^, Amanda Farrin^1^, Laura Ashley^2^, Suzanne Richards^3^, Graham Brunt^4^, Jacqui Gath^5^, Margaret Ogden^5^, Claire Surr^5^

##### ^1^Clinical Trials Research Unit, University of Leeds, Leeds, UK, ^2^School of Social Sciences, Leeds Beckett University, Leeds, UK, ^3^Academic Unit of Primary Care, Leeds Institute of Health Research, University of Leeds, Leeds, UK, ^4^Leeds Beckett University Service User and Carer Group, Leeds Beckett University, Leeds, UK, ^5^Centre for Dementia Research Expert by experience, Leeds Beckett University, Leeds, UK

**Background:** Many people living with cancer have other comorbidities e.g. diabetes or depression, leading to treatment and care complexities. Cancer care is routinely provided in secondary care, however comorbidities are managed in primary care. No studies have examined the prevalence of comorbidities using the Quality and Outcome Framework (QOF) or the healthcare useage of people living with cancer and comorbidities in England.

**Method:** CATCH, funded by Macmillan Cancer Support, is a cross-sectional observational study aiming to describe the population size, characteristics and healthcare useage of people living with cancer and comorbidities. Data were obtained from ResearchOne, a database of electronic patient records from English GP practices. Patients ≥50 with a cancer diagnosis consistent with QOF eligibility during 2005-2016 were included. Data included patient socio-demographics, presence of comorbidities and healthcare useage from 391 general practices (5.1% of English practices).

**Results:** Analysis is ongoing. We identified 99,188 people living with cancer; 56% had at least one comorbidity diagnosed in the two years prior to cancer. Hypertension was recorded as the most common comorbidity (22% of patients). More men were living with cancer and a comorbidity than women (54% vs. 46%). 60% of patients living with cancer and without comorbidity attended a primary care appointment in the 12-month period after cancer was recorded in the GP record; this was no different for those with a comorbidity. The average number of appointments attended per month was similar for those with and without comorbidity (1.5 vs. 1.2) however the average number of appointments attended per month increased with the number of comorbidities (up to 2.2 for those with >5 comorbidities).

**Conclusion:** CATCH provides the first estimates of the population size, clinical and healthcare useage characteristics of people living with cancer and QOF eligible comorbidities in England. It highlights the needs of this group and areas for future research.

**Disclosure:** Funded by Macmillan Cancer Support, London, UK

**Corresponding author:** Michelle Collinson

### 88. Genome-wide association study of acute toxicity and quality of life in breast cancer patients treated by surgery and radiotherapy in the REQUITE cohort study

#### Tim Rattay^1^, Colin Veal^1^, David Azria^2^, Jenny Chang-Claude^3^, Susan Davidson^4^, Alison M Dunning^5^, Dirk de Ruysscher^6^, Sara Gutierrez-Enriquez^7^, Philippe Lambin^8^, Anusha Müller^3^, Tiziana Rancati^9^, Barry S. Rosenstein^10^, Petra Seibold^3^, Elena Sperk^11^, R. Paul Symonds^1^, Riccardo Valdagni^9^, Ana Vega^12^, Liv Veldeman^13^, Adam Webb^1^, Catharine West^14^, Christopher J Talbot^1^, On behalf of the^15^

##### ^1^University of Leicester, Leicester, UK, ^2^ Institut du Cancer Montpellier (ICM), Montpellier, France, ^3^German Cancer Research Center (DKFZ), Heidelberg, Germany, ^4^The Christie Hospital NHS Foundation Trust, Manchester, UK, ^5^University of Cambridge, Cambridge, UK, ^6^University Hospitals Leuven, KU Leuven, Leuven, Belgium, ^7^Vall d’Hebron Institute of Oncology, Barcelona, Spain, ^8^MAASTRO Clinic, Maastricht, Netherlands, ^9^ Istituto di Ricovero e Cura a Carattere Scientifico, Fondazione Istituto Nazionale dei Tumori Milan, Milan, Italy, ^10^Icahn School of Medicine at Mount Sinai, New York, US, ^11^University Medical Centre Mannheim, Heidelberg, Germany, ^12^Fundacion Publica Galega Medicina Xenomica, A Coruña, Spain, ^13^Universiteit Ghent, Ghent, Belgium, ^14^University of Manchester, Manchester, UK, ^15^REQUITE Study, Leicester, UK

This abstract has been published previously, https://www.sciencedirect.com/science/article/pii/S0959804918304040?via%3Dihub

**Disclosure:** Funded by EU, NIHR, London, UK

**Corresponding author:** Tim Rattay

### 89. Identifying people with multiple cancer diagnoses – a Scottish Routes from Diagnosis analysis

#### Emily Moore^1^, Cheryl Denny^1^, Kelly Shiell-Davis^2^, Claire LeBlanc^2^

##### ^1^Information Services Division, NHS National Services Scotland, Edinburgh, UK ^2^Macmillan Cancer Research, London, UK

**Background:** There is a significant and increasing minority of people diagnosed with more than one cancer in their lifetimes, due (amongst other factors) to improving diagnostic techniques and increased long-term survival of people living with and beyond cancer. As part of the Scottish Routes from Diagnosis (SRfD) project we investigated pathways for people diagnosed with more than one primary cancer. Here we explore the prevalence of multiple diagnoses and the timing of other diagnoses in relation to the diagnosis of the index cancer.

**Method:** We used routinely collected national data to define cohorts of people diagnosed with the four most common cancers in Scotland (breast, prostate, colorectal and lung cancers) in 2012, and to identify persons with another cancer (excluding multiple primaries of the index cancer) diagnosed up to 10yrs before or 5yrs after the index diagnosis.

**Results:** The proportion of persons with another cancer diagnosis during the total lookback and follow-up periods ranged from 7% (breast cancer cohort) to 13% (prostate cancer cohort). The rate of diagnoses (per person year at risk) varied by time and cohort. The lung cancer cohort had the highest rate of other diagnoses throughout the time period, and breast cancer the lowest.

**Conclusion:** A significant minority of people in our cohorts experienced another cancer diagnosis in addition to their index cancer. The number of persons affected varied by cohort, due to differences in age, common risk factors and survival rates. Since diagnoses tend to cluster in time, people with multiple cancer diagnoses in a short time period may be undergoing treatment for both cancers concurrently – and therefore may have additional support and care needs due to the greater burden of concurrent disease and treatment. People with multiple diagnoses may benefit from increased awareness of this group among providers of care and support services.

**Disclosure:** Funded by Macmillan Cancer Support, London, UK

**Corresponding author:** Emily Moore

